# Mosaic autosomal aneuploidies are detectable from single-cell RNAseq data

**DOI:** 10.1186/s12864-017-4253-x

**Published:** 2017-11-25

**Authors:** Jonathan A. Griffiths, Antonio Scialdone, John C. Marioni

**Affiliations:** 1 Cancer Research UK Cambridge Institute, University of Cambridge, CB2 0RE, Cambridge, UK; 2 EMBL-European Bioinformatics Institute (EMBL-EBI), Wellcome Genome Campus, Cambridge, CB10 1SD UK; 3 Wellcome Trust Sanger Institute, Wellcome Genome Campus, CB10 1SA, Cambridge, UK; 4 Present Address: Institute of Epigenetics and Stem Cells, Helmholtz Zentrum München, München, Germany

**Keywords:** Aneuploidy detection, Single-cell, Copy-number, RNAseq

## Abstract

**Background:**

Aneuploidies are copy number variants that affect entire chromosomes. They are seen commonly in cancer, embryonic stem cells, human embryos, and in various trisomic diseases. Aneuploidies frequently affect only a subset of cells in a sample; this is known as “mosaic” aneuploidy. A cell that harbours an aneuploidy exhibits disrupted gene expression patterns which can alter its behaviour. However, detection of aneuploidies using conventional single-cell DNA-sequencing protocols is slow and expensive.

**Methods:**

We have developed a method that uses chromosome-wide expression imbalances to identify aneuploidies from single-cell RNA-seq data. The method provides quantitative aneuploidy calls, and is integrated into an *R* software package available on GitHub and as an Additional file of this manuscript.

**Results:**

We validate our approach using data with known copy number, identifying the vast majority of aneuploidies with a low rate of false discovery. We show further support for the method’s efficacy by exploiting allele-specific gene expression levels, and differential expression analyses.

**Conclusions:**

The method is quick and easy to apply, straightforward to interpret, and represents a substantial cost saving compared to single-cell genome sequencing techniques. However, the method is less well suited to data where gene expression is highly variable. The results obtained from the method can be used to investigate the consequences of aneuploidy itself, or to exclude aneuploidy-affected expression values from conventional scRNA-seq data analysis.

**Electronic supplementary material:**

The online version of this article (doi:10.1186/s12864-017-4253-x) contains supplementary material, which is available to authorized users.

## Background

Aneuploidies are gains or losses of entire chromosomes. They occur commonly during early human development [1], cause some human disease (Edwards, Patau and Down syndromes), and are implicated in critical failures at the pre-implantation stage of development [2]. While the expression levels of genes on chromosomes with an aneuploidy are buffered in some cases, these mechanisms rarely fully compensate for the additional or missing gene copy and may only act on a gene-by-gene basis [3]. Aneuploidy-driven expression changes have been observed in yeast [4], Drosophila [5], and human cell lines at both the mRNA and the protein level [6].

Previous studies using array Comparative Genomic Hybridisation (aCGH) [1] have shown that aneuploidies that arise during very early embryonic development are frequently mosaic in character, such that the copy number gain or loss only affects a fraction of the cells in the embryo; these are referred to as mosaic aneuploidies. Similarly, mosaic aneuploidy is observed in populations of embryonic stem cells [7]. To properly characterise and investigate mosaic aneuploidy, it is therefore necessary to study individual cells rather than bulk populations.

Technological developments have facilitated the application of genomics techniques at single-cell resolution. This has allowed the genome, transcriptome, epigenome and proteome of individual cells to be molecularly characterised [8–12]. Furthermore, it has recently become possible to combine multiple sequencing modalities and apply them to the same cell: for example, parallel genome and transcriptome sequencing [13] (G&T-seq) has allowed the integration of copy number and mRNA expression information.

Nevertheless, such combined profiling is relatively unusual and most experiments focus on assaying only a single molecular feature. In particular, most published studies have focussed on single-cell RNA-sequencing (scRNA-seq) [8, 14–16]. Consequently, and given the relatively high prevalence of aneuploidies noted above, the ability to call such features directly from scRNA-seq data is highly desirable.

To this end, we have developed a method for calling aneuploidies from scRNA-sequencing data and applied it to a variety of different use cases. Our approach works by statistically identifying, separately for each cell, chromosomes with genes that show consistently deviant expression compared to the same chromosome in other cells. The efficacy of a similar approach has previously been demonstrated using tumour samples [17], where different clonal populations of cells could be visually distinguished. However, that method does not make explicit ploidy calls. Our method shows high levels of sensitivity and specificity when using known copy-number information from G&T-seq data. Moreover, its predictions are supported by allele-specific expression information and differential expression analysis.

## Methods

Let *c*_*gij*_ denote the normalised (Counts Per Million, CPM) expression level for gene *g* on chromosome *i* in cell *j*. Furthermore, let

$$ a_{gij} = \frac{c_{gij}}{\text{med}_{j} c_{gij}} $$

denote the expression of gene *g* on chromosome *i* in cell *j* normalized by the median expression of the same gene across cells. We consider only highly expressed genes (see “Operational information” section, below) to reduce the effects of technical artefacts common to scRNA-seq as well as to prevent occurrence of extreme values of *a*_*gij*_.

Subsequently, for every cell-chromosome combination we define

$$b_{ij} = \sum_{g \in i} a_{gij} $$

*b*_*ij*_ depends on the number of genes *g* considered on chromosome *i*. To make this sum comparable across chromosomes, which contain different numbers of genes, we normalise by the number of considered genes on each chromosome, *G*_*i*_:

$$r_{ij} = \frac{b_{ij}}{G_{i}} $$

Finally, within each cell, we convert this ratio into a score centred at 1 across chromosomes:

$$s_{ij} = 1 + (r_{ij} - \text{med}_{i} r_{ij}) $$

**Fig. 1:**
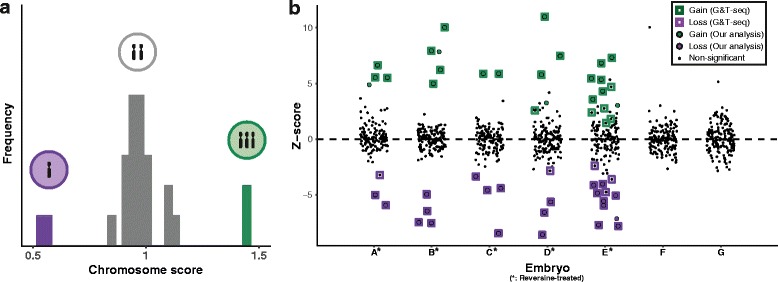
Successful detection of aneuploidies from scRNA-seq data. **a** Overview of the method. Cells with aneuploid chromosomes (purple and green) have altered levels of transcription of genes on the affected chromosome (less and more, respectively). For a given chromosome and cell, we compute a score for how deviant the overall expression of genes on that chromosome is relative to that in other cells. **b** We applied our method to 8-cell stage mouse embryos that were sequenced via a parallel genome and transcriptome method (G&T-seq). Our method performs well compared to the ground truth provided by genomic sequencing (sensitivity 78.0%, specificity 99.5%, FDR 11.4%). The chromosome with high Z-score in embryo F is not called as aneuploid as it does not pass an effect size threshold (“Methods” section)

Assuming that no chromosome within a cell has a copy number gain or loss, *s*_*ij*_ will deviate randomly around 1. By contrast, if specific chromosomes possess evidence of an aneuploidy their scores will be elevated or reduced accordingly. A graphical representation is shown in Fig. 1 a. Note that this interpretation assumes that the majority of chromosomes within a cell are not affected by the same type of aneuploidy.

To infer whether a cell-chromosome displays aberrant copy number, we converted *s*_*ij*_ into a Z-score, where the variance was estimated separately for each chromosome across cells using the median absolute deviation (MAD). We identified aneuploid chromosomes using an FDR-corrected *p*<0.1, where the p-value was obtained using Student’s t-distribution.

### Operational information

As input, we considered all cells that passed quality control using the criteria employed in each analysed dataset.

We only consider highly expressed genes (median CPM>50) to prevent inclusion of genes where small differences in expression across cells could make large differences to *a*_*gij*_.

Before utilising the method, Principal Component Analysis (PCA) was applied to the log-transformed highly expressed genes to identify substructure in the data. The presence of sub-structure will be driven by differentially expressed genes across cells. Consequently, jointly analysing cells in this setting would result in chromosome scores driven by differentially expressed genes rather than aneuploidy. Therefore, if cell groupings were observed, we assigned cells into different groups and analysed them separately.

Finally, to call an aneuploidy we not only required that the corrected p-value was less than 0.1, but also imposed an effect size threshold such that cell-chromosomes where 0.8<*s*_*ij*_<1.2 were not considered significant. This is analogous to approaches commonly applied in microarray and RNA-sequencing analyses when detecting differentially expressed genes.

### Allele-specific expression analysis

To identify biases in allele-specific expression that may be indicative of aneuploidy we considered, for each cell-chromosome, the total number of reads that could be uniquely allocated to one allele. Subsequently, we computed an allele-ratio as the ratio of the total number of reads from one allele over the total number of reads that could be uniquely assigned to either allele. To ensure the median ratio was the same across all chromosomes, we median centred the computed ratios on a per chromosome basis.

At different stages of embryonic development, progressive activation of the paternal genome results in systematically different allele ratios between cells across stages. To ensure that allele ratios were comparable between cells of different stages, we additionally median centred the allele ratios for all chromosomes in each embryo. This step also corrects for further embryo-specific allele-ratio biases.

### Differential expression analysis

To find genes that were associated with aneuploidy, we performed differential expression (DE) analysis using two scRNA-seq datasets (mouse embryos [18] and mESCs [19]). First, we subsetted the data such that it contained only genes that are expressed at a mean level above 10 counts per million (CPM) or more in both datasets. For each dataset, we subsequently performed differential expression analysis using edgeR [20] between cells called as diploid and those that contain at least one chromosome our method called as aneuploid. We added batch (for the mESCs) and embryo number (for the embryos) as covariates to account for technical effects. We called differentially expressed genes as those with an FDR-corrected *p*<0.1.

We considered genes that were either downregulated in both datasets or upregulated in both datasets as a high-confidence set of genes that have altered expression levels in aneuploid cells.

## Results

To detect aneuploidies from scRNA-sequencing data we computed, for a defined group of cells, a score for each chromosome-cell pair that measures whether the expression level of genes in that chromosome differs substantially from other cells. We converted this score into a p-value and used this to detect significant deviations, which we interpret as providing evidence for the presence of an aneuploidy (“Methods” section). The algorithm is available in an *R* package (*scploid*) provided in Additional file 1, with the latest version also available at https://github.com/MarioniLab/Aneuploidy2017.

### True aneuploidies are detected at a low false positive rate

To assess our method’s performance, we first applied it to a dataset where DNA copy number was known. This dataset processed cells from 8-cell stage mouse embryos using a combined genome-and-transcriptome (G&T-seq) strategy [13]. Here, the mRNA and DNA from a single cell are physically separated from one another and processed in parallel to provide transcriptomic and genomic information about the same cell. We therefore used the genomic copy number calls to assess our method’s performance on the transcriptome data.

Grouping cells by treatment (reversine or control), our method identified aneuploidies with a sensitivity of 78.0% (from 50 real aneuploidies) and FDR of 11.4% (Fig. 1 b). Importantly, for chromosomes with no evidence of copy number changes, the p-values derived from the Z-scores were uniformly distributed, suggesting the assumptions made by our model are reasonable (see Additional file 2: Figures S3–S4). Of the 11 false negative aneuploidy calls, 8 were found in the aneuploidy rich embryo E, which showed considerably increased incidence of aneuploidy compared to the other embryos. These chromosomes frequently showed low levels of total expression deviation compared to normal ploidy chromosomes (Additional file 2: Figure S10). By contrast, the 5 false positives were spread across embryos and chromosomes. We did not call any aneuploidies in the control (non-treated) cells, concordant with DNA sequencing copy number calls.

### High gene expression variance confounds our model

**Fig. 2:**
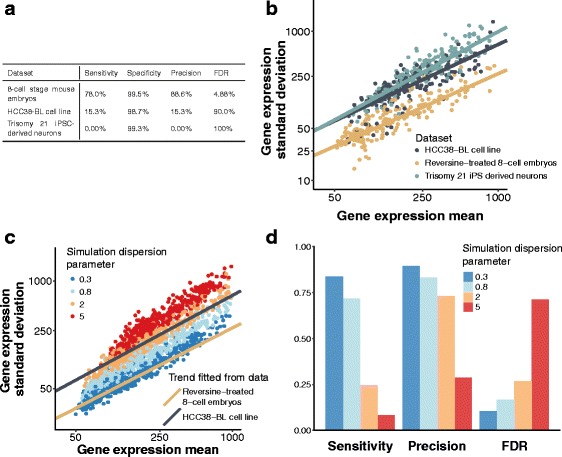
High variability in gene expression levels compromises performance. **a** Our method performs less well on cell-line G&T-sequencing data than on the mouse embryos. All cells were considered for the 8-cell embryos and HCC38-BL data. Trisomy 21 cells were downsampled to a ratio of 1 T21 cell : 4 control cells (normal ploidy chromosome 21), to ensure that these aneuploidies were in the minority and therefore can be detected. **b** The datasets with poor performance show more variable gene expression profiles. For 500 genes selected at random from each dataset (navy: HCC38-BL cell line; yellow: Reversine-treated 8-cell embryos; cyan: trisomy 21 iPS derived neurons) we plot the (log) standard deviation of expression (*y*−axis) against the (log) mean expression (*x*−axis). A linear model was fitted separately for each dataset using genes with a median count (per million reads) of at least 50 and overlaid. **c** Simulated datasets with different dispersion parameters are shown. We simulated four datasets to assess the impact of gene expression variability on the performance of our method. Genes from each simulation are shown and the different dispersion parameters used in the simulation are noted. The regression lines from the fit in **2b** are overlaid. **d** As the data become more variable, the performance of our method degrades. For simulations with variability comparable to the HCC38-BL and Trisomy 21 neuron datasets, the sensitivity and precision are considerably impacted. Reported values are the mean of 10 simulations

In addition, G&T-sequencing was also applied to cells from an immortalized lymphoblastoid cell line, HCC38-BL [13]. Although derived from normal (primarily diploid) cells, G&T-sequencing identified seven copy number changes (three of which affected only one chromosomal arm). Our method identified only two of these changes, albeit the remaining aneuploid chromosomes had scores that were consistent with the change in copy number but not statistically significant. Our method also made several false positive calls. Performance metrics are shown in Fig. 2 a.

To explore what might underpin this observation, we investigated the scRNA-sequencing data derived from these cells in greater detail. Relative to the 8-cell embryo cells, the expression profiles of the HCC38-BL cells were more variable (Fig. 2 b). To investigate how this increased variance might affect the performance of our method, we simulated data based upon the 8-cell stage data introduced earlier. This allowed us to adjust the degree of variability in expression while realistically controlling for both gene expression levels and the relationship between variability and expression (Fig. 2 c, Additional file 2: Section 3.4).

Simulations where the mean-variance relationship was similar to the real 8-cell embryo data yielded performance approximately equal to that of the real data. However, when we increased the level of variability in the expression profiles to that observed in the HCC38-BL cells, we observed a substantial decrease in the performance of our approach (see Fig. 2 d). Similar behaviour was observed in the final set of cells profiled using the G&T protocol, a set of Trisomy 21 and normal ploidy neurons derived from induced pluripotent stem cells (Additional file 2: Section 4).

In sum, the relatively poor performance of our approach when applied to both simulated and real data with higher levels of variance in gene expression levels across cells demonstrates that our ability to detect copy number changes is, unsurprisingly, heavily influenced by the underlying variability in the analysed expression profiles (see “Discussion” section).

### Using allele specific expression to validate copy number calls

It has been reported that the quality of scRNA-seq data derived using joint protocols is less consistent and sometimes lower in quality than when only the mRNA is profiled [21]. Given the relationship between our method’s performance and the amount of noise in the data (explored using the simulations above), we thus applied it to conventional scRNA-seq data. Importantly, we considered scRNA-seq data generated from F1 intercrosses between two inbred strains of mice [18, 19], where each allele is derived from a distinct genetic background.

The presence of an aneuploidy will create an imbalance between the copy numbers of alleles from the affected chromosome, which should lead to an expression imbalance between the two sets of alleles. While this does not provide as definitive a conclusion as DNA sequencing data, a cell that contains such an expression imbalance is nonetheless more likely to contain an aneuploidy. This approach, which considers only the allele-specific expression counts, is orthogonal to our existing method, which considers the total gene expression levels. Therefore agreement between the two approaches would offer support for the efficacy of our method without the use of G&T-seq.

**Fig. 3:**
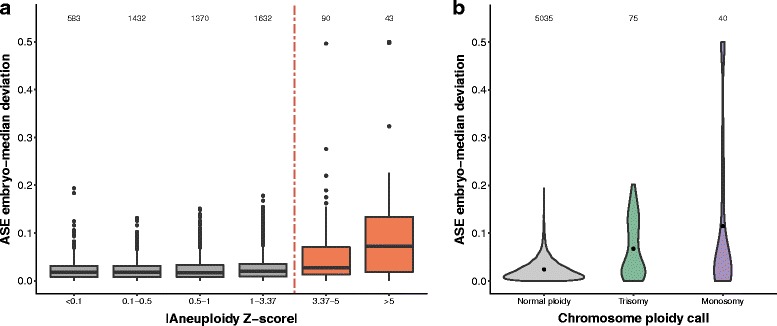
Allele-specific expression datasets support the method’s aneuploidy calls. **a** Chromosomes with higher-confidence aneuploidy calls show greater allele-specific expression (ASE) deviation than cells that are not called (*p*<10^−9^, Mann-Whitney U test). The ASE deviation was corrected for systematic ASE differences for each embryo and for each autosome (Methods). Z-scores to the right of the dashed line are significant after FDR-correction. Values above Z-score bins are the number of chromosomes in each bin. **b** Trisomic and monosomic chromosomes show greater ASE deviation than chromosomes with normal-ploidy (*p*<10^−8^ and *p*<10^−2^, respectively; Mann-Whitney U test). Black circles indicate the mean deviation. Violin plots are area normalised. Values above the plots are the number of chromosomes considered

Specifically, we have considered a set of F1 cells derived from the embryos of C57BL/6J × CAST/EiJ mice (two cell stage to late blastocyst) [18]. First, we applied our aneuploidy detection method on total expression levels to identify candidate aneuploidies. Independently, we considered the allele-specific counts, recording the relative contribution to each chromosome’s total allele-mappable expression from each set of alleles. Chromosomes that score highly in our total expression method show significantly higher levels of deviation from balanced allelic expression than other chromosomes (*p*<10^−9^; Fig. 3 a), thus supporting the validity of our aneuploidy calls. We noted that monosomic-called chromosomes show more severe deviation than those called as trisomic (Fig. 3 b), as expected from the total absence of an allele set in cases of monosomy compared to a smaller change in allele proportion in trisomies. We observed very similar behaviour in another F1 cross dataset of cultured mouse embryonic stem cells [19] (see Additional file 2: Section 6).

### Differential expression analysis identifies genes associated with aneuploidy

To explore the utility of the aneuploidy predictions made by our method, we next performed transcriptome-wide differential expression analysis between all cells called as aneuploid (irrespective of the affected chromosome(s)) and those called as diploid in the two large scRNA-seq datasets analysed above (mouse embryos [18] and cultured mESCs [19]). In total, we identified 22 genes that were commonly upregulated in aneuploid cells in both datasets (“Methods” section & Additional file 2: Section 8; FDR <0.1). No shared downregulated genes were found.

Of these genes, a number show particular relevance for aneuploidy: *Gas5* is a noncoding RNA with roles in growth arrest [22] and apoptosis [23]; *Txnip* overexpression results in G1 cell-cycle arrest [24]; and *Rps27l* overexpression has been shown to promote p53 activity [25], resulting in cell-cycle arrest and apoptosis. Apoptotic and growth arrest functions are known to be associated with aneuploidy [26].

The activation of the unfolded protein response is also linked with aneuploidy [27]. Three of the differentially expressed genes have roles in this pathway: Calnexin (*Canx*), *Pdia3* [28], and *Sdf2* [29]. *Sdf2* differential expression is additionally associated with human oocyte aneuploidy [30].

The roles of many of these differentially expressed genes in aneuploidy-related pathways provides further support for the performance of our method.

## Discussion

Our analysis demonstrates that changes in DNA copy number at the single-cell level can be inferred directly from single-cell RNA-sequencing data. One significant caveat is the relationship between the method’s performance and the degree of noise in the data; such an increase in noise can be driven by several factors. Heterogeneity between cell populations, if not accounted for by clustering cells into homogeneous groups, can lead to systematic chromosome score differences as a result of genes being differentially expressed between populations. Furthermore, cells that are transcriptionally more variable increase the amount of aneuploidy-independent gene expression variance present, which compromises our method’s performance. Such an increase in variance can be driven by technical and biological effects, the former of which is particularly relevant for scRNA-seq, where different protocols generate different amounts of technical noise [31].

Despite this, we demonstrate the good performance of our method across a variety of cell types and conditions. In practice, we suggest that a user exercise caution over the following characteristics of their data. Subpopulations of cells should be identified and analysed separately to reduce aneuploidy-independent expression variance, akin to new approaches for normalising scRNA-seq data [32]. The method should be applied to cells that are relatively phenotypically normal (for example, large genome rearrangements move cells away from integer copy number values and hinder chromosomal gene assignment).

Additionally, as for all scRNA-sequencing analysis approaches, the degree of noise in the data is vital. We have identified three easily computable metrics to assess this: a gene-wise score that captures increased or decreased expression variability compared to the G&T-seq 8-cell embryos; the total number of genes that qualify as highly expressed (median CPM > 50); and the fraction of zero counts observed in these genes — a lower variability score, more available genes, and fewer zeros are features that enable application of our method. Importantly, when applying these metrics to recently generated single-cell RNA-seq datasets [16, 33] we observed that the method appears to be well suited to analyse contemporary scRNA-seq data (see Additional file 2: Section 7 for further detail of these metrics and datasets).

One further concern is whether cells captured at different stages of the cell cycle will lead to differences in copy number driven by variability in the process by which chromosomes are replicated. However, previous work has suggested that dosage control is tightly regulated during the cell cycle [34], mitigating this effect. Additionally, when using an existing classifier to assign the mESCs and developing mouse embryos analysed above to different cell cycle phases [35], we did not observe an association between the degree of aneuploidy and cell cycle (see Additional file 2: Section 9).

Moving forward, it may be possible to integrate more information to increase predictive power. For example, classifiers have previously been trained to identify aneuploid cells based on specific transcriptional signatures [36]. Alternatively, where possible, allele-specific expression information could be coupled with total expression data, which would be especially helpful when detecting copy number deletion events.

Having acquired ploidy information about a single-cell RNA-seq dataset through this method, the results can be exploited in a number of ways to obtain further biological insight. For example, gene expression counts on chromosomes that are suspected to be aneuploid may be excluded when selecting highly variable genes [37] to prevent propagation of aneuploidy-driven signal into downstream analyses. Additionally, as shown above, segregating cell-chromosomes by whether or not they harbour an aneuploidy can help identify genes that are potentially associated with copy number aberrations.

The method may provide particular benefits in stem cell and embryonic research, where aneuploidies are known to be common [2, 7, 38] and single-cell approaches have been widely applied. Additionally, the method is straightforward to apply (requiring no additional experimental work) and easy to interpret, yielding direct aneuploidy calls for each cell-chromosome unlike previous strategies [17]. Application of this method also represents a considerable cost saving compared to expensive and experimentally more complex single-cell genome sequencing protocols [39].

## Conclusions

In this paper, we have shown that chromosome-wide imbalances in mRNA gene expression measured using scRNA-seq can be used to identify aneuploidy. We have demonstrated this using ground-truth ploidy knowledge (parallel genomic & transcriptomic sequencing), allele-specific expression ratios over chromosomes, and differential expression between cells with and without called aneuploidies. The method is straightforward to apply, albeit care must be taken to control for potential confounding factors. Downstream applications include identification of aneuploidies for gaining insight into their cause and / or consequences, as well as to exclude expression values from aneuploid chromosomes to improve the accuracy of common analysis techniques.

## Additional files

Additional file 1 Code for analysis. A gzipped tarball containing all code used for analysis, as well as the.html report referred to above. A package to run aneuploidy assessment in *R* is also included, alongside a script to download the data we have used. The latest version of these files may be found on https://github.com/MarioniLab/Aneuploidy2017. (GZ 6405 kb)

Additional file 2 Analysis report. A.html file that details all the analysis included herein, including additional figures referred to in the manuscript as well as some further analyses. (HTML 8315 kb)

## Abbreviations

aCGH: Array competitive genome hybridisation
CPM: Counts per million
DE: Differential expression
G&T-seq: Genome and transcriptome sequencing
MAD: Median absolute deviation
PCA: Principal components analysis
scRNA-seq: Single-cell RNA sequencing

## References

[1] Vanneste E, Voet T, Le Caignec C, Ampe M, Konings P, Melotte C, Debrock S, Amyere M, Vikkula M, Schuit F, Fryns JP, Verbeke G, D’Hooghe T, Moreau Y, Vermeesch JR (2009). Chromosome instability is common in human cleavage-stage embryos. Nat Med.

[2] Daughtry BL, Chavez SL (2015). Chromosomal instability in mammalian pre-implantation embryos: potential causes, detection methods, and clinical consequences. Cell Tissue Res.

[3] Donnelly N, Storchová Z (2014). Dynamic karyotype, dynamic proteome: buffering the effects of aneuploidy. Biochimica et Biophysica Acta (BBA) - Mol Cell Res.

[4] Torres EM, Sokolsky T, Tucker CM, Chan LY, Boselli M, Dunham MJ, Amon A (2007). Effects of Aneuploidy on Cellular Physiology and Cell Division in Haploid Yeast. Science.

[5] Stenberg P, Lundberg LE, Johansson AM, Rydén P, Svensson MJ, Larsson J (2009). Buffering of Segmental and Chromosomal Aneuploidies in Drosophila melanogaster. PLOS Genet.

[6] Stingele S, Stoehr G, Peplowska K, Cox J, Mann M, Storchova Z (2012). Global analysis of genome, transcriptome and proteome reveals the response to aneuploidy in human cells. Mol Syst Biol.

[7] Gaztelumendi N, Nogués C. Chromosome Instability in mouse Embryonic Stem Cells. Sci Rep. 2014;4. doi:10.1038/srep05324. Accessed 2 Mar 2017 10.1038/srep05324 PMC4060510 24937170

[8] Tang F, Barbacioru C, Wang Y, Nordman E, Lee C, Xu N, Wang X, Bodeau J, Tuch BB, Siddiqui A, Lao K, Surani MA (2009). mRNA-Seq whole-transcriptome analysis of a single cell. Nat Methods.

[9] Bendall SC, Simonds EF, Qiu P, Amir E-aD, Krutzik PO, Finck R, Bruggner RV, Melamed R, Trejo A, Ornatsky OI, Balderas RS, Plevritis SK, Sachs K, Pe’er D, Tanner SD, Nolan GP (2011). Single-cell mass cytometry of differential immune and drug responses across a human hematopoietic continuum. Science (New York, NY).

[10] Smallwood SA, Lee HJ, Angermueller C, Krueger F, Saadeh H, Peat J, Andrews SR, Stegle O, Reik W, Kelsey G (2014). Single-cell genome-wide bisulfite sequencing for assessing epigenetic heterogeneity. Nat Methods.

[11] Buenrostro JD, Wu B, Litzenburger UM, Ruff D, Gonzales ML, Snyder MP, Chang HY, Greenleaf WJ (2015). Single-cell chromatin accessibility reveals principles of regulatory variation. Nature.

[12] Gawad C, Koh W, Quake SR (2016). Single-cell genome sequencing: current state of the science. Nat Rev Genet.

[13] Macaulay IC, Haerty W, Kumar P, Li YI, Hu TX, Teng MJ, Goolam M, Saurat N, Coupland P, Shirley LM, Smith M, Van der Aa N, Banerjee R, Ellis PD, Quail MA, Swerdlow HP, Zernicka-Goetz M, Livesey FJ, Ponting CP, Voet T (2015). G&T-seq: parallel sequencing of single-cell genomes and transcriptomes. Nat Methods.

[14] Islam S, Kjällquist U, Moliner A, Zajac P, Fan JB, Lönnerberg P, Linnarsson S (2011). Characterization of the single-cell transcriptional landscape by highly multiplex RNA-seq. Genome Res.

[15] Kim JK, Kolodziejczyk AA, Ilicic T, Teichmann SA, Marioni JC (2015). Characterizing noise structure in single-cell RNA-seq distinguishes genuine from technical stochastic allelic expression. Nat Commun.

[16] Scialdone A, Tanaka Y, Jawaid W, Moignard V, Wilson NK, Macaulay IC, Marioni JC, Göttgens B (2016). Resolving early mesoderm diversification through single-cell expression profiling. Nature.

[17] Patel AP, Tirosh I, Trombetta JJ, Shalek AK, Gillespie SM, Wakimoto H, Cahill DP, Nahed BV, Curry WT, Martuza RL, Louis DN, Rozenblatt-Rosen O, Suvà ML, Regev A, Bernstein BE (2014). Single-cell RNA-seq highlights intratumoral heterogeneity in primary glioblastoma. Science.

[18] Deng Q, Ramsköld D, Reinius B, Sandberg R (2014). Single-Cell RNA-Seq Reveals Dynamic, Random Monoallelic Gene Expression in Mammalian Cells. Science.

[19] Kolodziejczyk AA, Kim JK, Tsang JCH, Ilicic T, Henriksson J, Natarajan KN, Tuck AC, Gao X, Bühler M, Liu P, Marioni JC, Teichmann SA (2015). Single Cell RNA-Sequencing of Pluripotent States Unlocks Modular Transcriptional Variation. Cell Stem Cell.

[20] McCarthy DJ, Chen Y, Smyth GK (2012). Differential expression analysis of multifactor RNA-Seq experiments with respect to biological variation. Nucleic Acids Res.

[21] Svensson V, Natarajan KN, Ly LH, Miragaia RJ, Labalette C, Macaulay IC, Cvejic A, Teichmann SA (2017). Power analysis of single-cell RNA-sequencing experiments. Nat Methods.

[22] Kino T, Hurt DE, Ichijo T, Nader N, Chrousos GP (2010). Noncoding RNA Gas5 Is a Growth Arrest and Starvation-Associated Repressor of the Glucocorticoid Receptor. Sci Signal.

[23] Mourtada-Maarabouni M, Pickard MR, Hedge VL, Farzaneh F, Williams GT (2008). GAS5, a non-protein-coding RNA, controls apoptosis and is downregulated in breast cancer. Oncogene.

[24] Yamaguchi F, Takata M, Kamitori K, Nonaka M, Dong Y, Sui L, Tokuda M (2008). Rare sugar D-allose induces specific up-regulation of TXNIP and subsequent G1 cell cycle arrest in hepatocellular carcinoma cells by stabilization of p27kip1. Int J Oncol.

[25] Xiong X, Zhao Y, He H, Sun Y (2011). Ribosomal protein S27-like and S27 interplay with p53-MDM2 axis as a target, a substrate and a regulator. Oncogene.

[26] Li M, Fang X, Baker DJ, Guo L, Gao X, Wei Z, Han S, van Deursen JM, Zhang P (2010). The ATM–p53 pathway suppresses aneuploidy-induced tumorigenesis. Proc Natl Acad Sci USA.

[27] Ohashi A, Ohori M, Iwai K, Nakayama Y, Nambu T, Morishita D, Kawamoto T, Miyamoto M, Hirayama T, Okaniwa M, Banno H, Ishikawa T, Kandori H, Iwata K (2015). Aneuploidy generates proteotoxic stress and DNA damage concurrently with p53-mediated post-mitotic apoptosis in SAC-impaired cells. Nat Commun.

[28] Lindquist JA, Jensen ON, Mann M, Hämmerling GJ (1998). ER-60, a chaperone with thiol-dependent reductase activity involved in MHC class I assembly. EMBO J.

[29] Lorenzon-Ojea AR, Guzzo CR, Kapidzic M, Fisher SJ, Bevilacqua E (2016). Stromal Cell-Derived Factor 2: A Novel Protein that Interferes in Endoplasmic Reticulum Stress Pathway in Human Placental Cells. Biol Reprod.

[30] Fragouli E, Bianchi V, Patrizio P, Obradors A, Huang Z, Borini A, Delhanty JDA, Wells D (2010). Transcriptomic profiling of human oocytes: association of meiotic aneuploidy and altered oocyte gene expression. MHR: Basic Sci Reprod Med.

[31] Grün D, Kester L, van Oudenaarden A (2014). Validation of noise models for single-cell transcriptomics. Nat Methods.

[32] Lun ATL, Bach K, Marioni JC (2016). Pooling across cells to normalize single-cell RNA sequencing data with many zero counts. Genome Biol.

[33] Hashimshony T, Senderovich N, Avital G, Klochendler A, de Leeuw Y, Anavy L, Gennert D, Li S, Livak KJ, Rozenblatt-Rosen O, Dor Y, Regev A, Yanai I (2016). Cel-seq2: sensitive highly-multiplexed single-cell rna-seq. Genome Biol.

[34] Padovan-Merhar O, Nair GP, Biaesch AG, Mayer A, Scarfone S, Foley SW, Wu AR, Churchman LS, Singh A, Raj A (2015). Single Mammalian Cells Compensate for Differences in Cellular Volume and DNA Copy Number through Independent Global Transcriptional Mechanisms. Mol Cell.

[35] Scialdone A, Natarajan KN, Saraiva LR, Proserpio V, Teichmann SA, Stegle O, Marioni JC, Buettner F (2015). Computational assignment of cell-cycle stage from single-cell transcriptome data. Methods.

[36] Vera-Rodriguez M, Chavez SL, Rubio C, Pera RAR, Simon C (2015). Prediction model for aneuploidy in early human embryo development revealed by single-cell analysis. Nat Commun.

[37] Brennecke P, Anders S, Kim JK, Kołodziejczyk AA, Zhang X, Proserpio V, Baying B, Benes V, Teichmann SA, Marioni JC, Heisler MG (2013). Accounting for technical noise in single-cell RNA-seq experiments. Nat Methods.

[38] Peterson SE, Westra JW, Rehen SK, Young H, Bushman DM, Paczkowski CM, Yung YC, Lynch CL, Tran HT, Nickey KS, Wang YC, Laurent LC, Loring JF, Carpenter MK, Chun J. Normal Human Pluripotent Stem Cell Lines Exhibit Pervasive Mosaic Aneuploidy. PLoS ONE. 2011;6(8). doi:10.1371/journal.pone.0023018. Accessed 2 Mar 2017 10.1371/journal.pone.0023018 PMC3156708 21857983

[39] Macaulay IC, Teng MJ, Haerty W, Kumar P, Ponting CP, Voet T (2016). Separation and parallel sequencing of the genomes and transcriptomes of single cells using G&T-seq. Nat Protoc.

