# Supplementary material for: Mosaic autosomal aneuploidies are detectable from single-cell RNAseq data
Source: BMC Genomics. 2017 Nov 25;18:904. doi: 10.1186/s12864-017-4253-x (PMC5702132; doi:10.1186/s12864-017-4253-x)
Supplement: Supplementary file 1 — Code for analysis. A gzipped tarball containing all code used for analysis, as well as the.html report referred to above. A package to run aneuploidy assessment in R is also included, alongside a script to download the data we have used. The latest version of these files may be found on https://github.com/MarioniLab/Aneuploidy2017. (GZ 6405 kb) [file 12864_2017_4253_MOESM1_ESM.gz › Aneuploidy2017/PaperSupplementary.html]

Mosaic autosomal aneuploidies are detectable from single-cell RNA-seq data


Code 

- Show All Code
- Hide All Code

# Mosaic autosomal aneuploidies are detectable from single-cell RNA-seq data

Jonathan Griffiths, Antonio Scialdone and John Marioni

#### *23 September 2017*

# Contents

- 1 Method summary
- 2 Analysis on mouse 8-cell stage cells (G&T-seq)
  - 2.1 Testing model assumptions
  - 2.2 False predictions
  - 2.3 Application of Patel et al. method
  - 2.4 Summary
- 3 Analysis on the HCC38/HCC38-BL cell lines (G&T-seq)
  - 3.1 Substructure identification
  - 3.2 HCC38 copy number derivation
  - 3.3 Aneuploidy assessment
  - 3.4 Simulations
    - 3.4.1 Simulating HCC datasets
    - 3.4.2 Separation of library size and overdispersion changes
  - 3.5 Summary
- 4 Analysis on Trisomy 21 neuron cells (G&T-seq)
- 5 Analysis on Deng et al. data (F1 intercross scRNA-seq)
  - 5.1 Allele specific expression (ASE) data
  - 5.2 Comparing our detection method to ASE counts
- 6 Analysis on mESC data (F1 intercross scRNA-seq)
  - 6.1 Finding ASE values
  - 6.2 Comparing our detection method to the ASE counts
  - 6.3 Correspondence to mESC CHR8 aneuploidies
- 7 Testing data suitability for method
  - 7.1 Residual approach
  - 7.2 Number of genes
  - 7.3 Fraction of zeroes
  - 7.4 Summary
- 8 Differential expression analyses
  - 8.1 Differential expression between calls
  - 8.2 Reproduction of aneuploidy markers
- 9 Cell cycle effects do not influence aneuploidy calling
- 10 Manuscript + Supplementary graphics
- 11 R Session Information

This is the primary accompanying document for the paper **Mosaic autosomal aneuploidies are detectable from scRNA-seq data**. It steps through the analysis procedures undertaken, highlighting how we have come to the conclusions made in the manuscript.

The newest versions of thid and other Supplementary Files from the paper can be found at https://github.com/MarioniLab/Aneuploidy2017

# 1 Method summary

First, we load in the functions, packages and data (running *data\_proc.R* and *aneu\_functions.R*).

```
#load in functions
library(here)
folder_location = paste0(here(), "/")
plot_folder = paste0(folder_location, "plots")
source(paste0(folder_location, "aneu_functions.R"))

# To process:
source(paste0(folder_location, "data_proc.R"), echo = FALSE)

# To load:
# for(file in dir(paste0(folder_location, "proc_data/"), full.names = TRUE))
#   load(file)


#stop edgeR/scploid getCounts() issue
getCounts = scploid::getCounts
```

Summarising how our aneuploidy calling method works, we use CPM counts (\(c\_{gij}\)) for gene \(g\) on chromosome number \(i\) in cell \(j\), and normalise the count for each gene by its median value across cells:

\[ a\_{gij} = \frac{c\_{gij}}{\mbox{med}\_{j} c\_{gij}}\]

We then sum these expression scores over each individual chromosome:

\[b\_{ij} = \sum\_{g \in i} a\_{gij}\]

Before controlling for the number of genes \(G\) considered on each chromosome \(i\), \(G\_i\):

\[r\_{ij} = \frac{b\_{ij}}{G\_i}\]

We then centre each cell’s median chromosome score on 1:

\[s\_{ij} = 1 + (r\_{ij} - \mbox{med}\_{i} r\_{ij})\]

This corrects for two features:

1. Monosomies and trisomies release and take up read space respectively in our CPM counts. They therefore increase/depress the summed scores of other chromosomes in the cell. By our assumption that the median scoring chromosome is of normal ploidy, this effect is abolished.
2. Low expression can score from \(0<a\_{gij}<1\), while high expression can score from \(1<a\_{gij}<\infty\). This leads to a slight bias where \(mean\_{j}(a\_{gij}) > 1\).

Assuming that no chromosome within a cell has a copy number gain or loss, \(s\_{ij}\) will deviate randomly around 1. By contrast, if specific chromosomes possess evidence of an aneuploidy their scores will be elevated or reduced accordingly. Note that this interpretation assumes that:

- The median scoring chromosome in a cell must be of normal ploidy. If a majority of chromosomes in a cell show either a trisomy or monosomy, the cell centering step will consider those chromosomes as normal ploidy.
- For each chromosome across cells, its median-scoring chromosome must be normal ploidy. Otherwise, this majority will be median-centered and called as normal ploidy, and the remaining chromosomes will score aberrantly.

To infer whether a cell-chromosome displays aberrant copy number, we convert \(s\_{ij}\) into a Z-score, where the variance was computed separately for each chromosome across cells using the median absolute deviation (MAD), which protects against the variance-inflating influence of aneuploid scores. We identified aneuploid chromosomes using an FDR-corrected \(p<0.1\), where the p-value was obtained using Student’s t-distribution (d.f. \(n\_{cells}-1\)).

This method is motivated by the presence of a whole-chromosome trisomy or monosomy showing systematic transcriptional up- or down-regulation across all of its genes, therefore affecting our chromosome scores relative to the median chromosome score in each cell grouping

We also have a few additional rules for calling aneuploidy to capture effects that the Z-score cannot easily capture:

1. We consider only highly expressed genes (CPM median > 50), as a small expression change for a lowly expressed gene can disproportionately impact the score via the median-normalised counts.
2. We look for substructure in our data using PCA on the highly expressed genes. If any structure is observed, we analyse the cells in each group separately.
3. A chromosome score (\(s\_{ij}\)) must be large or small (>1.2 or <0.8) as well as statistically significant to be called as aneuploid. This prevents calling of aneuploidy in chromosomes whose scores deviate only slightly, but where the variance of the test statistic is small.

# 2 Analysis on mouse 8-cell stage cells (G&T-seq)

A G&T seq dataset of 8-cell stage mouse embryos (published in Macauley et al. 2015) allows us to estimate ploidy from transcriptomes while also having access to the ground truth via parallel genomic sequencing. The concordance between these two sets of information was highlighted in Figure 2 (panel F) of that paper.

First we look for substructure in the data - we do not want our analysis of ploidy confounded by unrelated transcriptional differences, shown in Figure 1

```
#pca on log(counts+1)

plotPCA(emb8, cols = emb8_meta$embryo)
```

Figure 1: 8-cell embryo PCA  
 PCA has been performed on the 8-cell stage embryo data using the genes considered for ploidy assessment (i.e. gene expression median CPM value > 50, log transformed). Colours label different cells.

The reversine treatment appears to drive transcriptional differences between cells - they split on PC1. Different embryos are well mixed, however. We should therefore analyse our cells in groups according to treatment status to avoid introducing systematic chromosome score differences that are driven by the treatment-response rather than any real aneuploidy.

We now call aneuploidies in the mouse embryos using our method and compare our results to known aneuploidies from genome sequencing. Performance metrics are shown in Table 1. The metrics considered are:

- Sensitivity: fraction of real aneuploidies identified.
- Precision: fraction of correctly called aneuploidies of all called aneuploidies.
- Specificity: fraction of correctly called normal ploidy chromosomes.
- False discovery rate (FDR): fraction of incorrectly called aneuploidies of all called aneuploidies.
- Accuracy: fraction of all calls that are correct (aneuploidy and normal ploidy).
- F1: harmonic mean of precision and sensitivity.
- False positive rate (FPR): Fraction of normal ploidy chromosomes called as aneuploid.

```
emb8 = doAneu(emb8)

mouse_hits = getHits(emb8)

mouse_false_pos = getFP(emb8)

#get performance metrics
mouse_test = testPerformance(emb8)
kable(as.data.frame(t(format(mouse_test, digits = 3))), caption = "Method performance on G&T-seq 8-cell embryos")
```

Table 1: Method performance on G&T-seq 8-cell embryos

  
| sensitivity | precision | fdr | specificity | accuracy | f1 | fpr |
| --- | --- | --- | --- | --- | --- | --- |
| 0.78000 | 0.88636 | 0.11364 | 0.99488 | 0.98441 | 0.82979 | 0.00512 |

Performance is excellent, capturing the vast majority of aneuploidies with a low rate of false calling.

Using log-counts (and appropriately altered parameters e.g. CPM threshold) does not improve performance. Results are shown in Table 2

```
#to do this we hack the CPM values and median exclusion method
emb8_log = emb8
emb8_log@cpm = log10(getCounts(emb8_log)+1)
emb8_log = setParam(emb8_log, "min.median", log10(50))
emb8_log = doAneu(emb8_log)

log_test = testPerformance(emb8_log)

kable_tab = rbind(mouse_test, log_test)
rownames(kable_tab) = c("CPM", "Log-CPM")

kable(as.data.frame(kable_tab), caption = "Method performance on G&T-seq 8-cell embryos using log CPMs vs regular method application")
```

Table 2: Method performance on G&T-seq 8-cell embryos using log CPMs vs regular method application

  
|  | sensitivity | precision | fdr | specificity | accuracy | f1 | fpr |
| --- | --- | --- | --- | --- | --- | --- | --- |
| CPM | 0.78 | 0.8863636 | 0.1136364 | 0.9948770 | 0.9844055 | 0.8297872 | 0.0051230 |
| Log-CPM | 0.74 | 0.6981132 | 0.3018868 | 0.9836066 | 0.9717349 | 0.7184466 | 0.0163934 |

Removing the step of normalisation across chromosomes in each cell reduces sensitivity, shown in Table 3.

```
emb8_nocenter = setParam(emb8, "center.cells", FALSE)
emb8_nocenter = doAneu(emb8_nocenter)

nonorm_test = testPerformance(emb8_nocenter)

kable_tab = rbind(mouse_test, nonorm_test)
rownames(kable_tab) = c("Cell normalised", "Not cell normalised")

kable(kable_tab, caption = "Method performance on G&T-seq 8-cell embryos, with and without normalising across chromosomes in a cell")
```

Table 3: Method performance on G&T-seq 8-cell embryos, with and without normalising across chromosomes in a cell

  
|  | sensitivity | precision | fdr | specificity | accuracy | f1 | fpr |
| --- | --- | --- | --- | --- | --- | --- | --- |
| Cell normalised | 0.78 | 0.8863636 | 0.1136364 | 0.9948770 | 0.9844055 | 0.8297872 | 0.0051230 |
| Not cell normalised | 0.66 | 0.7500000 | 0.2500000 | 0.9887295 | 0.9727096 | 0.7021277 | 0.0112705 |

We will now investigate some of the assumptions of our model to ensure it is working as planned.

## 2.1 Testing model assumptions

**Contribution of true aneuploidy status to our scores**

```
# Get the z-scores, and note which chromosomes are truly aneuploid
z_gt = getScores(emb8)
z_gt$truth = FALSE
z_gt$truth[which(paste0(z_gt$cell, z_gt$chr)%in%paste0(getKnownAneu(emb8)$cell, getKnownAneu(emb8)$chr))] = TRUE
#what fraction of score variance is explained by the true presence of an aneuploidy?
z_mod = lm(abs(z_gt$z) ~ z_gt$truth)
```

For Z-scores of each chromosome \(i\), \(Z\_i\), and ploidy status indicator \(\phi\_i\) (\(\phi\_i = 1 \text{ if aneuploid}\), \(\phi\_i = 0 \text{ if normal ploidy}\)), we fit the linear model:

\[|Z\_i| = \alpha + \beta \phi\_i + \epsilon\]

using ordinary least squares. This model explains over half of |Z|-score variance (\(R^2 =\) 0.544).

**Investigation of off-chromosome aneuploidy effects**

Because chromosomes present in “incorrect” copy number show abnormal levels of gene expression, the presence of aneuploid chromosomes may disregulate gene expression on other chromosomes from the same cell. This would confound our prediction of true ploidy status, as we may overpredict the degree of aneuploidy in aneuploid cells due to these off-chromosome effects.

We can check for this by comparing the variance of aneuploidy scores (\(S\_{ij}\)) across known normal ploidy chromosomes in cells with aneuploidies against variance across aneuploidy scores in cells without aneuploidy. This is shown in Figure 2

```
# first we need to scrub the aneuploid chromosomes
# our comparison is between the non-aneuploid ones
scrubbed_ratios = getSijMat(emb8)
known = getKnownAneu(emb8)
for(row in 1:nrow(known)){
    scrubbed_ratios[ as.character(known$chr[row]), 
                     as.character(known$cell[row]) ] = NA
}
#get variance cell-wise
vars = apply(scrubbed_ratios, 2, var, na.rm = T)
aneu_vars = vars[names(vars)%in%getKnownAneu(emb8)$cell]
non_aneu_vars = vars[!names(vars)%in%getKnownAneu(emb8)$cell]

var_test = ks.test(aneu_vars, non_aneu_vars)
wil_test = wilcox.test(aneu_vars, non_aneu_vars)

plot(ecdf(aneu_vars), verticals=TRUE, do.points=FALSE, col = "darkgrey", main = "Ploidy variance cumulative distributions", xlab = "Variance", ylab = "Cumulative distribution")
plot(ecdf(non_aneu_vars), verticals=TRUE, do.points=FALSE, add=TRUE, col='brown')
legend(x=0.012, y=0.4, fill = c("darkgrey", "brown"), legend = c("Aneuploid cell variance", "Non-aneuploid cell variance"))
```

Figure 2: Empirical cumulative distribution functions for aneuploidy score variance  
 The ECDF is plotted for the variance across normal-ploidy chromosomes in each cell for cells that either contain an aneuploidy (grey) or that do not contain an aneuploidy (red). Chromosome scores of truly aneuploid chromosomes are excluded. These distributions investigate whether the presence of an aneuploid chromosome destabilises expression on other chromosomes such that those chromosomes may be incorrectly called as aneuploid.

The difference in variances of non-aneuploid chromosome scores is not significant (P=0.206, KS-test, see cumulative distribution plot above; P=0.12, Mann-Whitney U test).

**Test for properly distributed Z-scores**

We have assumed that chromosome scores are normally distributed with MAD estimated variance, which should give us a set of Z-scores that are t-distributed (\(df = n-1\), \(n\) = group cell size). That this is true is important for our use of p-values to call aneuploidies.

To ensure this is the case, we investigate our observed distribution of normal-ploidy z-scores. We show them compared to the normal distribution (as the number of degrees of freedom is relatively high, at 16) in a Q-Q plot (Figure 3). p-values are also visually evenly distributed, shown in Figure 4.

```
normal_cells = z_gt[!z_gt$truth,]

# We have ties in the KS-test - hide the output that warns of this, as we cannot avoid it in our method (median centering)
ks_test =suppressWarnings( ks.test(normal_cells$z, pt, df = min(table(factor(emb8_meta$treatment)))))


qqnorm(normal_cells$z)
qqline(normal_cells$z)
```

Figure 3: Q-Q plot for non-aneuploid Z-scores  
 The Z-scores calculated from the non-aneuploid chromosomes in the 8-cell embryo data lie close to the diagonal in a normal Q-Q plot.

```
hist(normal_cells$p, xlab = "Unadjusted p-values", main = "")
```

Figure 4: Evenly distributed p-values for non-aneuploid chromosomes  
 P-values calculated for non-aneuploid chromosomes are nearly evenly distributed. The peak at p=1 derives from the median-centering approach used in the method.

The Z scores sit well on the normal Q-Q plot. We can also see that the unadjusted p-values are distributed approximately uniformly (there is an inflation of p=1 values due to our median centering approach.)

The assumptions made in our method therefore appear valid.

## 2.2 False predictions

**False negatives**

```
#collate the failures we make

missing = paste(getFN(emb8)$cell, getFN(emb8)$chr)
extra = paste(getFP(emb8)$cell, getFP(emb8)$chr)

#make them nice to print to the html output
missing = sapply(missing, function(x) paste(read.fwf(textConnection(x), c(2, nchar(x)), as.is = TRUE), collapse = " chr"))

extra = sapply(extra, function(x) paste(read.fwf(textConnection(x), c(2, nchar(x)), as.is = TRUE), collapse = " chr"))


missing_e = getFN(emb8)[grepl("E", getFN(emb8)$cell), ]
true_e = getKnownAneu(emb8)[grepl("E", getKnownAneu(emb8)$cell), ]

tab_true = table(c(true_e$chr, 1:19))-1
tab_missing = table(c(missing_e$chr, 1:19))-1


frac = tab_missing/tab_true
remove = !is.na(frac)

#summary(glm( I(frac[remove]) ~ I(mouse_gpc[remove])))
```

By looking at the false negative predictions we have made (A5 chr5, D7 chr13, D8 chr13, E1 chr7, E1 chr8, E2 chr15, E3 chr4, E4 chr10, E4 chr19, E6 chr8, E6 chr9), we see that the aneuploidy rich embryo E makes up the vast majority of our false negatives (72.7%). This is disproportionately higher than the fraction of real aneuploidies in the dataset that originate from this embryo (44%)

The chromosomes missed in embryo E do not appear to be driven by increased or reduced numbers of considered genes on these chromosomes, shown in Figure 5.

```
enough_counts = rownames(getCPM(emb8))[apply(getCPM(emb8), 1, median) > 50]
mouse_gpc = table(getCHR(emb8)[apply(getCPM(emb8), 1, median) > 50])
mouse_gpc = mouse_gpc[order(as.numeric(names(mouse_gpc)))]

e_counts = getCPM(emb8)
e_counts = e_counts[, grepl("E", colnames(e_counts))]
enough_counts = rownames(e_counts)[apply(e_counts, 1, median) > 50]
keep_genes = apply(e_counts, 1, median) > 50
e_emb_gpc = table(getCHR(emb8)[keep_genes])
e_emb_gpc = e_emb_gpc[order(as.numeric(names(e_emb_gpc)))]


plot(x=as.vector(e_emb_gpc[remove]), y = as.vector(frac[remove]), xlab = "Considered genes per chromosome", ylab = "Fraction of true aneuploidies missed by our method")


mod = glm(frac ~ mouse_gpc, family = "binomial", weights = tab_true)
predo = predict(mod, newdata = data.frame(mouse_gpc = 50:300), type = "response")
points(50:300, predo, col = "darkgrey", type = "l")
```

Figure 5: The number of considered genes per chromosome does not affect calling efficacy  
 For cells in embryo E, we do not see any relationship between the fraction of False Negative predictions and the number of genes considered on each chromosome.

For the fit shown in Figure 5, the number of genes available for aneuploidy prediction did not affect the false negative rate (p=0.473, binomial GLM).

How are these chromosomes escaping our calling method? Below, we show the scores for the false negative predictions in embryo E in Table 4.

```
missing = presentFN(emb8)

kable(missing[grepl("E", missing$cell),], row.names = FALSE, caption="Cells from embryo E incorrectly identified as normal-ploidy. \"monosomy\" indicates whether the chromosomes were monosomic or trisomic. \"score\" indicates the expression score calculated, and \"p.adj\" the associated p-value. \"sig_p\" and \"sig_score\" indicate whether the p-value and expression score are sufficient for aneuploidy call, respectively. \"dir_correct\" indicates whether the expression deviation matches the real aneuploidy.")
```

Table 4: Cells from embryo E incorrectly identified as normal-ploidy  
 “monosomy” indicates whether the chromosomes were monosomic or trisomic. “score” indicates the expression score calculated, and “p.adj” the associated p-value. “sig\_p” and “sig\_score” indicate whether the p-value and expression score are sufficient for aneuploidy call, respectively. “dir\_correct” indicates whether the expression deviation matches the real aneuploidy.

| cell | chr | score | p.adj | monosomy | sig\_p | sig\_score | dir\_correct |
| --- | --- | --- | --- | --- | --- | --- | --- |
| E1 | 7 | 1.1107768 | 0.8550054 | FALSE | FALSE | FALSE | TRUE |
| E1 | 8 | 1.1490350 | 0.1448187 | FALSE | FALSE | FALSE | TRUE |
| E2 | 15 | 1.0549615 | 0.5965775 | FALSE | FALSE | FALSE | TRUE |
| E3 | 4 | 0.8747411 | 0.2666980 | TRUE | FALSE | FALSE | TRUE |
| E4 | 10 | 0.8114079 | 0.0008179 | TRUE | TRUE | FALSE | TRUE |
| E4 | 19 | 0.8280325 | 0.0194268 | TRUE | TRUE | FALSE | TRUE |
| E6 | 8 | 1.1272207 | 0.2771054 | FALSE | FALSE | FALSE | TRUE |
| E6 | 9 | 1.1778087 | 0.0009695 | FALSE | TRUE | FALSE | TRUE |

From these FN results, we see a mixture of cells with significant and non-significant p-values, but none with a significant score. However, a number of the chromosomes do have scores very close to clearing our threshold.

It is surprising that we see so many missed aneuploidy calls in this embryo in particular. Given that the p-value threshold is often unmet, it may be the case that the high prevalence of aneuploidies in this embryo hampers our ability to detect them. In Figure 6 we show the effect of the number of chromosomes called as aneuploid on the estimated MAD across chromosomes in embryo E.

```
#Variance of chromosomes + # aneuploidies
emb_e = getScores(emb8)[grepl("E", getScores(emb8)$cell),]
split_chr = split(emb_e, emb_e$chr)

mads = sapply(split_chr, function(x) mad(x$score))
n_aneu = table(factor(getKnownAneu(emb8)$chr[grepl("E", getKnownAneu(emb8)$cell)], levels = 1:19))

ggplot(data = data.frame(mad = mads, n_aneu = as.numeric(n_aneu)), aes(x = n_aneu, y = mad)) +
  geom_jitter(width = 0.1) +
  theme_bw() +
  labs(x = "Number of aneuploidies in embryo E", y = "MAD across cells") +
  geom_smooth(method = "lm", se = FALSE)
```

Figure 6: Aneuploidy inflence on MAD  
 For chromosomes that posess more aneuploidies in embryo E, we routinely identify a higher median absolute deviation in cells from this embryo.

However, the effect is not seen when we include cells from all Reversine-treated embryos, which is how we assess ploidy in this dataset (Figure 7)

```
#Variance of chromosomes + # aneuploidies
emb_all = getScores(emb8)
split_chr = split(emb_all, emb_all$chr)

mads = sapply(split_chr, function(x) mad(x$score))
n_aneu = table(factor(getKnownAneu(emb8)$chr[grepl("E", getKnownAneu(emb8)$cell)], levels = 1:19))

ggplot(data = data.frame(mad = mads, n_aneu = as.numeric(n_aneu)), aes(x = n_aneu, y = mad)) +
  geom_jitter(width = 0.1) +
  theme_bw() +
  labs(x = "Number of aneuploidies in embryo E", y = "MAD across cells") +
  geom_smooth(method = "lm", se = FALSE)
```

Figure 7: Aneuploidy inflence on MAD  
 For chromosomes that posess more aneuploidies in embryo E, we do not identify a higher median absolute deviation across all Reversine-treated cells

Aneuploidies affecting the MAD do not therefore provide a satisfying explanation of the behaviour observed.

Now we consider the variance of gene expression across cells. We have first set the expression of all genes on truly aneuploid chromosomes to `NA`. We have then renormalised the cells’ expression profiles to CPM counts excluding these chromosomes’ genes. Plotted in Figure 8 is the median-variance relationship for highly expressed genes for cells in Reversine treated embryos.

```
dummy_counts = getCounts(emb8)[,!grepl("[GH]", colnames(getCounts(emb8)))]
known = getKnownAneu(emb8)
for(chr in 1:19){
  for(cell in 1:ncol(getCounts(emb8))){
    if(paste(colnames(dummy_counts)[cell], chr) %in% paste(known$cell, known$chr)){
      genes = which(getCHR(emb8) == chr)
      dummy_counts[genes, cell] = NA
    }
  }
}


#renormalise
dummy_counts = sweep(dummy_counts, 2, colSums(dummy_counts, na.rm = TRUE), "/")*1e6


e_counts = dummy_counts[,grepl("E", colnames(dummy_counts))]
other_counts = dummy_counts[,!grepl("E", colnames(dummy_counts))]
e_med = apply(e_counts, 1, median)
e_var = apply(e_counts, 1, var)
o_med = apply(other_counts, 1, median)
o_var = apply(other_counts, 1, var)

plot_df = data.frame(med = c(e_med, o_med), var = c(e_var, o_var), class = c(rep("E", length(e_med)), rep("A-D", length(o_med))))
ggplot(plot_df, aes (x = med+1, y = var+1, col = factor(class)))+
  geom_point(size = 0.2, alpha = 0.3) +
  scale_x_log10(limits = c(50, max(plot_df$med+1)), breaks = c(50, 100, 1000, 10000), name = "CPM median") + 
  scale_y_log10(name = "CPM variance") +
  geom_smooth(method = "lm") +
  scale_color_manual(values = c("coral", "black"), name = "Embryo") +
  geom_vline(xintercept = 50, col = "darkgrey") +
  theme_bw()
```

Figure 8: Median-variance relationship for embryo E and all other reversine-treated embryos

Embryo E does not appear to be markedly more variable in expression than the other Reversine-treated embryos. This is unlikely to be driving our poorer performance.

An alternative explanation could be that embryo E has responded differently to the Reversine treatment to the other embryos. This does not appear to be observed in the PCA in Figure 1, however.

We have investigated this further by correlating the CPM averages of embryos for the method-considered genes (again excluding aneuploid gene counts) between all pairwise embryo combinations. The correlation coefficients are shown in Figure 9.

```
split_counts = lapply(c("A", "B", "C", "D", "E"), function(x) dummy_counts[, grepl(x, colnames(dummy_counts))])
combs = split(t(combn(5,2)), 1:choose(5,2))

cors = sapply(combs, function(x) cor(rowMeans(split_counts[[x[1]]], na.rm = TRUE), 
                                     rowMeans(split_counts[[x[2]]], na.rm = TRUE), 
                                     use = "complete.obs", method = "spearman"))

contains_e = sapply(combs, function(x) 5%in%x)

cor_df = data.frame(cor = cors, is_e = contains_e)

ggplot(cor_df, aes( x= factor(is_e), y = cor)) +
         geom_boxplot() +
  scale_x_discrete(name = "", labels = c("Correlation excludes\nembryo E", "Correlation includes\nembryo E"))+
  scale_y_continuous(name = "Spearman correlation coefficient") +
  theme_bw()
```

Figure 9: Correlation between mean embryo expression levels  
 Correlation coefficients with embryo E are not notably lower than for other embryos.

```
ttest = t.test(cor_df[cor_df$is_e,"cor"], cor_df[!cor_df$is_e,"cor"])
```

Embryo E’s correlation coefficients are not lower than the other correlation coefficients (p = 0.83), suggesting the cells in the embryo are behaving like the other embryos’ cells.

To investigate whether the failure to detect these aneuploidies in embryo E is likely due to the nature of the data, we have looked at the expression sums (in CPM units) across chromosomes for each cell. Notably, these values are distinct from the \(s\_{ij}\) that we have used to score aneuploidy as they consider all genes, and do not median normalise. These CPM sums are plotted, centred on 0, in Figure 10. We have highlighted False Negative chromosome calls, showing the difference between those who failed to pass the p-value threshold alone, and those that also did not pass the \(s\_{ij}\) deviation threshold.

Notably, for the chromosomes where we have called false negative results (across all embryos), the CPM sums are visibly closer to those of diploid chromosomes than the true positive called chromosomes. This suggests that our method is not introducing any particularly increased rate of error, but that these chromosomes do not show considerable expression deviations due to their aneuploidies.

```
split_counts = split(as.data.frame(getCPM(emb8)[,!grepl("[GH]", colnames(getCPM(emb8)))]), getCHR(emb8))
sums = sapply(split_counts, colSums)

scaled = scale(sums, center = TRUE, scale = FALSE)
melted = melt(scaled)
names(melted) = c("cell", "chr", "val")
melted$emb = sapply(melted$cell, function(x) substr(x, start = 1, stop = 1))
pasted = paste(melted$cell, melted$chr)
pasted_score = paste(getScores(emb8)$cell, getScores(emb8)$chr)


melted$true = pasted %in% paste(getKnownAneu(emb8)$cell, getKnownAneu(emb8)$chr)
melted$sig.p = getScores(emb8)[match(pasted, pasted_score),]$p.adj < getParam(emb8, "p.thresh")
melted$sig.score = abs( getScores(emb8)[match(pasted, pasted_score),]$score - 1 ) > getParam(emb8, "min.deviation")
melted$TN = !melted$true & (!melted$sig.p | !melted$sig.score)
melted$TP = melted$true & melted$sig.p & melted$sig.score
melted$FP = !melted$true & melted$sig.p & melted$sig.score
melted$score_fail = melted$true & melted$sig.p & !melted$sig.score
melted$p_fail = melted$true & !melted$sig.p & melted$sig.score
melted$both_fail = melted$true & !melted$sig.p & !melted$sig.score

melted$col = NA
melted$col[melted$TN] = "TN"
melted$col[melted$TP] = "TP"
melted$col[melted$score_fail] = "fail_score"
melted$col[melted$p_fail] = "fail_p"
melted$col[melted$both_fail] = "fail_both"
melted$col[melted$FP] = "FP"

melted$shape = as.numeric(grepl("fail", melted$col)+1)

#score fails
#"#000000", , , , , "#006FA6", "#A30059",
vals = c("TN" = "darkgrey", 
         "TP" = "grey35", 
         "fail_score" = "#1CE6FF", 
         "fail_p" = "#FF34FF", 
         "fail_both" = "#FF4A46",
         "FP" = "#008941")

names = c("TN" = "TN", 
         "TP" = "TP", 
         "fail_score" = "FN, s n.s.", 
         "fail_p" = "FN, p n.s.", 
         "fail_both" = "FN, p&s n.s.",
         "FP" = "FP")

melted = rbind(melted[melted$TN, ], melted[melted$TP, ], melted[!(melted$TP | melted$TN), ])

ggplot(melted, aes(y = val, x = factor(emb), col = col, shape = factor(shape))) +
  geom_jitter(height = 0, width = 0.2) +
  facet_wrap( ~ melted$chr, ncol = 4) +
  scale_color_manual(values = vals, name= "Aneuploidy prediction\nstate", labels = names) +
  scale_shape_discrete(name = "", labels = c("Other", "FN call")) +
  theme_bw() +
  labs(y = "Centred CPM sums", x = "Embryo")
```

Figure 10: Chromosome expression sums broadly reflect aneuploidy calls  
 Each plot shows the CPM expression sums for one chromosome across cells in the five reversine treated embryos. CPM sums were centred and scaled by chromosome number for readability. Chromosomes are coloured according to the success of our aneuploidy prediction model. Where we do not detect aneuploidies in Embryo E cells, those that failed our p-value threshold show similar expression sums to other non-aneuploid cells.

**False Positives**

Our false positive results do not appear to have any trend (A8 chr6, B8 chr11, D8 chr7, E4 chr12, E4 chr6). The aberrant expression profiles of many of these false positives are also seen in the RPKM plot in the G&T-seq paper and in Figure 10 above; consequently, these seem largely to derive from the data itself rather than something our method is spuriously generating.

## 2.3 Application of Patel et al. method

The `inferCNV` method, first presented in a single-cell paper looking at tumour samples, also leverages single-cell expression data to infer DNA copy-number changes. However, there are some important differences to our method:

- `inferCNV` does not supply any statistic for identifying aneuploidies: rather, it generates graphical output of moving-window scores. This does not provide a satisfying output for the user for rare aneuploidies, since the ploidy state of each cell must be visually assessed. Our method provides a definite output for the user, including calls and confidences (via p-values and chromosome scores).
- `inferCNV` groups cells according to the similarities of their aneuploidy profiles. This is useful in e.g. cancer datasets, where common systematic aberrations are expected across groups of cells, but less so where aneuploidies are expected to occur more stochastically through the data (e.g. the embryos described here). Our method easily identifies aberrations that may be rare in the dataset.
- `inferCNV` runs only on an old version of R, for which it can be hard to find the required packages (unless using the author supplied Docker image). Our method is considerably easier to implement, and has no dependencies.

We have run `inferCNV` on the embryo data described above to highlight the differences: it is not easy to identify all of the aneuploidies that were detected with our method by eye from the `inferCNV` output.

We have used the supplied Docker image to build a container to run `inferCNV`. The commands we have run are found in the folded code below, and the figure output is shown in Figure 11.

```
# ### Outside of the Docker container:
# # Get gene locations
# ftp ftp.ensembl.org (anonymous login)
# cd pub/release-89/gtf/mus_musculus
# mget Mus_musculus.GRCm38.89.gtf.gz
# y
# bye
# gunzip Mus_musculus.GRCm38.89.gtf.gz
# 
# # Copy over our data
# docker cp Mus_musculus.GRCm38.89.gtf ecstatic_jones:/usr/local/src/Mus_musculus.GRCm38.89.gtf
# docker cp proc_data/gt_8cell_data.RData ecstatic_jones:/usr/local/src/gt_8cell_data.RData
# 
# 
# ### Inside of the docker container:
# # Install the inferCNV package into R itself (for the packaged R script)
# tar -czf inferCNV.tar.gz inferCNV/
# R
# install.packages(repos=NULL, "inferCNV.tar.gz")
# q("no")
# 
# #Move files around
# mkdir my_data
# python ./inferCNV/scripts/gtf_to_position_file.py Mus_musculus.GRCm38.89.gtf my_data/mouse_pos.txt
# R
# load("gt_8cell_data.RData")
# write.table(log2(gt_8cell_counts+1), "my_data/expr.tab", row.names = TRUE, col.names = TRUE, sep = "\t", quote = FALSE)
# non_aneu = emb8_meta[!emb8_meta$cell %in% gt_8cell_known_hits$cell,]
# write.table(non_aneu$cell[grepl("A", non_aneu$cell)], file = "my_data/a_ref.csv", quote = FALSE, eol = ",", sep = ",", row.names = FALSE, col.names=FALSE)
# write.table(non_aneu$cell[grepl("G", non_aneu$cell) | grepl("H", non_aneu$cell)], file = "my_data/non_rev_ref.csv", quote = FALSE, eol = ",", sep = ",", row.names = FALSE, col.names=FALSE)
# q("no")
#
# #remove trailing commas from R output
# #thanks to https://unix.stackexchange.com/questions/220576/how-to-remove-last-comma-of-each-line-on-csv-using-linux
# sed -i 's/.$//' my_data/a_ref.csv
# sed -i 's/.$//' my_data/non_rev_ref.csv
# 
#
# # Run script with various settings
# ./inferCNV/scripts/inferCNV.R \
#   --cutoff 4.5 \
#   --noise_filter 0.3 \
#   --output_dir no_ref \
#   --vis_bound_threshold " -1,1" \
#   my_data/expr.tab my_data/mouse_pos.txt
#   
# ./inferCNV/scripts/inferCNV.R \
#   --cutoff 4.5 \
#   --noise_filter 0.3 \
#   --ref my_data/a_ref.csv \
#   --output_dir a_ref \
#   --vis_bound_threshold " -1,1" \
#   my_data/expr.tab my_data/mouse_pos.txt
# 
# ./inferCNV/scripts/inferCNV.R \
#   --cutoff 4.5 \
#   --noise_filter 0.3 \
#   --ref my_data/non_rev_ref.csv \
#   --output_dir gh_ref \
#   --vis_bound_threshold " -1,1" \
#   my_data/expr.tab my_data/mouse_pos.txt
#   
# #consolidate for copying
# mv a_ref* a_ref
# mv gh_ref* gh_ref
# mv no_ref* no_ref
#   
# ### Outside of docker image
# # get out the files from the image
# docker cp ecstatic_jones:/usr/local/src/a_ref ~/Desktop/docker_out/a_ref
# docker cp ecstatic_jones:/usr/local/src/gh_ref ~/Desktop/docker_out/gh_ref
# docker cp ecstatic_jones:/usr/local/src/no_ref ~/Desktop/docker_out/no_ref


knitr::include_graphics(paste0(folder_location, "/infercnv.png"), auto_pdf = FALSE)
```

Figure 11: inferCNV output from 8-cell stage embryo data  
 No cells were labelled as control for inferCNV, as aneuploidies were rare and not systematically affecting groups of cells.

While some aneuploidies are immediately apparent, some are harder to see and may not be identified by visual inspection. There are 50 aneuploidies present in the dataset that should be extracted from the image above.

## 2.4 Summary

Our method is very effective at calling aneuploidy in this dataset, with high sensitivity (78.00%) and low FDR (11.36%) and false positive rate (0.51%).

# 3 Analysis on the HCC38/HCC38-BL cell lines (G&T-seq)

## 3.1 Substructure identification

Another dataset from the same G&T-seq paper allows us to apply our method to a cancer cell line (HCC38) and an immortalised lymphoblastoid cell line (HCC38-BL). The first two principal components of both datasets are plotted in Figure 12.

```
plotPCA(hcc, col = getGroups(hcc))
```

Figure 12: PCA of HCC38 and HCC38-BL G&T-seq cell lines  
 PCA was performed using the expression (log-CPM) of genes that qualify for aneuploidy testing. The two cell lines split clearly on the first principal component

It is apparent that we should treat each cell line separately - they are well separated by PCA on highly expressed genes. This is quite predictable though - they are very different tissues.

Even within the quite broadly spread HCC38-BL cells (in Figure 12), we do not observe substructure when plotted on its own principal components, shown in Figure 13

```
plotPCA(hccbl)
```

Figure 13: PCA of HCC38-BL G&T-seq cell line  
 There does not appear to be any substructure in this set of cells.

## 3.2 HCC38 copy number derivation

The HCC38 cell line’s genome is very heavily rearranged, duplicated, and deleted. As such, there are not two copies of each chromosome present in the cell. Not only is hyperdiploidy rife throughout this cell line, but different parts of different chromosomes are present in different numbers, as visible in this karyogram. This poses a severe challenge to our method - we are very far from two copies of each part of each chromosome, and even further from two copies of each gene! For this reason we cannot reasonably expect to extract meaningful results from this set of data, so we have excluded it from the manuscript. However, we have retained some of our exploratory analysis in this document to reinforce the point.

We used the genomic data from the parallel sequencing of these cells to quantify how much of each “normal” chromosome is present in each cell, taking the mean of the ploidy calls across bins on each chromosome (see Methods section of G&T-seq paper for bin information) weighted by the bin length. Notably, this does not account for where genes sit in each bin.

Chromosomes that have mean copy numbers that are very deviant from the across-cell average could be interpreted as “aneuploid”, and this should be reflected transcriptionally and in our calling method. There are a couple of problems, however:

1. We have not selected regions for gene content. An increase in copy number over a chromosome may not therefore represent an increase of gene expression of genes on that chromosome. Effectively, the relationship between copy number and transcriptional output that our method is based on no longer holds
2. Basal copy number is not constant, so our approach of looking for \(s\_{ij} < 0.8\) or \(s\_{ij} > 1.2\) (i.e. around the new genomic ratios of 0.5 or 1.5 for a copy number variant arounf diploidy) is not well founded here.

In any case, the distribution of these scores is shown in Figure 14

```
boxplot(t(cn_base_5), xlab = "CHR", ylab = "Average CN call")
```

Figure 14: Copy number calls for HCC38-BL cell line

We call as our ‘ground-truth’ those which deviate from the median score for each chromosome by a mean copy number of at least 1. These are highlighted as red squares in Figure 15.

```
chr_medians = apply(cn_base_5, 1, median)
deviance = sweep(cn_base_5, MARGIN = 1, FUN = "-", STATS = chr_medians)

hits = which(abs(deviance)>=1)

cn_change = data.frame(cell = colnames(deviance)[col(deviance)[hits]],
                       chr = rownames(deviance)[row(deviance)[hits]],
                       stringsAsFactors = F)

cn_change$expr_down = deviance[hits]<0

melt_cn = melt(cn_base_5)
calls = melt_cn[paste(melt_cn$Var2, melt_cn$Var1) %in% 
  paste(cn_change$cell, cn_change$chr), ]

boxplot(melt_cn$value ~ melt_cn$Var1, xlab = "CHR", ylab = "Average CN call")
points(x=calls$Var1, y = calls$value, col = "red", pch = 15, cex = 1.5)
```

Figure 15: Identified aneuploid cells from HCC38-BL cell line  
 The red highlighted points differ from the median copy-number call for its chromosome by at least 1, and are identified as aneuploid for our method.

Note that due to the considerable genomic differences in the cancer to a normal diploid cell it is highly likely to be unsuitable for our method, its analysis is present in this document only to demonstrate this point.

## 3.3 Aneuploidy assessment

We now call aneuploidies, and assess our performance over this dataset:

```
hcc = doAneu(hcc)
hcc38 = doAneu(hcc38)
hccbl = doAneu(hccbl)


hcc_test = testPerformance(hcc)


#get the scores calculated for all the true aneuploidies
comparison_bl = presentKnown(hccbl)
#get the scores for 'true' hcc38 aneuplopidies
comparison_38 = presentKnown(hcc38)
```

**HCC38 results**

Unsurprisingly (for the reasons described above) our method does not perform well at predicting chromosome-level copy number changes in the cancer cell line, whose genome is too heavily altered. This is highlighted in Table 5, which shows the method’s results for the red-highlighted chromosomes of Figure 15.

```
kable(comparison_38, row.names = FALSE, caption="Results from aneuploid-calling method on HCC38 cell line \"aneuploidies\". Aneuploidies were detected according to differences in DNA-sequencing copy number calls, but cells are not on average diploid. \"monosomy\" indicates whether the chromosomes were identified as monosomic or trisomic. \"score\" indicates the expression score calculated, and \"p.adj\" the associated p-value. \"sig_p\" and \"sig_score\" indicate whether the p-value and expression score are sufficient for aneuploidy call, respectively. \"dir_correct\" indicates whether the expression deviation matches the real aneuploidy.")
```

Table 5: Results from aneuploid-calling method on HCC38 cell line “aneuploidies”  
 Aneuploidies were detected according to differences in DNA-sequencing copy number calls, but cells are not on average diploid. “monosomy” indicates whether the chromosomes were identified as monosomic or trisomic. “score” indicates the expression score calculated, and “p.adj” the associated p-value. “sig\_p” and “sig\_score” indicate whether the p-value and expression score are sufficient for aneuploidy call, respectively. “dir\_correct” indicates whether the expression deviation matches the real aneuploidy.

| cell | chr | score | p.adj | pred\_monosomy | sig\_p | sig\_score | dir\_correct |
| --- | --- | --- | --- | --- | --- | --- | --- |
| Cell\_28 | 14 | 0.8970022 | 0.9593100 | TRUE | FALSE | FALSE | FALSE |
| Cell\_73 | 20 | 0.7983896 | 0.9580485 | TRUE | FALSE | TRUE | TRUE |
| Cell\_86 | 21 | 1.0027564 | 1.0000000 | FALSE | FALSE | FALSE | TRUE |
| Cell\_61 | 11 | 1.0020782 | 1.0000000 | TRUE | FALSE | FALSE | FALSE |
| Cell\_61 | 14 | 0.8576870 | 0.9096386 | TRUE | FALSE | FALSE | FALSE |
| Cell\_61 | 16 | 1.0502237 | 1.0000000 | FALSE | FALSE | FALSE | TRUE |
| Cell\_61 | 20 | 1.0093872 | 1.0000000 | FALSE | FALSE | FALSE | TRUE |
| Cell\_62 | 16 | 1.2108562 | 0.9593100 | FALSE | FALSE | TRUE | TRUE |
| Cell\_66 | 16 | 0.4481023 | 0.3905014 | TRUE | FALSE | TRUE | TRUE |
| Cell\_50 | 20 | 1.1462670 | 0.9593100 | FALSE | FALSE | FALSE | FALSE |
| Cell\_39 | 14 | 1.4684720 | 0.0063642 | FALSE | TRUE | TRUE | TRUE |
| Cell\_39 | 18 | 0.9638130 | 1.0000000 | FALSE | FALSE | FALSE | TRUE |
| Cell\_49 | 16 | 0.8141319 | 0.9593100 | TRUE | FALSE | FALSE | TRUE |
| Cell\_49 | 20 | 0.9040047 | 0.9942073 | TRUE | FALSE | FALSE | TRUE |
| Cell\_5 | 11 | 1.1024259 | 1.0000000 | FALSE | FALSE | FALSE | TRUE |
| Cell\_5 | 14 | 0.9191043 | 0.9593100 | TRUE | FALSE | FALSE | FALSE |
| Cell\_30 | 20 | 0.8859076 | 0.9593100 | TRUE | FALSE | FALSE | TRUE |
| Cell\_30 | 21 | 1.4563136 | 0.0016586 | FALSE | TRUE | TRUE | TRUE |
| Cell\_30 | 9 | 1.0054840 | 1.0000000 | TRUE | FALSE | FALSE | FALSE |
| Cell\_51 | 3 | 1.1310332 | 0.3956177 | FALSE | FALSE | FALSE | TRUE |

**HCC38\_BL results**

The HCC38-BL cell line contains four chromosome 11 trisomies, two 16q monosomies, and a 16q trisomy. Only two of the chromosome 11 aberrations have been successfully called (although we have not separated expression by chromosome arm). The results of our method on these chromosomes is shown in Table 6.

All of the truly aneuploid chromosomes’ scores have the correct direction of effect (matching expression change with aneuploidy type), and all of sufficient magnitude to pass our \(0.8>s\_{ij}\), \(s\_{ij}>1.2\) threshold; however only two pass our p-value thresholds.

```
kable(comparison_bl, row.names = FALSE, caption = "Results from aneuploid-calling method on HCC38-BL cell line aneuploidies. Aneuploidies were detected according to differences in DNA-sequencing copy number calls. \"monosomy\" indicates whether the chromosomes were identified as monosomic or trisomic. \"score\" indicates the expression score calculated, and \"p.adj\" the associated p-value. \"sig_p\" and \"sig_score\" indicate whether the p-value and expression score are sufficient for aneuploidy call, respectively. \"dir_correct\" indicates whether the expression deviation matches the real aneuploidy.")
```

Table 6: Results from aneuploid-calling method on HCC38-BL cell line aneuploidies  
 Aneuploidies were detected according to differences in DNA-sequencing copy number calls. “monosomy” indicates whether the chromosomes were identified as monosomic or trisomic. “score” indicates the expression score calculated, and “p.adj” the associated p-value. “sig\_p” and “sig\_score” indicate whether the p-value and expression score are sufficient for aneuploidy call, respectively. “dir\_correct” indicates whether the expression deviation matches the real aneuploidy.

| cell | chr | score | p.adj | pred\_monosomy | sig\_p | sig\_score | dir\_correct |
| --- | --- | --- | --- | --- | --- | --- | --- |
| Cell\_82 | 16 | 1.4993379 | 0.3473587 | FALSE | FALSE | TRUE | TRUE |
| Cell\_68 | 11 | 1.4461283 | 0.2031838 | FALSE | FALSE | TRUE | TRUE |
| Cell\_22 | 11 | 1.9022468 | 0.0019676 | FALSE | TRUE | TRUE | TRUE |
| Cell\_70 | 11 | 1.3880770 | 0.3211501 | FALSE | FALSE | TRUE | TRUE |
| Cell\_12 | 11 | 1.6503371 | 0.0349751 | FALSE | TRUE | TRUE | TRUE |
| Cell\_56 | 16 | 0.5815260 | 0.6567007 | TRUE | FALSE | TRUE | TRUE |
| Cell\_79 | 16 | 0.5225957 | 0.4934419 | TRUE | FALSE | TRUE | TRUE |

It is noteworthy that the poor performance of the method is *not* driven by our parameter choice. Plotted in Figures 16 and 17 is the F1 score (harmonic mean of sensitivity and precision) for various parameter settings in the HCC38-BL cells and 8-cell embryos respectively - note the lower values of F1 in the HCC38-BL plot across all settings.

```
#countour map over the two dimensions of threshold limit and p value looking at F1 norm/sens/specificty
p_range = c(0.5, 0.1, 0.05, 0.01, 0.001, 0.0001)
thresh_range = c(0.7, 0.6, 0.5, 0.4, 0.3, 0.2, 0.1, 0)
hcc_contour_mat = matrix(NA, ncol = length(p_range), nrow = length(thresh_range), dimnames = list(thresh_range, p_range))
gt_contour_mat = matrix(NA, ncol = length(p_range), nrow = length(thresh_range), dimnames = list(thresh_range, p_range))
for(i in 1:length(hcc_contour_mat)){
  p_thresh = as.numeric(colnames(hcc_contour_mat)[col(hcc_contour_mat)[i]])
  dev_thresh = as.numeric(rownames(hcc_contour_mat)[row(hcc_contour_mat)[i]])
  
  new_emb8 = emb8
  new_emb8 = setParam(new_emb8, "p.thresh", p_thresh)
  new_emb8 = setParam(new_emb8, "min.deviation", dev_thresh)
  new_emb8 = doAneu(new_emb8)
  
  new_bl = hccbl
  new_bl = setParam(new_bl, "p.thresh", p_thresh)
  new_bl = setParam(new_bl, "min.deviation", dev_thresh)
  new_bl = doAneu(new_bl)
  
  hcc_contour_mat[i] = testPerformance(new_bl)["f1"]
  
  gt_contour_mat[i] = testPerformance(new_emb8)["f1"]
  
}

ggplot(melt(hcc_contour_mat), aes (x = factor(Var2, ordered = TRUE, levels = p_range), y = factor(Var1, ordered = TRUE, levels = thresh_range), fill = value)) +
  geom_tile(col = "grey") +
  scale_fill_gradient2(limits = c(0,1), midpoint = 0.5, low = "#fc8d59", mid = "#ffffbf", high = "#99d594", name = "F1 score") +
  labs(x = "p-value cutoff", y = "Effect size cutoff") +
  theme_bw()
```

Figure 16: The F1 score for our method’s performance is shown across parameter settings for the HCC38-BL cell line  
 For each combination of p-value cutoff (x-axis) and required minimum deviation (y axis, 0.2 corresponds to s<0.8 or s>1.2 for significance) the harmonic mean of sensitivity and specificity is shown. The poor performance of our method is not dependent on parameter settings, as it is low across all combinations.

```
ggplot(melt(gt_contour_mat), aes (x = factor(Var2, ordered = TRUE, levels = p_range), y = factor(Var1, ordered = TRUE, levels = thresh_range), fill = value)) +
  geom_tile(col = "black") +
  scale_fill_gradient2(limits = c(0,1), midpoint = 0.5, low = "#fc8d59", mid = "#ffffbf", high = "#99d594", name = "F1 score") +
  labs(x = "p-value cutoff", y = "Effect size cutoff") +
  theme_bw()
```

Figure 17: The F1 score for our method’s performance is shown across parameter settings for the 8-cell embryos  
 For each combination of p-value cutoff (x-axis) and required minimum deviation (y axis, 0.2 corresponds to s<0.8 or s>1.2 for significance) the harmonic mean of sensitivity and specificity is shown. The method performs considerably better over a wider variety of parameters than for the HCC38-BL data.

Parameter choices for our method (p<0.1, \(s\_{ij}>1.2\) or \(s\_{ij}<0.8\)) were not chosen to maximise fit on the mouse dataset but instead at a conventional value for FDR (0.1) and a value for score deviation to take into account any buffered chromosomes or those with sub 1.5/0.5 values due to chance.

The poor sensitivity may be driven by two differences between the HCC datasets and the mouse embryo data we have used:

1. The cell line transcriptomic data has a considerably smaller library size, shown in Figure 18.

```
# non-normalised
mouse_library = colSums(getCounts(emb8))
hcc38_library = colSums(getCounts(hcc38))
hccbl_library = colSums(getCounts(hccbl))

plot(density(mouse_library), 
     xlim = c(0, max(c(hcc38_library, mouse_library, hccbl_library))), 
     ylim = c(0, max(c(density(hcc38_library)$y, density(mouse_library)$y, 
                       density(hccbl_library)$y))*1.2),
     col = "coral",
    xlab = "Library size pre-normalisation")
points(x=mouse_library, y = rep(0, length(mouse_library)), col = "coral", pch = 20)
abline(v=mean(mouse_library), col = "coral", lty = "twodash")

points(density(hcc38_library), type = "l", col = "darkgrey")
points(x=hcc38_library, y = rep(0.2e-7, length(hcc38_library)), col = "darkgrey", pch = 20)
abline(v=mean(hcc38_library), col = "darkgrey", lty = "twodash")

points(density(hccbl_library), type = "l", col = "cornflowerblue")
points(x=hccbl_library, y = rep(0.4e-7, length(hccbl_library)), col = "cornflowerblue", pch = 20)
abline(v=mean(hccbl_library), col = "cornflowerblue", lty = "twodash")

legend(x=2.7e6, y = 8e-7, legend = c("HCC38", "HCC38-BL", "Mouse 8 cell"), fill = c("darkgrey", "cornflowerblue", "coral"))
```

Figure 18: Cell library sizes  
 Each point is a cell. Solid lines show the density of library size distributions for each dataset. Dashed vertical lines indicate the means of distributions. Both the HCC38-BL and HCC38 cells have considerably smaller library sizes than the 8-cell embryos.

2. The variance of a gene’s expression for a given mean expression is higher for the HCC cell lines than the mouse embryos - particularly in the HCC38-BL cells. This is displayed in Figure 19, where the mean and the standard deviation of expression (CPM) are shown for genes in each dataset. We use CPM rather than raw counts because the aneuploidy method also uses CPM. The linear model fit between the log-values of these mean/sd values for each gene is overlaid. Although all data follow a similar gradient, intercepts are different, with the HCC38-BL data in partiular showing higher levels of standard deviation for all values of mean expression (note the log scale, the difference is large).

```
min_median = 50

#normalised
hcc_sub = getCPM(hcc38)
hcc_sub = hcc_sub[rowMedians(hcc_sub)>min_median,]
bl_sub = getCPM(hccbl)
bl_sub = bl_sub[rowMedians(bl_sub)>min_median,]

rev_sub = getCPM(splitCellsByGroup(emb8)[["Reversine"]])
rev_sub = rev_sub[rowMedians(rev_sub)>min_median,]
con_sub = getCPM(splitCellsByGroup(emb8)[["Control"]])
con_sub = con_sub[rowMedians(con_sub)>min_median,]


hcc_sub_mean = rowMeans(hcc_sub)
hcc_sub_sd = apply(hcc_sub, 1, sd)
bl_sub_mean = rowMeans(bl_sub)
bl_sub_sd = apply(bl_sub, 1, sd)

rev_sub_mean = rowMeans(rev_sub)
rev_sub_sd = apply(rev_sub, 1, sd)
con_sub_mean = rowMeans(con_sub)
con_sub_sd = apply(con_sub, 1, sd)

hcc_lm = lm(log10(hcc_sub_sd) ~ log10(hcc_sub_mean))
bl_lm = lm(log10(bl_sub_sd) ~ log10(bl_sub_mean))
rev_lm = lm(log10(rev_sub_sd) ~ log10(rev_sub_mean))
con_lm = lm(log10(con_sub_sd) ~ log10(con_sub_mean))

#Take an equal number of points from each
# sample_size = min(c(length(bl_sub_mean),
#                     length(hcc_sub_mean),
#                     length(rev_sub_mean),
#                     length(con_sub_mean)
#                     ))

sample_size = 1000


set.seed(42)
bl_rows = sample(length(bl_sub_mean), sample_size)
hcc_rows = sample(length(hcc_sub_mean), sample_size)
rev_rows = sample(length(rev_sub_mean), sample_size)
con_rows = sample(length(con_sub_mean), sample_size)


xlims = c(min(c(rev_sub_mean, con_sub_mean, bl_sub_mean, hcc_sub_mean)),
          max(c(rev_sub_mean, con_sub_mean, bl_sub_mean, hcc_sub_mean)))
ylims = c(min(c(rev_sub_sd, con_sub_sd, bl_sub_sd, hcc_sub_sd)),
          max(c(rev_sub_sd, con_sub_sd, bl_sub_sd, hcc_sub_sd)))

plot_df = data.frame(mean = c(bl_sub_mean[bl_rows], hcc_sub_mean[hcc_rows], rev_sub_mean[rev_rows], con_sub_mean[con_rows]),
                     sd = c(bl_sub_sd[bl_rows], hcc_sub_sd[hcc_rows], rev_sub_sd[rev_rows], con_sub_sd[con_rows]),
                     data = c(rep("HCC38-BL", sample_size), 
                              rep("HCC38", sample_size), 
                              rep("Reversine embryos", sample_size), 
                              rep("Control embryos", sample_size) ))

plot_df = plot_df[sample(nrow(plot_df), nrow(plot_df), replace = FALSE),]

mod_df = t(sapply(list(hcc_lm, bl_lm, rev_lm, con_lm), coef))
rownames(mod_df) = c("HCC38", "HCC38-BL", "Reversine embryos", "Control embryos")
colnames(mod_df) = c("int", "slope")
                     
ggplot(plot_df ,aes (x = mean, y = sd, col = factor(data))) +
  geom_point(size = 0.4) +
  scale_x_log10() + scale_y_log10(breaks = c(20, 50, 10^(2:4))) +
  theme_bw() +
  scale_color_manual(values = c("HCC38" = "coral3", "HCC38-BL" = "coral", "Control embryos" = "cyan", "Reversine embryos" = "cyan3")) +
  geom_abline(data = as.data.frame(mod_df), aes(slope = slope, intercept = int, col = factor(rownames(mod_df))), lwd = 1.5)
```

Figure 19: Gene expression variance is higher in the HCC datasets  
 Each point is a gene, with its mean expression shown on the x-axis, and the standard deviation of its expression on the y-axis. Only genes with a median count above 50 are shown (i.e. those used for aneuploidy assessment). The HCC38 and HCC38-BL cell lines show systematically higher expression variance compared to the 8-cell embryos.

```
#compatability for later sections:
bl_counts = bl_sub
bl_sd = bl_sub_sd
bl_mean = bl_sub_mean
```

Given the large magnitude scores but insignificant p-values from our method for the true aneuploidies in the HCC38-BL data, expression variance appears critical - inflated variability increases the score variance, which pushes p-values upward.

Importantly, this relationship is not due to a difference in number of cells - there are 39 cells in the HCC38-BL data and 38 cells in the Reversine treated mouse embryos.

Before moving on, it is also worth noting that the summed RPKM scores used in the G&T-seq paper (Figure 2 panel C, note the larger scale and increased number of non-aneuploid outliers) also demonstrate a considerably less ‘clean’ expression distribution in the HCC data than the mouse embryos. This is in support of the increased variance as shown above, but visible on the chromosome-level as well as the gene-level.

## 3.4 Simulations

To investigate the effects of these two differences, we simulate count matrices from the well-performing mouse data. We have two approaches to this - either by matching aneuploidy effect sizes from the data, or by setting expression in known aneuploid chromosomes to 1.5 or 0.5 times the normal expression. We simulate from the mouse data in order to ensure we retain characteristics of a single-cell RNAseq dataset (e.g. diversity of gene expression levels)

**When using the data to determine effect sizes, we:**

1. Fit the mean-overdispersion relationship over all genes and cells using a loess regression at the log scale. Overdispersion for each gene \(g\) is estimated by \[ OD\_g = \phi\_g = \frac{\sigma\_g^2}{\mu\_g^2} - \frac{1}{\mu\_g} = CV\_g^2 - \frac{1}{\mu\_g} \] where \(\mu\) indicates the gene’s log10 mean count across cells and \(\sigma\) indicates the log10 standard deviation of counts. Here, an overdispersion value of 0 indicates no overdispersion, while a large value indicates large levels of overdispersion. For the loess fit, \(\Phi(x)\) returns the fitted value for the overdispersion \(\phi\) for mean gene expression \(x\).
2. Choose factors by which to change library size and overdispersion. These are denoted \(\delta\) and \(\zeta\) respectively.
3. Choose a negative binomial distribution mean from which to sample. We generate a new matrix of mean counts for cells \(i\) (\(i\in 1,...,N\)) and genes \(g\), \(\mathbf{M}\), by considering the observed count \(c\_{gi}\):

\[M\_{gi} = \begin{cases}
\frac{\sum\_i{c\_{gi}}}{N} & \text{if normal ploidy}\\
c\_{gi} & \text{if aneuploid}
\end{cases}\]

Which is to say that:

- For normal ploidy cells, we consider the mean \(c\_{gi}\) per gene across the cells. Sampling of new expression values proceeds around this mean according to the dispersion, and we avoid generating data that is overfitted to the existing dataset. To preserve diversity of library size (\(L\_i\)) for these cells, we also scale \(M\_{gi}\)s per cell (over all \(P\) normal ploidy cells) using a term \(\lambda\_i\):

\[\lambda\_i = \frac{P L\_i}{\sum\_{c=1}^PL\_c}\] which simply scales cell sizes around the mean count.

- For cells with an aneuploidy, we retain \(c\_{gi}\) for each cell-gene combination, so that we retain the expression balances for all genes, matching the effect sizes observed in the data. We therefore do not scale for library sizes, i.e.

\[\lambda\_i = 1\] for aneuploid cells.

4. Sample new counts \(\mathbf{C}\) (for all genes \(g\), cells \(i\)) from a negative binomial distribution for each gene-cell combination for the output simulated count matrix \(C\_{gi}\), including any changes to dispersion according to \(\zeta\) and library size with \(\delta\).

\[C\_{gi} \sim NB(\mu = \delta \lambda\_i M\_{gi}, OD = \zeta \Phi(\lambda\_i M\_{gi}))\]

This provides a framework where:

- library sizes and gene expression levels are modelled from real single-cell data
- library sizes and dispersions can be easily altered
- aneuploidy effects (and the effect on other genes in CPM space) are retained from true aneuploidies in real single-cell data.

**When using the fixed 1.5x or 0.5x effects, we:**

1. Fit the mean-overdispersion relationship over all genes and cells using a loess regression at the log scale. Overdispersion for each gene \(g\) is estimated by \[ OD\_g = \phi\_g = \frac{\sigma\_g^2}{\mu\_g^2} - \frac{1}{\mu\_g} = CV\_g^2 - \frac{1}{\mu\_g} \] where \(\mu\) indicates the gene’s log10 mean count across cells and \(\sigma\) indicates the log10 standard deviation of counts. Here, an overdispersion value of 0 indicates no overdispersion, while a large value indicates large levels of overdispersion. For the loess fit, \(\Phi(x)\) returns the fitted value for the overdispersion \(\phi\) for mean gene expression \(x\).
2. Choose factors by which to change library size and overdispersion. These are denoted \(\delta\) and \(\zeta\) respectively.
3. Choose a negative binomial distribution mean from which to sample. For each gene \(g\), we calculate the mean count \(\mu\_g\) from *all* cells’ observed counts.
4. For each gene in each cell, determine whether or not its chromosome is aneuploid. The ploidy state of each gene is recorded with \(\alpha\), where:

\[\alpha = \begin{cases}
1.5 & \text{if trisomic},\\
0.5 & \text{if monosomic},\\
1.0 & \text{if diploid}.
\end{cases}\] which simply scales cell sizes after considering the mean count.

5. Sample new counts (for all genes \(g\), cells \(i\)) from a negative binomial distribution for each gene-cell combination for the output simulated count matrix \(\mathbf{C}\), including any changes to dispersion according to \(\zeta\) and library size with \(\delta\). To preserve diversity of library size (\(L\_i\)) for these cells, we also scale \(\mu\_g\)s per cell (over all \(N\) cells) using a term \(\lambda\_i\):

\[\lambda\_i = \frac{N L\_i}{\sum\_{c=1}^NL\_c}\] Sampling means and overdispersions are altered according to \(\alpha\):

\[ C\_{gi} \sim NB\left(\mu = \delta \alpha\_{gi} \lambda\_i \mu\_{g}, OD = \zeta \Phi(\alpha\_{gi} \lambda\_i \mu\_{g})\right) \]

This provides a framework where:

- library sizes and gene expression levels are modelled from real single-cell data
- library sizes and dispersions can be easily altered
- aneuploidy effects (and the effect on other genes in CPM space) are fixed for each gene around the altered distribution mean of 0.5 or 1.5 times normal ploidy for monosomies and trisomies respectively.

This is analogous to “inserting” aneuploidies into the data with a large effect size, although we match the aneuploidy frequencies to the real data.

We then apply our method to the new count matrices, scoring performance against the aneuploidies known from DNA-sequencing.

An example of the overdispersion fit is shown in Figure 20.

```
emb_fit = get_fit(getCounts(emb8))

plot(x=emb_fit$x, y = emb_fit$y, xlab = "log10 Gene mean expression", ylab = "log10 Gene expression OD")

points(x=seq(from = -2, to = 4, by = 0.1), y = predict(object = emb_fit, newdata = data.frame(x=seq(from = -2, to = 4, by = 0.1))), type = "l", col = "red")
```

Figure 20: Overdispersion fit  
 An example of an overdispersion fit across genes is shown. We use the loess trend line, shown in red, to simulate variance in our data dependent on each gene’s mean expression.

We have removed some outlying overdispersions (\(log\_{10}(OD) < -5\)) in order to ensure stability of the fit for highly-expressed genes, which are the most important for our method.

We used this simulation framework to understand the relatively poorer performance of the HCC38-BL data

### 3.4.1 Simulating HCC datasets

First we apply our “data-learnt” effect size approach, matching the characteristics of each dataset in turn. To match the library size differences, we scaled library sizes by the ratio of library size means in each dataset. To match variability, we altered the overdispersion parameter to align most genes in the mean-standard deviation plots below.

Simulations are run ten times, with mean performance metrics shown. Plots of gene mean-variance behaviour are shown from a single simulation.

**Original mouse 8-cell results:**

```
#we need to separate these or it will be confounded by DE between the two cell types.
raw_bl = getCounts(hccbl)

raw_aneu = getCounts(emb8)[, getCellNames(emb8)%in%getKnownAneu(emb8)$cell]
raw_fine = getCounts(emb8)[, !getCellNames(emb8)%in%getKnownAneu(emb8)$cell]

ds_factor_bl = 1/(mean(colSums(getCounts(emb8))) / mean(colSums(raw_bl)))

od_bl = apply(raw_bl, 1, od_est)
od_mouse = apply(getCounts(emb8), 1, od_est)

od_factor_bl = mean(od_bl, na.rm = T)/mean(od_mouse, na.rm = T)

kable(as.data.frame(t(mouse_test)))
```

| sensitivity | precision | fdr | specificity | accuracy | f1 | fpr |
| --- | --- | --- | --- | --- | --- | --- |
| 0.78 | 0.8863636 | 0.1136364 | 0.994877 | 0.9844055 | 0.8297872 | 0.005123 |

**Control simulation results:**

```
#label
#plot mean-sd
control = get_simulated_counts(dispersion_factor = 0.35, normalise = FALSE)
control_cpm = plot_test(sim_counts = control, real_counts = getCounts(emb8))
```

Figure 21: 8-cell embryo control simulation (learnt effect size) mean-variance plot  
 Simulated genes (coral) show similar characteristics to most genes in the true dataset.

```
#do repeat 10x for stats
control_rep = subsample_repeat_analysis(overdisperse = 0.35, nruns = 10)
kable(as.data.frame(t(apply(control_rep, 2, mean))))
```

| sensitivity | precision | fdr | specificity | accuracy | f1 | fpr |
| --- | --- | --- | --- | --- | --- | --- |
| 0.844 | 0.8763036 | 0.1236964 | 0.9938525 | 0.9865497 | 0.8593296 | 0.0061475 |

The control simulations do not show noticable changes in method performance when compared against the real data.

**HCC38-BL simulation results:**

```
#label
#plot mean-sd
ds_learnt_bl = get_simulated_counts(dispersion_factor = 2, downsample_factor = ds_factor_bl, normalise = FALSE)
ds_learnt_bl_cpm = plot_test(real_counts = raw_bl, sim_counts = ds_learnt_bl)
```

Figure 22: HCC38-BL simulation (learnt effect size) mean-variance plot  
 Simulated genes (coral) show similar characteristics to most genes in the true dataset.

```
#repeat x10 for stats
hccbl_rep = subsample_repeat_analysis(overdisperse = 2, downsample = ds_factor_bl, nruns = 10)
kable(as.data.frame(t(apply(hccbl_rep, 2, mean))))
```

| sensitivity | precision | fdr | specificity | accuracy | f1 | fpr |
| --- | --- | --- | --- | --- | --- | --- |
| 0.27 | 0.7630624 | 0.2369376 | 0.9953893 | 0.960039 | 0.3925096 | 0.0046107 |

In this HCC38-BL simulation, the performance of our method is severely compromised compared to the low-variance simulation above.

We now repeat the process with the large/fixed effect size simulations to demonstrate that these changes in performance are not due to subtle characteristics in the mouse data (e.g. a certain few genes driving the effect).

**Control fixed effect simulation:**

```
set.seed(42)

fixed_control = subsample_simulate_aneuploidy(
      counts = getCounts(emb8),
      downsample_frac = 1,
      overdisperse_factor = 0.5,
      gene_map = getGeneTable(emb8),
      known_aneuploidy = getKnownAneu(emb8)
    )

fixed_control_cpm = plot_test(real_counts = getCounts(emb8), sim_counts = fixed_control)
```

Figure 23: 8-cell embryo control simulation (fixed effect size) mean-variance plot  
 Simulated genes (coral) show similar characteristics to most genes in the true dataset.

```
fixed_control_rep = subsample_repeat_analysis_fixed(nruns = 10, overdisperse = 0.5)

kable(as.data.frame(t(apply(fixed_control_rep, 2, mean))))
```

| sensitivity | precision | fdr | specificity | accuracy | f1 | fpr |
| --- | --- | --- | --- | --- | --- | --- |
| 1 | 0.9941176 | 0.0058824 | 0.9996926 | 0.9997076 | 0.9970297 | 0.0003074 |

**HCC38-BL fixed effect simulation:**

```
fixed_bl = subsample_simulate_aneuploidy(
      counts = getCounts(emb8),
      downsample_frac = ds_factor_bl,
      overdisperse_factor = 2,
      gene_map = getGeneTable(emb8),
      known_aneuploidy = getKnownAneu(emb8)
    )
  
  
fixed_bl_cpm = plot_test(real_counts = raw_bl, sim_counts = fixed_bl)
```

Figure 24: HCC38-BL simulation (fixed effect size) mean-variance plot  
 Simulated genes (coral) show similar characteristics to most genes in the true dataset.

```
fixed_bl_rep = subsample_repeat_analysis_fixed(nruns = 10, overdisperse = 2, downsample = ds_factor_bl)


kable(as.data.frame(t(apply(fixed_bl_rep, 2, mean))))
```

| sensitivity | precision | fdr | specificity | accuracy | f1 | fpr |
| --- | --- | --- | --- | --- | --- | --- |
| 0.974 | 0.818149 | 0.181851 | 0.9885246 | 0.9878168 | 0.8878103 | 0.0114754 |

While we see good performance under this simulation scheme for both settings, perfect performance is lost with the increase in gene expression variability.

### 3.4.2 Separation of library size and overdispersion changes

Now that we have observed the problems from the expression variance above, we can look at each of the library size changes and variability changes in turn. We will focus on the simulations that maintain the 8-cell stage effect sizes here i.e. the “learnt” effect size simulations.

```
od_rep = subsample_repeat_analysis(overdisperse = 2, downsample = 1, nruns = 10)

lib_rep = subsample_repeat_analysis(overdisperse = 0.35, downsample = ds_factor_bl, nruns = 10)
```

**Results from real mouse embryo data:**

```
kable(as.data.frame(t(mouse_test)))
```

| sensitivity | precision | fdr | specificity | accuracy | f1 | fpr |
| --- | --- | --- | --- | --- | --- | --- |
| 0.78 | 0.8863636 | 0.1136364 | 0.994877 | 0.9844055 | 0.8297872 | 0.005123 |

**Results from control simulation**

```
kable(as.data.frame(t(apply(control_rep, 2, mean))))
```

| sensitivity | precision | fdr | specificity | accuracy | f1 | fpr |
| --- | --- | --- | --- | --- | --- | --- |
| 0.844 | 0.8763036 | 0.1236964 | 0.9938525 | 0.9865497 | 0.8593296 | 0.0061475 |

**Results from library size change**

```
kable(as.data.frame(t(apply(lib_rep, 2, mean))))
```

| sensitivity | precision | fdr | specificity | accuracy | f1 | fpr |
| --- | --- | --- | --- | --- | --- | --- |
| 0.848 | 0.8807922 | 0.1192078 | 0.9940574 | 0.9869396 | 0.8633774 | 0.0059426 |

Library size changes alone appear to have very little effect

**Results from overdispersion change**

```
kable(as.data.frame(t(apply(od_rep, 2, mean))))
```

| sensitivity | precision | fdr | specificity | accuracy | f1 | fpr |
| --- | --- | --- | --- | --- | --- | --- |
| 0.244 | 0.6697351 | 0.3302649 | 0.9932377 | 0.9567251 | 0.3461576 | 0.0067623 |

Overdispersion appears to be the factor that strongly affects the efficacy of our method.

## 3.5 Summary

Compared to the excellent performance on the mouse 8-cell stage embryos, we see both compromised sensitivity and false positive calling rates in the HCC38-BL data. Despite this, it was seen that the scores of the truly aneuploid scores were still extreme.

Observing that the gene expression variance in these HCC38-BL cells was increased, we have demonstrated via simulations that an increased expression variance degrades the performance of the method, which accounts for the poor performance of the method on the HCC38-BL cells. This also makes sense intuitively, as additional expression variability that is independent from the true ploidy status of a chromosome will compromise the method’s performance.

# 4 Analysis on Trisomy 21 neuron cells (G&T-seq)

This is the final G&T-seq dataset produced in the Macaulay et al. paper. First, we will look for the expected T21/non-T21 substructure implicit in the experimental design, shown in Figure 25.

```
plotPCA(t21, col = getGroups(t21))
```

Figure 25: Principal components of T21 G&T cells  
 The first two principal components, based on the expression of genes used for aneuploidy calling, are shown for the cells in the Trisomy 21 neuron dataset. The T21/normal ploidy cells do not split clearly on PC1, though neither are they fully mixed.

There is PC1 separation by T21 status, but there is also some mixing between the cell types.

Now, we can see if the expression mean-SD relationship will present the same problems in this dataset as in the HCC data. The relationship is shown in Figure 26

```
test_median = 50

tris21 = getCPM(splitCellsByGroup(t21)[["T21"]])
norm = getCPM(splitCellsByGroup(t21)[["Diploid"]])

t21_highexp = tris21[apply(tris21, 1, median)>test_median, ]
norm_highexp = norm[apply(norm, 1, median)>test_median, ]

t21_mean = apply(t21_highexp, 1, mean)
t21_sd = apply(t21_highexp, 1, sd)
norm_mean = apply(norm_highexp, 1, mean)
norm_sd = apply(norm_highexp, 1, sd)

t21_lm = lm(log10(t21_sd) ~ log10(t21_mean))
norm_lm = lm(log10(norm_sd) ~ log10(norm_mean))

sample_size = 1000
t21_sample = sample(length(t21_mean), sample_size)
norm_sample = sample(length(norm_mean), sample_size)
rev_sample = sample(length(rev_sub_mean), sample_size)
con_sample = sample(length(con_sub_mean), sample_size)


plot_df = data.frame(mean = c(t21_mean[t21_sample], norm_mean[norm_sample], rev_sub_mean[rev_sample], con_sub_mean[con_sample]),
                     sd = c(t21_sd[t21_sample], norm_sd[norm_sample], rev_sub_sd[rev_sample], con_sub_sd[con_sample]),
                     data = c(rep("T21", sample_size), 
                              rep("Diploid21", sample_size), 
                              rep("Reversine embryos", sample_size), 
                              rep("Control embryos", sample_size) ))

plot_df = plot_df[sample(nrow(plot_df), nrow(plot_df), replace = FALSE),]

mod_df = t(sapply(list(t21_lm, norm_lm, rev_lm, con_lm), coef))
rownames(mod_df) = c("T21", "Diploid21", "Reversine embryos", "Control embryos")
colnames(mod_df) = c("int", "slope")
                     


ggplot(plot_df ,aes (x = mean, y = sd, col = factor(data))) +
  geom_point(size = 0.4) +
  scale_x_log10() + scale_y_log10(breaks = c(20, 50, 10^(2:4))) +
  theme_bw() +
  scale_color_manual(values = c("T21" = "orchid3", "Diploid21" = "orchid4", "Control embryos" = "cyan", "Reversine embryos" = "cyan3"), name = "") +
  geom_abline(data = as.data.frame(mod_df), aes(slope = slope, intercept = int, col = factor(rownames(mod_df))), lwd = 1.5) +
  labs(x = "Expression mean", y = "Expression S.D.")
```

Figure 26: Gene expression variance is very high in the T21 neuron dataset  
 Each point is a gene, with its mean expression shown on the x-axis, and the standard deviation of its expression on the y-axis. Only genes with a median count above 50 are shown (i.e. those used for aneuploidy assessment). The Trisomy 21 and control neuron cell lines show systematically higher expression variance compared to the 8-cell embryos, at approximately the same or higher variance as the HCC38-BL cell line.

We see a similar mean-variance inflation as the HCC38-BL cells. Additionally, the variance increase is found in both cell types rather than being driven by conflation of two different cell states.

We expect the high levels of variability to prevent our method from working properly, which would result poor performance for this dataset. To test this, we focussed on the Trisomy 21 aneuploidies in these cells.

If we consider all the cells in this dataset together, we will have approximately the same number of chromosome 21 trisomies as normal ploidy cells, which will produce a problem for our method - the median scoring chromosome 21 is not necessarily normal ploidy. We therefore consider all the control cells (20) and a fraction of the Trisomy 21 cells (a ratio of 4 normal : 1 trisomy). We have also included the other aneuploidies visible in the figures 2G and 2H of the G&T-seq paper, using the supplementary data from that figure, and defining as aneuploid those chromosomes with a mean copy number difference of at least 0.5 from 2 across bins (i.e. CN>2.5 or CN<1.5). This represents a copy number variant of more than half of the chromosome. The vast majority of aneuploidies captured are at integer copy number values.

The efficacy of our method on these aneuploidies is shown in Table 7

```
set.seed(42)
keep = c(which(grepl("Control", getCellNames(t21))), 
         sample(which(grepl("T21", getCellNames(t21))), 
                round(sum(grepl("Control", getCellNames(t21)))/4)))

t21_sub = makeAneu(counts = getCounts(t21)[, keep], 
               genes = getGenes(t21), 
               chrs = getCHR(t21), 
               cellNames = getCellNames(t21)[keep], 
               cellGroups = rep("Together", length(keep)))
t21_sub = setKnownAneu(t21_sub, getKnownAneu(t21)[getKnownAneu(t21)$cell %in% getCellNames(t21_sub),])
t21_sub = doAneu(t21_sub)


kable(as.data.frame(t(testPerformance(t21_sub))), caption = "Method performance on Trisomy 21 and control neurons")
```

Table 7: Method performance on Trisomy 21 and control neurons

  
| sensitivity | precision | fdr | specificity | accuracy | f1 | fpr |
| --- | --- | --- | --- | --- | --- | --- |
| 0 | 0 | 1 | 0.9933333 | 0.9675325 | 0 | 0.0066667 |

Performance is very poor again, as in the HCC38-BL data.

p-values are universally high, though we frequently see high aneuploidy scores, shown in Table 8.

```
kable(presentKnown(t21_sub), row.names = FALSE, caption = "Results of our method on the true aneuploidies in Trisomy 21 G&T dataset. \"monosomy\" indicates whether the chromosomes were identified as monosomic or trisomic. \"score\" indicates the expression score calculated, and \"p.adj\" the associated p-value. \"sig_p\" and \"sig_score\" indicate whether the p-value and expression score are sufficient for aneuploidy call, respectively. \"dir_correct\" indicates whether the expression deviation matches the real aneuploidy.")
```

Table 8: Results of our method on the true aneuploidies in Trisomy 21 G&T dataset  
 “monosomy” indicates whether the chromosomes were identified as monosomic or trisomic. “score” indicates the expression score calculated, and “p.adj” the associated p-value. “sig\_p” and “sig\_score” indicate whether the p-value and expression score are sufficient for aneuploidy call, respectively. “dir\_correct” indicates whether the expression deviation matches the real aneuploidy.

| cell | chr | score | p.adj | pred\_monosomy | sig\_p | sig\_score | dir\_correct |
| --- | --- | --- | --- | --- | --- | --- | --- |
| Control\_Cell\_17 | 20 | 1.0104012 | 0.9974284 | FALSE | FALSE | FALSE | TRUE |
| Control\_Cell\_17 | 21 | 1.3229369 | 0.8871549 | FALSE | FALSE | TRUE | TRUE |
| Control\_Cell\_41 | 22 | 0.5501153 | 0.9672639 | TRUE | FALSE | TRUE | FALSE |
| Control\_Cell\_42 | 13 | 1.0651870 | 0.9974284 | FALSE | FALSE | FALSE | TRUE |
| Control\_Cell\_54 | 1 | 1.2159462 | 0.8780198 | FALSE | FALSE | TRUE | FALSE |
| Control\_Cell\_54 | 10 | 1.4021824 | 0.9788983 | FALSE | FALSE | TRUE | TRUE |
| Control\_Cell\_54 | 12 | 0.8674441 | 0.9671060 | TRUE | FALSE | FALSE | FALSE |
| Control\_Cell\_54 | 13 | 1.0542185 | 0.9974284 | FALSE | FALSE | FALSE | TRUE |
| Control\_Cell\_54 | 3 | 1.0303548 | 0.9974284 | FALSE | FALSE | FALSE | FALSE |
| T21\_Cell\_73 | 21 | 1.5662583 | 0.7567055 | FALSE | FALSE | TRUE | TRUE |
| T21\_Cell\_64 | 21 | 1.7008775 | 0.6825309 | FALSE | FALSE | TRUE | TRUE |
| T21\_Cell\_16 | 21 | 1.6922988 | 0.6825309 | FALSE | FALSE | TRUE | TRUE |
| T21\_Cell\_52 | 21 | 1.4968202 | 0.8064159 | FALSE | FALSE | TRUE | TRUE |
| T21\_Cell\_39 | 21 | 2.4889528 | 0.1228236 | FALSE | FALSE | TRUE | TRUE |
| T21\_Cell\_39 | 22 | 1.8126108 | 0.4985160 | FALSE | FALSE | TRUE | FALSE |
| T21\_Cell\_27 | 21 | 1.0083320 | 0.9672639 | FALSE | FALSE | FALSE | TRUE |

This data has shown a more severe degree of variance than the HCC38-BL data according to our mean-SD plots.The performance is also worse than the HCC38-BL data. This result therefore supports our hypothesis that increased expression variability impacts the performance of our method.

# 5 Analysis on Deng et al. data (F1 intercross scRNA-seq)

A study by Deng et al. used CAST \(\times\) BL/6 \(F\_1\) mouse crosses to investigate allele specific expression (ASE) at the single-cell level. We can use the allele-specific reads that have been mapped by the authors to try to estimate where there are aneuploidies - we should see imbalances in expression between all assessable alleles if there is a copy number change.

We have removed some early stages from the data as they are completely dominated by maternal RNA, and we have also excluded the non-embryonic tissues (liver, cultured fibroblasts).

As far as we are aware, this paper does not cover its QC process once reads are aligned. We therefore consider all cells that were made available in the data accompanying the paper. It is notable that all cells look of reasonably high quality when considering the library sizes and number of genes detected, as shown in Figure 27

```
ggplot(data = data.frame(lib = colSums(getCounts(deng)), ngenes = colSums(getCounts(deng)>0), stage = deng_meta$stage)) + 
  geom_point(mapping = aes(x = lib, y = ngenes, col = stage)) +
  labs(x = "Library Size", y = "Number of detected genes") +
  scale_x_log10() +
  scale_color_brewer(palette = "Set1") +
  theme_bw()
```

Figure 27: Deng data QC overview  
 The library size and number of observed genes is plotted for each cell. There are no outlying cells with vastly smaller library sizes, or a discordant number of detected genes, so we retain all cells for further analysis.

Additionally, the authors had tested the cell-libraries for quality before sequencing.

## 5.1 Allele specific expression (ASE) data

Plotted in Figure 28 is the fraction of total reads that CAST alleles contribute to each chromosome, considering only where reads can be distinguished between the two strains’ alleles. The cells are grouped according to their developmental stage, with embryo age increasing along the x axis.

```
ratios = score_ase(count1 = cast_counts, count2 = c57_counts, chr_map = getGeneTable(deng))

#want to remove the fibroblast and liver tissues
ratios = ratios[!ratios$stage %in% c("BXC", "fibroblast"),]
#order by stage
dev_order = c("mid2cell", "late2cell", "4cell", "8cell", "16cell", "earlyblast", "midblast", "lateblast")
ratios = ratios[order(match(ratios$stage, dev_order)),]
ratios$index = 1:nrow(ratios)

#insert a gap between cell groupings to make the plot easier to read
unq_stage = unique(ratios$stage)
stage_loc = sapply(ratios$stage, function(x) which(unq_stage == x))
adjust_multiple = stage_loc-1
shift_size = 100

ratios$new_index = ratios$index + stage_loc*shift_size 
ratios$embryo = sapply(as.character(ratios$cell), function(x) deng_meta$emb_num[deng_meta$cell==x])

#split scores by cell to make it easier to extract outliers
cell_split = split(ratios, ratios$cell)

meanscores = sapply(cell_split, function(x) mean(x$ratio))
highcell = names(meanscores)[which(meanscores>0.95)]
lowcell = names(meanscores)[which(meanscores<0.35)]
highishcells  = names(meanscores)[which(meanscores>0.6)]

danger1 = c(paste(deng_meta$cell[deng_meta$emb_num==2 & deng_meta$stage=="lateblast"], 18) )
danger2 = paste(deng_meta$cell[deng_meta$emb_num==5 & deng_meta$stage=="8cell"], 7)
  
#assign colours to points
colours = ifelse(ratios$cell %in% c(highcell, lowcell), "coral", "black")
colours[which(ratios$stage=="8cell" & ratios$cell%in%highishcells)] = "cornflowerblue"
colours[which(paste(ratios$cell, ratios$chr) %in% c(danger1, danger2))] = "seagreen4"
ratios$col = colours

black_col = ratios[ratios$col=="black",]
other_col = ratios[ratios$col!="black",]

ggplot(ratios, aes( x=new_index, y = ratio)) +
  geom_point(data = ratios[ratios$col=="black",], mapping =aes( x=new_index, y = ratio), alpha = 0.4) +
  geom_point(data = ratios[ratios$col!="black",], mapping =aes( x=new_index, y = ratio), col = ratios[ratios$col!="black","col"], size = 2.5) +
  scale_x_continuous(breaks = c(), name = "Cell-chromosome combination") +
  labs (y = "CAST read fraction") +
  theme_bw()
```

Figure 28: Allele read fractions  
 The CAST allele read fractions for each chromosome in each cell are plotted. Chromosomes are grouped by cell stage, with embryo age increasing rightwards. Several points are highlighted in colour: these are described in the text following this Figure.

There are four key things to notice in the plot:

1. The maternal bias is still visible in the early stages we have considered, as observed on the left of Figure 28. Reads are not entirely saturated by maternal transcripts however, so we will retain all of the plotted stages for analysis. Additionally, we will need to be able to take into account the different baseline ASE imbalances for each stage in our analysis that follows.
2. There are two clearly outlying cells, marked in coral, with one in the 8-cell stage and another in the 16 cell stage. They show respectively a large paternal and maternal bias that we do not think is representative of aneuploidy, as we would not expect aneuploidy to be present in every chromosome of a cell and for such a cell to be viable. Instead, there may be some failure of the cell to correctly activate and balance paternal allelic expression, as suggested in the Supplementary Information of the Deng et al. paper. We will remove these cells from the analysis due to their aberrant allele-specific expression behaviour.
3. There is a set of cells from one embryo only that shows a considerable maternal bias compared to other cells at the same stage. These chromosomes are marked in blue.
4. There are two further embryo-chromosome combinations that show aberrant behaviour, highlighted in the Deng et al. Supplementary Text. These are highlighted in green.

In light of the embryo and stage specific effects, we should not use the raw allele expression fractions for our analysis. Instead, we should correct for stage and embryo driven differences.

Our smallest embryo contains two cells with 19 chromosomes each - 38 allele ratios. We therefore will score every ratio as a deviation from the median allele ratio in each embryo - this should abolish both the early embryo deviation, and the aberrant 8-cell embryo, while not relying on a median of very few ratios. Corrected ratios are shown in Figure 29

```
ratios$ratio_adj_emb = NA

for(row in 1:nrow(ratios)){
  sub_ratios = ratios$ratio[ratios$stage == ratios$stage[row] &
                              ratios$embryo == ratios$embryo[row] ]
  sub_median = median(sub_ratios)
  
  ratios$ratio_adj_emb[row] = ratios$ratio[row] - sub_median + 0.5
}


ggplot(ratios, aes( x=new_index, y = ratio_adj_emb)) +
  geom_point(data = ratios[ratios$col=="black",], mapping =aes( x=new_index, y = ratio_adj_emb), alpha = 0.4) +
  geom_point(data = ratios[ratios$col!="black",], mapping =aes( x=new_index, y = ratio_adj_emb), col = ratios[ratios$col!="black","col"]) +
  labs(x = "Index", y = "CAST read fraction (embryo adjusted)") +
  theme_bw()
```

Figure 29: Allele read fractions, embryo-bias corrected  
 The CAST allele read fractions for each chromosome in each cell are plotted, including a correction to center each embryo’s median chromosome fraction at 0.5. Chromosomes are grouped by cell stage, with embryo age increasing to the right. Coloured cells are as described above.

```
#now we remove those bad cells
ratios = ratios[!ratios$cell%in%c(highcell, lowcell),]
ratios = ratios[!paste(ratios$cell, ratios$chr) %in% c(danger1, danger2), ]
```

This appears to have corrected embryo biases as planned. We now exclude the two aberrant cells (coral) and the two embryo-chromosome combinations (green) that were highlighted in the Deng et al. Supplementary information.

However, there is one other deviation that we should control for - systematic chromosomal ASE imbalances, shown in Figure 30

```
boxplot(ratios$ratio_adj_emb ~ ratios$chr, ylim = c(0.4, 0.6), xlab = "CHR", ylab = "CAST read fraction (embryo adjusted)")
abline(h = 0.5, lty = "twodash", col = "darkgrey")
```

Figure 30: CAST allele fraction scores per chromosome  
 Different chromosomes exhibit systematically different ASE imbalances, perhaps due to imprinting of particular genes.

We will now adjust these by addition of a constant such that the median CAST fraction for each chromosome is 0.5, as shown in Figure 31.

```
ratios$ratio_adj = NA

for(chr in 1:19){
  median_score = median(ratios$ratio_adj_emb[ratios$chr==chr])
  ratios$ratio_adj[ratios$chr==chr] = 0.5 - median_score +
                  ratios$ratio_adj_emb[ratios$chr==chr]
}

boxplot(ratios$ratio_adj ~ ratios$chr, ylim = c(0.4, 0.6), xlab = "CHR", ylab = "CAST read fraction (embryo-CHR adjusted)")
abline(h = 0.5, lty = "twodash", col = "darkgrey")
```

Figure 31: CAST allele fraction scores per chromosome, with correction  
 Different chromosomes exhibit systematically different ASE imbalances, perhaps due to imprinting of particular genes. We have adjusted the scores by chromosome such that the median score is 0.5, as seen here.

Having acquired a measure of chromosome-wide allelic expression imbalances, we will now pivot towards the application of our method.

First, we look for substructure in the highly expressed genes to identify how we should group the application of our aneuploidy method, shown in Figure 32.

```
deng_meta$emb_num = as.character(deng_meta$emb_num)

plotPCA(deng)
```

Figure 32: Principal components of mouse embryonic cells (Deng et al)  
 The first two principal components, calculated from the genes used for aneuploidy calling, are shown for the cells in the Deng et al. dataset. Between PC1 and PC2, all stages have split.

We should analyse by stage, it seems.

We will now investigate the mean-variance relationship in this data. We have chosen two of the largest cell populations to plot, shown in Figure 33. We use two populations to prevent differential gene expression artificially increasing expression variance.

```
eight_counts = getCPM(deng)[,deng_meta$stage=="8cell"]
eight_mean = rowMeans(eight_counts)[rowMedians(eight_counts)>50]
eight_sd = apply(eight_counts, 1, sd)[rowMedians(eight_counts)>50]
eight_sample = sample(length(eight_mean), sample_size)
eight_lm = lm(log10(eight_sd) ~ log10(eight_mean))


mb_counts = getCPM(deng)[,deng_meta$stage=="midblast"]
mb_mean = rowMeans(mb_counts)[rowMedians(mb_counts)>50]
mb_sd = apply(mb_counts, 1, sd)[rowMedians(mb_counts)>50]
mb_sample = sample(length(mb_mean), sample_size)
mb_lm = lm(log10(mb_sd) ~ log10(mb_mean))


mouse_mean = rowMeans(getCPM(emb8))[rowMedians(getCounts(emb8))>50]
mouse_sd = apply(getCPM(emb8), 1, sd)[rowMedians(getCounts(emb8))>50]


plot_df = data.frame(mean = c(eight_mean[eight_sample], mb_mean[mb_sample], rev_sub_mean[rev_sample]),
                     sd = c(eight_sd[eight_sample], mb_sd[mb_sample], rev_sub_sd[rev_sample]),
                     data = c(rep("Deng 8-cell", sample_size), 
                              rep("Deng mid-blastocyst", sample_size), 
                              rep("Reversine embryos", sample_size)))

plot_df = plot_df[sample(nrow(plot_df), nrow(plot_df), replace = FALSE),]

mod_df = t(sapply(list(eight_lm, mb_lm, rev_lm, bl_lm), coef))
rownames(mod_df) = c("Deng 8-cell", "Deng mid-blastocyst", "Reversine embryos", "HCC38-BL")
colnames(mod_df) = c("int", "slope")


ggplot(plot_df ,aes (x = mean, y = sd, col = factor(data))) +
  geom_point(size = 0.4) +
  scale_x_log10() + scale_y_log10(breaks = c(20, 50, 10^(2:4))) +
  theme_bw() +
  scale_color_manual(values = c("Deng 8-cell" = "orchid3", "Deng mid-blastocyst" = "orchid4", "Reversine embryos" = "cyan3", "HCC38-BL" = "seagreen4"), name = "") +
  geom_abline(data = as.data.frame(mod_df), aes(slope = slope, intercept = int, col = factor(rownames(mod_df))), lwd = 1.5)
```

Figure 33: Mean-SD relationship of two populations from Deng et al  
 data. Both populations show increased variability compared to the G&T-seq 8-cell stage embryos. However, they do not show as variable expression as the HCC38-BL data.

Simulating, we will use the parameters of the largest cell population, again to avoid a mixture of developmental stages artifically increasing the variance. Results from simulations are shown in Figure 34.

**Real 8-cell stage results:**

```
kable(as.data.frame(t(mouse_test)))
```

| sensitivity | precision | fdr | specificity | accuracy | f1 | fpr |
| --- | --- | --- | --- | --- | --- | --- |
| 0.78 | 0.8863636 | 0.1136364 | 0.994877 | 0.9844055 | 0.8297872 | 0.005123 |

**Deng simulations:**

```
fraction_libsize = mean(colSums(getCounts(deng)))/mean(colSums(getCounts(emb8)))

#demonstate the matching results by plot
deng_test = get_simulated_counts(dispersion_factor = 1.3, downsample_factor = fraction_libsize, normalise = FALSE)
test_cpm = plot_test(sim_counts = deng_test, real_counts = getCounts(deng)[,getCellNames(deng)%in%deng_meta$cell[deng_meta$stage=="midblast"]])
```

Figure 34: Deng embryo simulation mean-variance plot (learnt effect size)  
 Simulated genes (coral) show similar characteristics to most genes in the true dataset.

```
#actually test the differences
deng_rep = subsample_repeat_analysis(overdisperse = 1.3,
                                     downsample = fraction_libsize,
                                     nruns = 10,
                                     grouping = emb8_meta$treatment)

kable(as.data.frame(t(apply(deng_rep, 2, mean))))
```

| sensitivity | precision | fdr | specificity | accuracy | f1 | fpr |
| --- | --- | --- | --- | --- | --- | --- |
| 0.486 | 0.7836202 | 0.2163798 | 0.9922131 | 0.9675439 | 0.5875957 | 0.0077869 |

Performance is noticeably lower than in the 8-cell stage data. However, we will continue with analysis for two reasons:

1. The degree of change is relatively low compared to the HCC38-BL and Trisomy 21 datasets considered above.
2. We do not have ground truth to compare against, and just want to compare the ASE and overall chromosomal expression levels. Our comparisons should tolerate some level of incorrect calling.

## 5.2 Comparing our detection method to ASE counts

```
deng = doAneu(deng)

#order ratios and our results the same
ratios$cell = as.character(ratios$cell)
ratios$chr = as.numeric(as.character(ratios$chr))
deng_z = getScores(deng)
deng_z$cell = as.character(deng_z$cell)
deng_z$chr = as.numeric(as.character(deng_z$chr))
deng_z = deng_z[!deng_z$cell%in%c(highcell, lowcell), ]
deng_z = deng_z[!paste(deng_z$cell, deng_z$chr) %in% c(danger1, danger2), ]
deng_z = deng_z[order(deng_z$cell, deng_z$chr), ]
ratios = ratios[order(ratios$cell, ratios$chr), ]


ratios$deviation = ratios$ratio_adj - 0.5
```

In this dataset we are calling aneuploidies at a rate of 2.59%. As we do not have proper ground-truth knowledge of aneuploidy in this case (no G&T-seq), we cannot properly assess performance.

Instead, we can relate the strength of ASE deviations from 0.5 to the Z-score our model generates. If we are effective in calling true aneuploidies, our significant calls should show greater ASE deviation.

The relationship between our method’s aneuploidy confidence calls and the ASE imbalances in chromosomes is shown in Figure 35. Z-scores to the right of the red dashed line indicate Z-score significant hits (after p-value correction), while the values above each box in the plot show the number of chromosomes considered in each Z-score bin.

```
df = data.frame(
  cell = ratios$cell,
  chr = ratios$chr,
  ratio = ratios$ratio_adj,
  dev = ratios$deviation,
  absdev = abs(ratios$deviation),
  logdev = log10(abs(ratios$deviation)),
  z = deng_z$z,
  absz = abs(deng_z$z),
  logz = log10(abs(deng_z$z))
  )
#we use the minimum z-score that remains significant for p<0.05
#to mark on the plot 'significant' values
threshold_call = min(abs(getScores(deng)$z)[getScores(deng)$p.adj < 0.05])

#-1 captures the 0s correctly, 0's from median centering
bin_limits = c(-1, 0.1, 0.5, 1, round(threshold_call, digits = 2), 5, 100)

axis_labels = sapply(2:length(bin_limits), function(x) paste0(bin_limits[x-1], "-", bin_limits[x]))
#but! we want the first and last labels to be a bit more general
axis_labels[length(axis_labels)] = paste0(">", bin_limits[length(bin_limits)-1])
axis_labels[1] = paste0("<", bin_limits[2])

#positions for numbers
xpos = 1:length(axis_labels)
#values for chr per bin
count_points = sapply(2:length(bin_limits), function(x){
  sum(df$absz > bin_limits[x-1] & df$absz < bin_limits[x])
})

#assign scores to correct bins
df$bin = sapply(df$absz, function(x) which.min(x>bin_limits)-1)
#df to plot number of chr
text_df = data.frame(pos = 1:(length(bin_limits)-1),
                     word = count_points,
                     y = rep(0.55, length(count_points)))


deng_plot = ggplot(data = df, mapping = aes(y=absdev, x = factor(bin))) +
  geom_boxplot(fill = c(rep("darkgrey", 4), rep("coral",2))) +
  geom_text(data = text_df, mapping = aes(x=pos, y = y, label = word), nudge_y = 0.00) +
  scale_x_discrete(labels = axis_labels, breaks = xpos, name = "|Aneuploidy Z-score|") +
  scale_y_continuous(name = "ASE embryo-median deviation", breaks = seq(from = 0, to = 0.5, by = 0.1)) +
  theme_bw() +
  theme(panel.border = element_blank(),
        panel.grid.major = element_blank(), 
        panel.grid.minor = element_blank(), 
        axis.line.x = element_line(colour = "black"),
        axis.line.y = element_line(colour = "black"),
        axis.title.x = element_text(face="bold", size=18, vjust=-2),
        axis.text.x  = element_text( size=12, colour = "black"),
        axis.title.y = element_text(face="bold", size=18,vjust=2),
        axis.text.y  = element_text( size=15, colour = "black"),
        plot.margin=unit(c(1,1,1.5,1.2),"cm"),
        legend.position = "none",
        plot.title = element_text(lineheight=.8, face="bold",size=30)) +
  geom_vline(xintercept = 4.5, lty = "twodash", col = "coral3")


deng_plot
```

Figure 35: ASE imbalances increase with aneuploidy prediction confidence  
 Allele specific expression biases, normalised to an embryo median, increase as chromosomes are more confidently called as aneuploid. Z-scores to the right of the red dashed line indicate Z-score significant hits after p-value correction. Numbers above boxes indicate the number of chromosomes assigned to each bin.

```
p_3a = wilcox.test(df$absdev[df$absz >= min(abs(getScores(deng)$z)[getScores(deng)$p.adj < 0.05])],
                   df$absdev[df$absz < min(abs(getScores(deng)$z)[getScores(deng)$p.adj < 0.05])])$p.value
```

The greater levels of ASE deviation seen in the high Z-score aneuploidy calls support the success of our method: the orthogonal approaches of total expression balance and allele-expression fractions are in agreement. The coral shaded bins show significantly greater ASE deviation than the grey shaded boxes (p=4.5e-10; Mann-Whiney U test)

In a similar vein, we would expect monosomies to show greater ASE deviations than trisomies: the remaining allelic ratio is 1:0 for monosomy compared to 1:2 in a trisomy. Indeed, we do also see this in our data, shown in Figure 36.

```
#add in aneuploidy state calls
df$state = "norm"

df_paste = paste(df$cell, df$chr)
hits_paste = paste(getHits(deng)$cell, getHits(deng)$chr)
for(row in 1:nrow(df)){
  #if is a hit
  if(df_paste[row] %in% hits_paste){
    # what kind of hit is it
    df$state[row] = ifelse(getHits(deng)$monosomy[which(hits_paste==df_paste[row])],
                      "mono", "tris")
  }
}

deng_plot_df = df

df$col = ifelse(df$state == "norm", "darkgrey",
                ifelse(df$state == "tris", "seagreen4", "purple"))

medians = sapply(split(df$absdev, f = df$state), mean)
median_df = data.frame(y = medians, x = match(names(medians), c("norm", "tris", "mono")))

deng_state_boxplot = ggplot(data = df, mapping = aes(y=absdev, 
                                                     x = factor(state, levels = c("norm", "tris", "mono")))) +
  geom_violin(aes(fill = factor(state)), alpha = 0.6) +
  scale_fill_manual(values = c("norm" = "darkgrey",
                               "tris" = "seagreen4",
                               "mono" = "purple")) +
  geom_point(data = median_df, mapping = aes(x=x, y= y)) +
  geom_text(data = data.frame(X=1:3, 
                              Y = rep(0.55, 3), 
                              lab = as.vector(table(df$state)[order(table(df$state), 
                                                                    decreasing = T)])),
            mapping = aes(x=X, y = Y, label = lab), nudge_y = 0.00) +
  scale_x_discrete(labels = c("Normal ploidy", "Trisomy", "Monosomy"), name = "Chromosome ploidy call") +
  scale_y_continuous(name = "ASE embryo-median deviation", breaks = seq(from = 0, to = 0.5, by = 0.1)) +
  theme_bw() +
  theme(panel.border = element_blank(),
        panel.grid.major = element_blank(), 
        panel.grid.minor = element_blank(), 
        axis.line.x = element_line(colour = "black"),
        axis.line.y = element_line(colour = "black"),
        axis.title.x = element_text(face="bold", size=18, vjust=-2),
        axis.text.x  = element_text( size=12, colour = "black"),
        axis.title.y = element_text(face="bold", size=18,vjust=2),
        axis.text.y  = element_text( size=15, colour = "black"),
        plot.margin=unit(c(1,1,1.5,1.2),"cm"),
        legend.position = "none",
        plot.title = element_text(lineheight=.8, face="bold",size=30))

deng_state_boxplot
```

Figure 36: Chromosomes called as aneuploid show characteristic ASE deviations  
 Trisomic- and monosomic-called chromosomes show increased embryo-normalised ASE deviations. Particularly, trisomic-called chromosomes show characteristic deviation at 0.16 (which corresponds to a 2:1 allele ratio), while monosomies show deviation at 0.5 (corresponding to the 1:0 allele ratio).

```
tris_norm_test = wilcox.test(df$absdev[df$state=="tris"], df$absdev[df$state=="norm"])
mono_norm_test = wilcox.test(df$absdev[df$state=="mono"], df$absdev[df$state=="norm"])
mono_tris_test = wilcox.test(df$absdev[df$state=="mono"], df$absdev[df$state=="tris"])
```

Using the Mann-Whitney test, we see that the normal ploidy calls are significantly less deviant than the trisomy (1.85e-09) and monosomy (0.00232) called chromosomes, but that the monosomy and trisomy called chromosomes are not significantly different (0.785).

If there were no cellular correction whatsoever for the aneuploidy, and genes were expressed according to their fractional presence in a cell, we would expect ASE deviations of 0.16 for trisomies (0.66 - 0.5) and of ~0.5 for monosomies. We do in fact observe this behaviour: note the density present for trisomy calls at 0.16 and for monosomy calls at 0.5 in Figure 36.

Many chromosomes do not show these characteristic deviations, however. There are additional biological effects to consider e.g. RNA turnover takes time, we may identify large CNVs in addition to whole-chromosome aneuploidies, and allelic expression is noisy (not least due to the fewer counts considered). The simulations shown in **??** also show that we anticipate that the noisiness of this data will drive some false calls.

Notably, it looks like there were no chromosomes with very severe ASE imbalances that were assigned to the normal-ploidy group.

# 6 Analysis on mESC data (F1 intercross scRNA-seq)

In thie section we replicate the findings from the Deng paper in a different dataset, using cultured mESCs. This provides an *in vitro* comparison to the previous *in vivo* section.

The counts data used for this section is available from the paper here (Kolodziejczyk et al 2015). We have separately acquired the full ASE counts from the authors.

Treatment and batch separate on the PCA shown in Figure 37, so we group analysis for our method by treatment-batch status.

```
plotPCA(mesc)
```

Figure 37: Principal components of mouse embryonic stem cell dataset  
 The first two principal components, calculated from the expression of genes used for aneuploidy calling, are shown for the cells in the mESC dataset. PC1 splits treatment, while PC2 separates some batches.

```
# plotPCA(mesc, col = substr(getGroups(mesc), 1, 3))
```

We also can check to see whether the mean-variance relationship behaves (approximately) like the successful mouse 8-cell embryos (Figure 39), as well as the library sizes (Figure 38).

```
ylims = c(0, max(density(colSums(getCounts(mesc)))$y, density(colSums(getCounts(emb8)))$y))

plot(density(colSums(getCounts(mesc))), ylim = ylims, col = "coral", main = "", xlab = "Library size")
points(density(colSums(getCounts(emb8))), type = "l", col = "cornflowerblue")
legend(x=7e6, y = 3e-7, fill = c("coral", "cornflowerblue"), legend = c("mESC", "Mouse 8-cell"))
```

Figure 38: Library sizes for mESC data  
 mESC libraries are larger than those generated in the 8-cell stage mouse embryo G&T data.

```
mesc_2i = getCPM(mesc)[, grepl("2i", getCellNames(mesc))]
mesc_2i = mesc_2i[rowMedians(mesc_2i)>50,]

mesc_a2i = getCPM(mesc)[, grepl("a2i", getCellNames(mesc))]
mesc_a2i = mesc_a2i[rowMedians(mesc_a2i)>50,]

mesc_lif = getCPM(mesc)[, grepl("lif", getCellNames(mesc))]
mesc_lif = mesc_lif[rowMedians(mesc_lif)>50,]


mean_2i = rowMeans(mesc_2i)
mean_a2i = rowMeans(mesc_a2i)
mean_lif = rowMeans(mesc_lif)

sd_2i = apply(mesc_2i, 1, sd)
sd_a2i = apply(mesc_a2i, 1, sd)
sd_lif = apply(mesc_lif, 1, sd)


lm_2i = lm(log10(sd_2i) ~ log10(mean_2i))
lm_a2i = lm(log10(sd_a2i) ~ log10(mean_a2i))
lm_lif = lm(log10(sd_lif) ~ log10(mean_lif))


sample_size = 1000
sample_rev = sample(x = length(rev_sub_mean), size = sample_size)
sample_2i = sample(x = length(mean_2i), size = sample_size)
sample_a2i = sample(x = length(mean_a2i), size = sample_size)
sample_lif = sample(x = length(mean_lif), size = sample_size)


plot_df = data.frame(mean = c(mean_2i[sample_2i], mean_a2i[sample_a2i], mean_lif[sample_lif], rev_sub_mean[sample_rev]),
                     sd = c(sd_2i[sample_2i], sd_a2i[sample_a2i], sd_lif[sample_lif], rev_sub_sd[sample_rev]),
                     data = c(rep("2i", sample_size), 
                              rep("a2i", sample_size), 
                              rep("lif", sample_size), 
                              rep("Reversine embryos", sample_size)))

plot_df = plot_df[sample(nrow(plot_df), nrow(plot_df), replace = FALSE),]

mod_df = t(sapply(list(lm_2i, lm_a2i, lm_lif, rev_lm, bl_lm), coef))
rownames(mod_df) = c("2i", "a2i", "lif", "Reversine embryos", "HCC38-BL")
colnames(mod_df) = c("int", "slope")
                     


ggplot(plot_df ,aes (x = mean, y = sd, col = factor(data))) +
  geom_point(size = 0.4) +
  scale_x_log10() + scale_y_log10(breaks = c(20, 50, 10^(2:4))) +
  theme_bw() +
  scale_color_manual(values = c("2i" = "darkslateblue", "a2i" = "cornflowerblue", "lif" = "cyan", "Reversine embryos" = "lightsalmon3", "HCC38-BL" = "chartreuse3"), name = "") +
  geom_abline(data = as.data.frame(mod_df), aes(slope = slope, intercept = int, col = factor(rownames(mod_df))), lwd = 1.5)
```

Figure 39: Mean-SD relationship of mESC data  
 All treatments show increased expression variance compared to the 8-cell stage G&T-seq data.

Variance is higher for a given mean expression than in the 8-cell embryos. We will investigate the degree to which this compromises our calling via simulations. The simulation result is shown in Figure 40.

**Real 8-cell stage results:**

```
kable(as.data.frame(t(testPerformance(emb8))))
```

| sensitivity | precision | fdr | specificity | accuracy | f1 | fpr |
| --- | --- | --- | --- | --- | --- | --- |
| 0.78 | 0.8863636 | 0.1136364 | 0.994877 | 0.9844055 | 0.8297872 | 0.005123 |

**mESC simulations:**

```
fraction_libsize = mean(colSums(getCounts(mesc)))/mean(colSums(getCounts(emb8)))

#demonstate the matching results by plot
mesc_test = get_simulated_counts(dispersion_factor = 1, downsample_factor = fraction_libsize)
test_cpm = plot_test(sim_counts = mesc_test, real_counts = getCounts(mesc))
```

Figure 40: mESC simulation mean-variance plot (learnt effect size)  
 Simulated genes (coral) show similar characteristics to most genes in the true dataset.

```
#actually test the differences
mesc_rep = subsample_repeat_analysis(overdisperse = 1,
                                     downsample = fraction_libsize,
                                     nruns = 10,
                                     grouping = emb8_meta$treatment)

kable(as.data.frame(t(apply(mesc_rep, 2, mean))))
```

| sensitivity | precision | fdr | specificity | accuracy | f1 | fpr |
| --- | --- | --- | --- | --- | --- | --- |
| 0.602 | 0.8032251 | 0.1967749 | 0.9923156 | 0.9732943 | 0.6827573 | 0.0076844 |

Performance degredation is observed, but not as severe as in the Trisomy 21 or HCC38-BL cells. Therefore it makes sense to progress as in the Deng data.

```
mesc = doAneu(mesc)
```

## 6.1 Finding ASE values

Now, in much a similar way to the Deng et al. mouse embryo data, we can look at allele specific expression to try to find signs of aneuploidy. Each cell-chromosome combination is shown in Figure 41, grouped by batch.

```
mesc_ratio_genes = getGeneTable(mesc)
# mesc_ratio_genes = mesc_ratio_genes[match(mesc_ratio_genes$gene, rownames(mesc_s129)),]

mesc_ratios = score_ase(count1 = mesc_s129, count2 = mesc_bl6, chr_map = mesc_ratio_genes)
mesc_ratios = mesc_ratios[order(mesc_ratios$cell, mesc_ratios$chr),]

mesc_ratios$index = 1:nrow(mesc_ratios)

mesc_ratios$stage = sapply(strsplit(as.character(mesc_ratios$cell), "_"), function(x) paste(x[3], x[4]))

#insert a gap between cell groupings
unq_stage = unique(mesc_ratios$stage)
stage_loc = sapply(mesc_ratios$stage, function(x) which(unq_stage == x))
adjust_multiple = stage_loc-1
shift_size = 300

mesc_ratios$new_index = mesc_ratios$index + stage_loc*shift_size 

cell_split = split(mesc_ratios, mesc_ratios$cell)

meanscores = sapply(cell_split, function(x) mean(x$ratio))


plot(x=mesc_ratios$new_index, y = mesc_ratios$ratio, xlab =  "Index", ylab = "S129 read fraction")
```

Figure 41: mESC allele read fractions  
 The S129 allele read fractions for each chromosome in each cell are plotted. Chromosomes are grouped by experimental batch. There do no appear to be any systematic biases between the batches.

We now correct for the chromosomal trends, not observing any large ASE differences between batches. The results are shown in Figures 42 and 43

```
boxplot(mesc_ratios$ratio ~ mesc_ratios$chr, ylim = c(0.3, 0.7), xlab = "CHR", ylab = "S129 read fraction")
abline(h = 0.5, lty = "twodash", col = "darkgrey")
```

Figure 42: Chromosome ASE biases in mESC data

```
mesc_ratios$ratio_adj = NA

for(chr in 1:19){
  median_score = median(mesc_ratios$ratio[mesc_ratios$chr==chr])
  mesc_ratios$ratio_adj[mesc_ratios$chr==chr] = 0.5 - median_score +
                  mesc_ratios$ratio[mesc_ratios$chr==chr]
}

boxplot(mesc_ratios$ratio_adj ~ mesc_ratios$chr, ylim = c(0.3, 0.7), xlab = "CHR", ylab = "S129 read fraction (CHR adjusted)")
abline(h = 0.5, lty = "twodash", col = "darkgrey")
```

Figure 43: ASE chromosome bias-corrected values for mESC data

Similarly to the Deng data, we do see some strongly ASE-biased chromosomes, which may well be aneuploidies.

## 6.2 Comparing our detection method to the ASE counts

Again, our high confidence aneuploidy calls are enriched for ASE-imbalanced chromosomes, shown in Figure 44

```
#sort first
mesc_z = getScores(mesc)

mesc_z$chr = as.numeric(as.character(mesc_z$chr))
mesc_z$cell = as.character(mesc_z$cell)
mesc_ratios$chr = as.numeric(as.character(mesc_ratios$chr))
mesc_ratios$cell = as.character(mesc_ratios$cell)
mesc_ratios$cell = sub(".NA", "", mesc_ratios$cell)


mesc_z = mesc_z[order(mesc_z$cell, mesc_z$chr), ]
mesc_ratios = mesc_ratios[order(mesc_ratios$cell, mesc_ratios$chr), ]

#they match now
#table(mesc_ratios$cell==mesc_z$cell & mesc_z$chr == mesc_ratios$chr)


df = data.frame(
  cell = mesc_ratios$cell,
  chr = mesc_ratios$chr,
  ratio = mesc_ratios$ratio_adj,
  dev = mesc_ratios$ratio_adj - 0.5,
  absdev = abs(mesc_ratios$ratio_adj - 0.5),
  logdev = log10(abs(mesc_ratios$ratio_adj - 0.5)),
  z = mesc_z$z,
  absz = abs(mesc_z$z),
  logz = log10(abs(mesc_z$z))
  )


threshold_call = min(abs(getScores(mesc)$z)[getScores(mesc)$p.adj < 0.05])


#-1 captures the 0s correctly
bin_limits = c(-1, 0.1, 0.5, 1, 2, round(threshold_call, 2), 5, 100)

axis_labels = sapply(2:length(bin_limits), function(x) paste0(bin_limits[x-1], "-", bin_limits[x]))
#but! we want the first and last labels to be a bit more general
axis_labels[length(axis_labels)] = paste0(">", bin_limits[length(bin_limits)-1])
axis_labels[1] = paste0("<", bin_limits[2])

xpos = 1:length(axis_labels)

count_points = sapply(2:length(bin_limits), function(x){
  sum(df$absz > bin_limits[x-1] & df$absz < bin_limits[x])
})

df$bin = sapply(df$absz, function(x) which.min(x>bin_limits)-1)

text_df = data.frame(pos = 1:(length(bin_limits)-1),
                     word = count_points,
                     y = rep(0.55, length(count_points)))


mesc_plot = ggplot(data = df, mapping = aes(y=absdev, x = factor(bin))) +
  geom_boxplot() +
  geom_text(data = text_df, mapping = aes(x=pos, y = y, label = word), nudge_y = 0.00) +
  scale_x_discrete(labels = axis_labels, breaks = xpos, name = "|Aneuploidy Z-score|") +
  scale_y_continuous(name = "ASE deviation") +
  theme_bw() +
  theme(panel.border = element_blank(),
        panel.grid.major = element_blank(), 
        panel.grid.minor = element_blank(), 
        axis.line.x = element_line(colour = "black"),
        axis.line.y = element_line(colour = "black"),
        axis.title.x = element_text(face="bold", size=18, vjust=-2),
        axis.text.x  = element_text( size=12),
        axis.title.y = element_text(face="bold", size=18,vjust=2),
        axis.text.y  = element_text( size=15),
        plot.margin=unit(c(1,1,1.5,1.2),"cm"),
        legend.position = "none",
        plot.title = element_text(lineheight=.8, face="bold",size=30)) +
  geom_vline(xintercept = 5.5, lty = "twodash", col = "coral")


mesc_plot
```

Figure 44: ASE imbalances increase with aneuploidy prediction confidence  
 Allele specific expression biases, normalised to an embryo median, increase as chromosomes are more confidently called as aneuploid. Z-scores to the right of the red dashed line indicate Z-score significant hits after p-value correction. Numbers above boxes indicate the number of chromosomes assigned to each bin.

And again, like the Deng data, we see particularly increased ASE deviation in the monosomic calls, shown in Figure 45

```
df$state = "norm"

df_paste = paste(df$cell, df$chr)
hits_paste = paste(getHits(mesc)$cell, getHits(mesc)$chr)
for(row in 1:nrow(df)){
  #if is a hit
  if(df_paste[row] %in% hits_paste){
    # what kind of hit is it
    df$state[row] = ifelse(getHits(mesc)$monosomy[which(hits_paste==df_paste[row])],
                      "mono", "tris")
  }
}

medians = sapply(split(df$absdev, f = df$state), mean)
median_df = data.frame(y = medians, x = match(names(medians), c("norm", "tris", "mono")))

mesc_state_boxplot = ggplot(data = df, mapping = aes(y=absdev, x = factor(state, levels = c("norm", "tris", "mono")))) +
  geom_violin() + #geom_boxplot
  geom_text(data = data.frame(X=1:3, 
                              Y = rep(0.55, 3), 
                              lab = as.vector(table(df$state)[order(table(df$state), 
                                                                    decreasing = T)])),
            mapping = aes(x=X, y = Y, label = lab), nudge_y = 0.00) +
  geom_point(data = median_df, mapping = aes(x=x, y= y)) +
  scale_x_discrete(labels = c("Normal ploidy", "Trisomy", "Monosomy"), name = "Chromosome ploidy call") +
  scale_y_continuous(name = "ASE deviation", breaks = seq(from = 0, to = 0.5, by = 0.1)) +
  theme_bw() +
  theme(panel.border = element_blank(),
        panel.grid.major = element_blank(), 
        panel.grid.minor = element_blank(), 
        axis.line.x = element_line(colour = "black"),
        axis.line.y = element_line(colour = "black"),
        axis.title.x = element_text(face="bold", size=18, vjust=-2),
        axis.text.x  = element_text( size=12),
        axis.title.y = element_text(face="bold", size=18,vjust=2),
        axis.text.y  = element_text( size=15),
        plot.margin=unit(c(1,1,1.5,1.2),"cm"),
        legend.position = "none",
        plot.title = element_text(lineheight=.8, face="bold",size=30))

mesc_state_boxplot
```

Figure 45: Chromosomes called as aneuploid show characteristic ASE deviations  
 Trisomic- and monosomic-called chromosomes show increased embryo-normalised ASE deviations. Particularly, trisomic-called chromosomes show characteristic deviation at 0.16 (which corresponds to a 2:1 allele ratio), while monosomies show deviation at 0.5 (corresponding to the 1:0 allele ratio). Median values are indicated by a black point.

```
batch_rate = sapply(unique(getGroups(mesc)), function(x){
  cellnames = getCellNames(mesc)[getGroups(mesc) == x]
  hits = sum(getHits(mesc)$cell %in% cellnames)
  return(hits/( length(cellnames)*19 ) ) 
  })
```

Generally, the aneuploidies called are evenly distributed over experimental batch and condition (max rate 2.39%, min rate 1.36%, mean rate 1.78%).

## 6.3 Correspondence to mESC CHR8 aneuploidies

Chromosomal instability is common in mESCs after many passages, particularly of chromosomes 8 and 11 (though the reference linked focussed on J1 mESCs rather than the G4 mESC’s used in the data we are analysing). In our mESC data, where a karyotypically normal cell was used to grow the colonies used for sequencing, we might therefore expect to see increased rates of calling of aneuploidies in chromosomes 8 and 11 as these start to build up over cell cycles.

Indeed, we do see an increased aneuploidy calling rate of chromosome 8 (representing 13.2% of all called aneuploidies), though no particularly elevated calling rate in chromosome 11, shown in Figure 46.

```
monos = as.vector(table(factor(getHits(mesc)$chr[getHits(mesc)$monosomy], levels = 1:19)))
tris = as.vector(table(factor(getHits(mesc)$chr[!getHits(mesc)$monosomy], levels = 1:19)))

count_df = data.frame(chr = rep(1:19, 2), count = c(monos, tris), type = c(rep("mono", 19), rep("tris", 19)))

ggplot(data = count_df, aes(x = chr, y = count, fill = type)) +
  geom_bar(stat = "identity") + 
  scale_fill_manual(values = c(mono = "purple", tris = "seagreen4"), labels = c("monosomy", "trisomy"), name = "Aneuploidy type") +
  scale_x_continuous(breaks = 1:19) +
  theme_bw()
```

Figure 46: Aneuploidy distribution  
 The number of called aneuploidies for each chromosome is shown. Particularly, we have identified many aneuploidies in chromosome 8, which is known to be often lose in mESC cultures.

```
tab_mesc = table(c(getHits(mesc)$chr, 1:19)) - 1
tab_deng = table(c(getHits(deng)$chr, 1:19)) - 1
```

Chromosome 7 also shows elevated rates of calling. This may be due to differing aneuploidy behaviours of different mESC lines.

Importantly, these signatures are not seen in the Deng data (the numbers of aneuploidies called for each chromosome are barely correlated between the two datasets, R=-0.2091099, Spearman correlation), suggesting that it is a feature of the mESCs and not simply of mouse genome structure (e.g. number of genes per chromosome affecting our method)

# 7 Testing data suitability for method

Here we present three features of real data that co-vary and lead to poor performance of our method. These are:

1. A residual-based score from the mean-standard deviation expression relationships of a dataset.
2. The number of genes that pass our expression threshold (median CPM > 50) in a dataset.
3. The fraction of zero counts in highly expressed genes (median CPM > 50) in a dataset.

In addition to the data analysed in the previous sections, we include five additional datasets to provide “standard”" examples for other data types:

1. One of the first UMI datasets, Zeisel et al.’s work on mouse brain samples. We consider only the oligodendrocytes to acquire a homogenous population.
2. A dataset of ~4000 peripheral blood mononuclear cells sequenced by 10X Genomics (v2 chemistry), the data for which is available on their website. We have taken the well defined cluster 2, which contains 612 cells, for the analysis here.
3. A recent UMI dataset (Tung et al.) generated from iPSCs using a Fluidigm C1 machine for library preparation. This differs from the Zeisel data by providing both transcriptomic reads alongside the UMI-collapsed molecule counts.
4. Cell libraries prepared using Cel-seq2 using a Fluidigm C1 machine (Hashimshony et al.)
5. Embryonic cell libraries prepared with Smart-Seq2 (Scialdone et al.)

## 7.1 Residual approach

We have seen above that large increases to the variance of expression data compromise the method’s performance. We therefore want a simple approach to determine whether or not our method is appropriate for a particular dataset, given its variability

We remain focussed on the the mean-standard deviation plots we have shown above, and also continue to use the G&T-seq 8 cell embryos (reversine treated) as a gold standard dataset.

Using the G&T-seq 8-cell dataset (Reversine-treated embryos only), we have fit the linear model across highly expressed (median CPM > 50) genes \(i\) (\(i \in 1,...,n\_{\text{genes}}\)):

\[ Y\_i = X\_i\beta + \epsilon \]

where \(Y\_i\) is the log10 transform of the standard deviation of gene expression for each gene, and \(X\_i\) the log10 transform of the mean gene expression for each gene (in both instances only considering genes expressed with a median count higher than 50).


Given these same quantities (\(X^\*\_j\), \(Y^\*\_j\)) from a new dataset, calculated over its highly expressed genes \(j \in 1,...,n^\*\_{\text{genes}}\) , we consider the difference to the 8-cell stage data behaviour as the sum of the “residuals” of the new data to the line fitted on the 8-cell data:

\[ r^\* = \sum\_{k=1}^{n^\*\_\text{genes}}\left(Y^\*\_k - \beta X^\*\_k \right)\]

and correct for the number of genes, which may vary between datasets

\[s^\* = \frac{r^\*}{n\_{genes}^\*}\]

this score \(s^\*\) represents the overall variability change of a dataset to the 8-cell dataset.

This process is shown schematically in Figure 47. When applied to real data, the line would correspond to the G&T-seq fit, and the points to the genes from a new dataset:

```
set.seed(42)

x_coords = runif(100,50,1000)
y_coords = sapply(x_coords, function(x) rnorm(1, mean = x * 1, sd = 200))

plot_df = data.frame(X = x_coords, Y = y_coords)


p1 = ggplot(data = plot_df, mapping = aes(x = X, y = Y)) +
    geom_segment(data = plot_df, mapping = aes(x = X, xend = X, y = X, yend = Y, col = ifelse((X-Y)<0, "one", "two"))) +
  geom_point(col = "purple") +
  geom_abline(slope = 1, intercept = 0) +
  # lims(y = c(1, 7)) +
  xlab("Gene mean expression") +
  ylab("Gene expression S.D.") +
  theme_bw()+
  theme(legend.position = "none",
        panel.grid = element_blank(),
        axis.ticks = element_blank(),
        axis.text = element_blank()) +
  ggtitle("Equivalently variable")
  

x_coords = runif(100,50,1000)
y_coords = sapply(x_coords, function(x) rnorm(1, mean = x * 1.3 + 250, sd = 200))

plot_df = data.frame(X = x_coords, Y = y_coords)


p2 = ggplot(data = plot_df, mapping = aes(x = X, y = Y)) +
    geom_segment(data = plot_df, mapping = aes(x = X, xend = X, y = X, yend = Y, col = ifelse((X-Y)<0, "one", "two"))) +
  geom_point( col = "purple" ) +
  geom_abline(slope = 1, intercept = 0) +
  # lims(y = c(1, 7)) +
  xlab("Gene mean expression") +
  ylab("Gene expression S.D.")+ 
    theme_bw()+
  theme(legend.position = "none",
        panel.grid = element_blank(),
        axis.ticks = element_blank(),
        axis.text = element_blank()) +
  ggtitle("More variable")

multiplot(p1, p2)
```

Figure 47: Gene expression variability schematics  
 In the upper plot, the genes (data points) are roughly as variable as the trend line. The sum of residuals here will be close to zero, indicating similar variability between the two datasets. In the lower plot, the genes are systematically more variable than the plotted line, so the residual sum will be high. This data is more variable than that of the plotted line.

The score in this toy data is the sum of the lengths in red, less the sum of lengths in blue, divided by the number of genes.

We use this “residual” approach because:

1. It weighs genes equally, as does our aneuploidy detection method
2. It can handle differences in the gradient and intercept of the mean-SD relationship as it operates on a gene-by-gene level

We simulate (using the method from the Section 3) and test a variety of overdispersion parameters.

```
set.seed(42)
od_factors = c(0.1, 0.3, 0.5, 0.8, 1.2, 1.6, 2, 2.5, 3, 3.5, 4, 5) 
cores = length(od_factors)

# sim_counts = lapply(od_factors, get_simulated_counts)
# names(sim_counts) = as.character(od_factors)

#generate a list of od_factors, under which are 5 simulated counts matrices
list_sim_counts = lapply(od_factors, 
                         function(x) lapply(1:10, 
                                            function(y) get_simulated_counts(dispersion_factor = x)))

#order the list_sim_counts like the emb8
list_sim_counts = lapply(list_sim_counts, function(x) lapply(x, function(y){
  y[,order(as.character(colnames(y)), decreasing = FALSE)]
}))


#make the 8cell model
gt_lm = rev_lm

ploidytests = lapply(list_sim_counts, function(x) lapply(x, function(y)
  makeAneu(counts = y, 
           genes = rownames(y), 
           chrs = getCHR(emb8), 
           cellNames = getCellNames(emb8), 
           cellGroups = getGroups(emb8))))

#To get our "residuals" at each level of the list, take the mean of the means of residuals
resid = sapply(ploidytests, 
               function(x) mean(sapply(x, 
                                       function(y) mean(getMetrics(y)$residual))))
names(resid) = as.character(od_factors)

#get performance metrics for each od factor
#add known aneuploidies
for(i in 1:length(ploidytests)){
  for(j in 1:length(ploidytests[[i]])){
    ploidytests[[i]][[j]] = setKnownAneu(ploidytests[[i]][[j]], getKnownAneu(emb8))
  }
}
#do aneuploidy calling
#for loop is slow, but it's a pain to deal with nested lists otherwise
for(i in 1:length(ploidytests)){
  for(j in 1:length(ploidytests[[i]])){
    ploidytests[[i]][[j]] = doAneu(ploidytests[[i]][[j]])
  }
}

test = lapply(ploidytests, function(pts) lapply(pts, function(x) doAneu(x)))

#get performance metrics
performance = lapply(ploidytests, function(x) t(sapply(x, function(y) testPerformance(y))))
#and find the average one
mean_performance = lapply(performance, colMeans, na.rm = T)
#store these
result_df = data.frame(od = od_factors, 
                       sens = sapply(mean_performance, function(x) x["sensitivity"]),
                       prec = sapply(mean_performance, function(x) x["precision"]),
                       fdr = sapply(mean_performance, function(x) x["fdr"])
)
```

Calculating residual scores from the simulated data, we see that the overdispersion and the residual score vary together, shown in Figure 48

```
ggplot(data = data.frame(od = as.numeric(names(resid)), 
                         resid = as.numeric(resid)), 
       mapping = aes(x = od, y = resid)) + 
  geom_point() +
  labs(x = "Overdispersion", y= "Residual score") +
  theme_bw()
```

Figure 48: Overdispersion and the calculated residual score vary together for simulations with increasing variance

As we would expect, the decreased performance of higher overdispersion (shown in Figure 49) is also manifested in the residual scores (Figure 50:

```
ggplot(data = result_df) + 
  geom_line(mapping = aes(x = od, y = sens)) + 
  geom_line(mapping = aes(x=od, y = fdr), col = "coral") +
  xlab("Overdispersion parameter") +
  ylab("") +
  annotate("text", x = 1.5, y = 0.76, label = "Sensitivity") +
  annotate("text", x = 1.5, y = 0.26, label = "FDR", col = "coral") +
  theme_bw()
```

Figure 49: Aneuploidy detection sensitivity decreases with increasing overdispersion for simulations with increasing variance

```
resid_perf_df = data.frame(residual = as.numeric(resid), sensitivity = result_df$sens, fdr = result_df$fdr)

ggplot(data = resid_perf_df) +
  geom_line(mapping = aes(x = residual, y = sensitivity)) + 
  geom_line(mapping = aes(x=residual, y = fdr), col = "coral") +
  xlab("Residual score") +
  ylab("") +
  annotate("text", x = 0.1, y = 0.7, label = "Sensitivity") +
  annotate("text", x = 0.1, y = 0.2, label = "FDR", col = "coral") +
  theme_bw()
```

Figure 50: Aneuploidy detection sentivity decreases with increasing residual score for simulations with increasing variance

Previously, we have rougly estimated a suitable overdispersion factor that simulates datasets with similar variability to real data. However, we can now directly calculate residual scores from datasets.

We now show the residual scores we have calculated from datasets overlaid on the performance of our simulations, in Figure 51.

```
data = list(gt = splitCellsByGroup(emb8)[["Control"]],
            bl = hccbl,
            tris_control = splitCellsByGroup(t21)[["Diploid"]],
            tris_tris = splitCellsByGroup(t21)[["T21"]],
            mesc = mesc,
            deng_8cell = splitCellsByGroup(deng)[["8cell"]],
            deng_lateblast = splitCellsByGroup(deng)[["lateblast"]],
            zeisel = odc,
            ten = ten,
            gilad_reads = umi_reads,
            gilad_mols = umi_mols,
            cel = cel,
            scialdone = scialdone)

data_labels = c(gt = "G&T 8 cell Control",
                bl = "HCC38-BL",
                tris_control = "Trisomy control",
                tris_tris = "Trisomy T21",
                mesc = "mESCs",
                deng_8cell = "Deng 8 cell",
                deng_lateblast = "Deng late blastocyst",
                zeisel = "Zeisel oligodendrocytes",
                ten = "10X PBMCs",
                gilad_reads = "C1 UMI reads",
                gilad_mols = "C1 UMI molecules",
                scialdone = "Scialdone embryonic cells",
                cel = "Cel-Seq2")

data_resids = sapply(data, function(x) getMetrics(x)[["residual"]])

ggplot(data = resid_perf_df) +
  geom_vline(data = data.frame(dataset = names(data_resids), residual = as.numeric(data_resids)),
             mapping = aes(xintercept = residual, col = dataset), lwd = 1.5)+
  geom_line(mapping = aes(x = residual, y = sensitivity)) + 
  geom_line(mapping = aes(x=residual, y = fdr), col = "coral") +
  xlab("Residual score") +
  ylab("") +
  annotate("text", x = 0.1, y = 0.7, label = "Sensitivity") +
  annotate("text", x = 0.1, y = 0.2, label = "FDR", col = "coral") +
  scale_colour_Publication(labels = data_labels, name = "") +
  theme_bw()
```

Figure 51: Residual scores of various datasets  
 The residual scores, calculated for data used for aneuploidy calling and other data introduced in this section, are shown. Overlaid are the aneuploidy detection sensitivity and false discovery rate for simulated data with various residual scores.

There are a few interesting things to notice in these values:

1. The poorly-performing G&T-seq data show large residual scores, as expected
2. 10X data appears to be unsuitable for this method due to its very large residual score.
3. Collapsing the C1 (Tung et al.) UMI reads into molecule counts reduces the residual score.
4. The most recent protocols and datasets (e.g. Cel-seq2 and Scialdone et al.’s Smart-Seq2 processed embryonic cells) show low residual scores, and therefore appear to be ideal for the application of our method.

As we have shown in previous sections, increased variability results in lower performance of our method. These residual scores therefore represent a simple and quick test to identify to what degree inflated gene expression deviation in a dataset will adversely affect performance.

To ensure that this residual score is relatively independent of the number of cells being considered, we have randomly downsampled the mESC data, and recalculated the residual scores. The values of ten repeated simulations for each downsampled cell number are shown in Figure 52.

```
set.seed(42)

big_resid = getMetrics(mesc)[["residual"]]
resid_50 = sapply(1:10, function(x) getMetrics(sampleCells(mesc, 50))[["residual"]])
resid_100 = sapply(1:10, function(x) getMetrics(sampleCells(mesc, 100))[["residual"]])
resid_200 = sapply(1:10, function(x) getMetrics(sampleCells(mesc, 200))[["residual"]])
resid_400 = sapply(1:10, function(x) getMetrics(sampleCells(mesc, 400))[["residual"]])


plot(x = c(rep(50, 10), rep(100, 10), rep(200, 10), rep(400, 10), ncol(getCounts(mesc))), y = c(resid_50, resid_100, resid_200, resid_400, big_resid), xlab = "Number of cells", ylab = "Residual score", ylim = c(0, 0.3))

resid_means = c(mean(resid_50), mean(resid_100), mean(resid_200), mean(resid_400), big_resid)
points(x = c(50, 100, 200, 400, ncol(getCounts(mesc))), y = resid_means, type = "l", col = "coral")
```

Figure 52: Residual score is weakly dependent on number of cells considered  
 The residual score has been calculated for mESC data, downsampled to reduced cell number. The residual score is not considerably affected by this downsampling.

## 7.2 Number of genes

Residual scores and overdispersion also affect the number of genes that can be considered under our median CPM > 50 cutoff. In Figure 53, we have plotted the number of genes in our simulations vs. their residual scores as the black line. Additionally, values obtained from real datasets have been overlaid.

```
ngenes_data = sapply(data, function(x) getMetrics(x)[["ngenes"]] )

#then through these lists, get the mean number of genes that pass the threshold
mean_genes = sapply(ploidytests, function(x) mean(sapply(x, function(y) getMetrics(y)$ngenes)))

ngene_df = data.frame(od = od_factors, ngenes = mean_genes, resid = resid)

ggplot(data = ngene_df) +
  geom_line(mapping = aes(x = resid, y = ngenes)) +
  geom_point(data = data.frame(data_resid = data_resids, data_ngenes = ngenes_data, data_name = names(data)), mapping = aes(x = data_resid, y = ngenes_data, col = data_name), size = 3) +
  scale_colour_Publication(labels = data_labels, name = "") +
  annotate("text", 0.45, 3000, label = "Overdispersion simulations") +
  labs(x = "Residual score", y = "Number of genes (median CPM > 50)") +
  theme_bw()
```

Figure 53: Number of genes that quality for aneuploidy detection varies with residual score  
 For the data considered in this manuscript, an increased level of expression variance (measured by the residual score) is observed alonside a reduction in the number of genes that are used for aneuploidy detection (median expression > 50). For simulated data, increased overdispersion also reduces the number of considered genes.

```
# names(ngenes_data) = data_labels[match(names(ngenes_data), names(data_labels))]
# print(ngenes_data)
```

The number of considered genes is an alternative metric for assessing whether our method can be applied reliably. However, it is important to note that the number of highly expressed (median CPM > 50) genes will vary by biology as well as by variability - e.g. embryonic stem cells express more genes at high levels than very specialised, mature cell types.

In terms of the observed results, it is not surprising that the number of expressed genes affects method accuracy - when we consider fewer genes, the method is less robust to noise affecting its score.

## 7.3 Fraction of zeroes

As data becomes more variable, the number of genes with 0 count will also increase (this is particularly characteristic of data with poor capture efficiency, such as droplet sequencing technologies).

We observe that the datasets that we perform worst on also show an increased number of 0 counts in their highly expressed (median CPM > 50) genes, shown in Figure 54

```
data_zeroes = lapply(data, function(x) getMetrics(x)[["zeros"]])

unlisted = unlist(data_zeroes)
split_names = strsplit(names(unlisted), split = ".", fixed = T)

zero_df = data.frame(frac = unlisted, data = sapply(split_names, function(x) x[1]))

data_labels_narrow = c(gt = "G&T 8 cell\nControl",
                       bl = "HCC38-BL",
                       tris_control = "Trisomy\ncontrol",
                       tris_tris = "Trisomy\nT21",
                       mesc = "mESCs",
                       deng_8cell = "Deng\n8 cell",
                       deng_lateblast = "Deng late\nblastocyst",
                       zeisel = "Zeisel\noligodendrocytes",
                       ten = "10X\nPBMCs",
                       gilad_reads = "C1 UMI\nreads",
                       gilad_mols = "C1 UMI\nmolecules",
                       scialdone = "Scialdone\nemb. cells",
                       cel = "Cel-Seq2")

ggplot(zero_df, mapping = aes(y = frac, x = factor(data, ordered = T, levels = names(data_zeroes)[order(sapply(data_zeroes, median))]))) +
  geom_boxplot() +
  xlab("Dataset") +
  ylab("Fraction of zeroes for genes considered") +
  scale_x_discrete(labels = data_labels_narrow) +
  theme_bw()
```

Figure 54: Fraction of zeros in genes used for aneuploidy detection  
 The most variable datasets considered in our analyses show a much higher fraction of 0 counts for genes used in aneuploidy detection.

It is likely that this plot reflects capture efficiency differences between different datasets. A low capture efficiency will lead to noisier and lower quality data.

## 7.4 Summary

We have identified three metrics that can be applied to a dataset to understand how well suited it is to our method:

1. A “residual score” to quantify how much more or less variable the expression profile is of the highly expressed (median CPM > 50) genes compared to a dataset that performs well (here, the G&T-seq 8-cell embryos)
2. The number of genes that are considered for analysis in a dataset (median CPM > 50)
3. The fraction of 0 counts that are observed for the genes that qualify for analysis (median CPM > 50)

An increase in the residual score and 0-count fraction, and a decrease in the number of genes considered all correspond to a poorer quality dataset for the purposes of this aneuploidy detection method. This has been demonstrated from real data as well as our overdispersed simulations.

We advise caution when interpreting results of our method on datasets that score poorly in these metrics.

# 8 Differential expression analyses

## 8.1 Differential expression between calls

```
#do 8cell
#there are no aneuploidies in the control data - we can't therefore
#properly control for the treatment including the control treamtent cells.
# (full rank design matrix impossible)
#therefore toss the control cells
raw_rev_counts = getCPM(splitCellsByGroup(emb8)[["Reversine"]])
raw_rev_counts = raw_rev_counts[rowMeans(raw_rev_counts)>10,]
rev_meta = emb8_meta[emb8_meta$treatment=="Reversine",]
rev_meta$aneu = rev_meta$cell%in%getHits(emb8)$cell

gt_de = get_de_genes(raw_rev_counts,
                       model.matrix(~ rev_meta$aneu))
#no de genes


#convert the deng data to ensembl ID's
name_map = getBM(mart = mouse_ensembl, values = getGenes(deng), filters = "mgi_symbol", attributes = c("ensembl_gene_id", "mgi_symbol", "chromosome_name"))
#remvoe duplicates, just keep the first ensembl version of each gene
name_map = name_map[!duplicated(name_map$mgi_symbol),]
raw_deng_translated = getCPM(deng)[getGenes(deng)%in%name_map$mgi_symbol,]
rownames(raw_deng_translated) = name_map$ensembl_gene_id[match(rownames(raw_deng_translated), name_map$mgi_symbol)]

#we only want to test genes that are expressed in both
#remove genes that aren't to help multiple testing
deng_norm = raw_deng_translated
keepgenes_deng = rownames(deng_norm)[rowMeans(deng_norm)>10]
keepgenes_mesc = rownames(getCPM(mesc))[rowMeans(getCPM(mesc))>10]
shared = keepgenes_deng[keepgenes_deng%in%keepgenes_mesc]

#regress out specific chromosome signals (e.g. so many particular trisomies don't drive signal)
mesc_chrmat = matrix(1, ncol = 19, nrow = length(getCellNames(mesc)), dimnames = list(getCellNames(mesc), 1:19))
for(hit in 1:nrow(getHits(mesc))){
  mesc_chrmat[match(as.character(getHits(mesc)$cell[hit]), rownames(mesc_chrmat)),
              match(as.character(getHits(mesc)$chr[hit]), colnames(mesc_chrmat))] = 
    ifelse(getHits(mesc)$monosomy[hit], 0.5, 1.5)
}

mesc_dmat = cbind(model.matrix(~ I(getCellNames(mesc)%in%getHits(mesc)$cell) + mesc_meta$embryo))#,mesc_chrmat)

#do mesc
mesc_de = get_de_genes(getCPM(mesc)[getGenes(mesc)%in%shared,],
                       mesc_dmat)


#regress out specific chromosome signals

deng_chrmat = matrix(1, ncol = 19, nrow = length(getCellNames(deng)), dimnames = list(getCellNames(deng), 1:19))
for(hit in 1:nrow(getHits(deng))){
  deng_chrmat[match(as.character(getHits(deng)$cell[hit]), rownames(deng_chrmat)),
              match(as.character(getHits(deng)$chr[hit]), colnames(deng_chrmat))] = 
    ifelse(getHits(deng)$monosomy[hit], 0.5, 1.5)
}

deng_dmat = cbind(model.matrix(~ I(colnames(raw_deng_translated) %in% getHits(deng)$cell) + deng_meta$stage))#,deng_chrmat)

#do Deng
raw_deng_emb_sub = raw_deng_translated
deng_meta$stage = as.character(deng_meta$stage)
deng_de = get_de_genes(raw_deng_translated[rownames(raw_deng_translated)%in%shared,],
                       deng_dmat)


#mesc/deng intersection
up_intersect = mesc_de$up[mesc_de$up %in% deng_de$up]
#length 3
down_intersect = mesc_de$up[mesc_de$down %in% deng_de$down]
#length 0

up_genes = getBM(mart = mouse_ensembl, values = up_intersect, filters = "ensembl_gene_id", attributes =  "mgi_symbol")[,1]


deng_tab = deng_de$full_tab
deng_tab$chr = name_map$chromosome_name[match(rownames(deng_tab), name_map$ensembl_gene_id)]

deng_tab$tris = sapply(deng_tab$chr, function(x) sum(getHits(deng)$chr[!getHits(deng)$monosomy] == x))
deng_tab$mono = sapply(deng_tab$chr, function(x) sum(getHits(deng)$chr[getHits(deng)$monosomy] == x))
deng_tab$diff = deng_tab$tris - deng_tab$mono


cn_plot_deng = ggplot(deng_tab, aes ( y = logFC, x = factor(diff) )) +
  geom_boxplot() +
  theme_bw() +
  labs(x = "Total CN differences", y = "log2(FC)") +
  ggtitle("Deng") +
  lims(y = c(-1, 1))


mesc_tab = mesc_de$full_tab
mesc_tab$chr = name_map$chromosome_name[match(rownames(mesc_tab), name_map$ensembl_gene_id)]

mesc_tab$tris = sapply(mesc_tab$chr, function(x) sum(getHits(mesc)$chr[!getHits(mesc)$monosomy] == x))
mesc_tab$mono = sapply(mesc_tab$chr, function(x) sum(getHits(mesc)$chr[getHits(mesc)$monosomy] == x))
mesc_tab$diff = mesc_tab$tris - mesc_tab$mono

cn_plot_mesc = ggplot(mesc_tab, aes ( y = logFC, x = factor(diff) )) +
  geom_boxplot() +
  theme_bw() +
  labs(x = "Total CN difference", y = "log2(FC)") +
  ggtitle("mESC") +
  lims (y = c(-1, 1))
```

We have performed differential expression (DE) analyses between aneuploid and normal-ploidy cells in each of the mouse datasets we have analysed (these are those with high-quality aneuploidy calls).

For the G&T-seq 8-cell embryos, we use true ploidy knowledge of each cell for our DE. For the mESCs and Deng et al. embryos we use our method’s aneuploidy calls. We have performed DE analysis using edgeR, including genes with a mean CPM expression of at least 10. For the mESC and Deng calls, we want to find a high-confidence set of DE genes; therefore we have only considered genes that are expressed with CPM>10 in *both* datasets.

We have only considered the Reversine treated embryos in the G&T-seq data, as the lack of true aneuploid cells in the Control set prevents construction of a full-rank design matrix. In the mESC data, we use as a covariate the batch-treatment status of each cell, and for the Deng. et al embryos we use the developmental stage (e.g. 16 cell, late blastocyst).

First, we need to show that the DE gene calls do not depend on the number of aneuploidies observed. For example, if we had observed many Trisomy 3 CNVs, we could be concerned that all the genes on chromosome 3 will be differentially expressed, and that we would not be capturing a cellular response to aneuploidy.

In Figure 55, we have plotted the log fold change for each gene considered for DE analysis alongside the gain or loss in number of chromosome copies across the dataset (i.e. an excess of trisomies provides a positive value).

```
multiplot(cn_plot_mesc, cn_plot_deng)
```

Figure 55: The number of observed aneuploidies does not affect DE calls  
 Plotted for the Deng and mESC datasets are the log2 fold change values, alongside the total difference in predicted copy number. A high copy number difference indicates a prevalence of trisomies in the data, and a low difference a prevalence of monosomies.

```
dm = lm(mesc_tab$logFC ~ mesc_tab$diff)
dd = lm(deng_tab$logFC ~ deng_tab$diff)
```

We considered whether the copy number difference was having any effect by regressing the log2(FC) values against the difference to complete normal ploidy (i.e. 2 copies in all cells) for each gene using ordinary least squares.

The copy number difference has no significant effect in the mESC data (p = 0.253), though is significant in the Deng data (p = 8.3e-05). However, here the coefficient value is extremely small (0.00611 compared to intercept of 0.11).

One gene is upregulated in the G&T-seq embryos: Snora68, a nucleolar RNA.

We have taken the intersection of up-regulated and down-regulated genes between the mESC and Deng datasets to find a small, high-confidence set of differentially expressed genes.

There are 22 up-regulated genes found by this intersection. These are:

Agpat4, Agrn, C1d, Canx, Capn2, Ccdc73, Gas5, Lamb1, Malat1, Mdm2, Mdm4, Pdia3, Ppp1r2, Rbm28, Riok1, Rps27l, Sdf2, Strn3, Taf1d, Txn1, Txnip, Upf2.

Of these genes, some are particularly notable:

- **Gas5** is a gene associated with growth arrest and apoptosis. Aneuploidy is known to be linked to apoptosis and cell cycle arrest.
- **Rps27l** has been reported to act with p53, where increased levels of Rps27l result in increased levels of p53. p53 promotes cell cycle arrest and apoptosis, in agreement with the increased activity of Gas5.
- **Txnip** is the mouse ortholog of **TXNIP**, a human gene which is downregulated in cancers and whose overexpression promotes G1 cell-cycle arrest
- **Calnexin (Canx)** is a protein chaperone, which operates as a part of the unfolded protein response (UPR). Activation of this response has been associated with aneuploid cells.
- **Pdia3** interacts with calnexin, and therefore is also likely to play a role in the UPR.
- **Sdf2** also acts as part of the UPR, and differential expression of SDF2 has been linked to aneuploidy incidence in human oocytes.

There were 0 shared downregulated genes.

Given that many of the differentially expressed genes play a role in aneuploidy response, this finding provides some support for our method’s success.

The G&T-seq embryos were treated with Reversine for only 8 hours before library preparation. This could have been too little time for the cells to mount an aneuploidy response, which may explain the low number of DE genes.

## 8.2 Reproduction of aneuploidy markers

A recent study of aneuploidy in human embryos acquired both genomic information and transcriptomic data (RT-qPCR) from the same cells, and trained a ploidy-state classifier on the genes they had analysed. They reported the following genes to be retained by the classifier:

BUB1, BUB3, CASP2, CDK7, CTNNB1, E2F1, GADD45A, GAPDH, PTTG1, TP53, TSC2 and YBX2.

Of these, BUB1, CASP2, GAPDH and GADD45A were more highly expressed in aneuploid cells, with the remainder more highly expressed in diploid cells

We have ground truth aneuploidy knowledge in 8-cell mouse embryos (G&T-seq), and our aneuploidy calls in the Deng et al. embryos and mESCs, alongside scRNA-seq data for each of them. We can use our calls toinvestigate the genes highlighted in the study to see if they provide a sound molecular signature for aneuploidy in our murine datasets, or in other scRNA-seq data.

First, we find the orthologous genes in the mouse for those given above.

```
aneu_up = c("BUB1", "CASP2", "GAPDH", "GADD45A")
aneu_down = c("BUB3", "CDK7", "CTNNB1", "E2F1", "PTTG1", "TP53", "TSC2", "YBX2")

#translate to ensembl ID
ensembl_ids = getBM(
  attributes = c("hgnc_symbol", "ensembl_gene_id"),
  values = c(aneu_up, aneu_down),
  filters = "hgnc_symbol",
  mart = ensembl)

orthologs = getBM(
  attributes = c("ensembl_gene_id", "mmusculus_homolog_ensembl_gene", "mmusculus_homolog_orthology_confidence"),
  values = ensembl_ids$ensembl_gene_id,
  filters = "ensembl_gene_id",
  mart = ensembl)


orthologs$hgnc = sapply(orthologs$ensembl_gene_id, function(x) ensembl_ids$hgnc_symbol[min(which(ensembl_ids$ensembl_gene_id==x))])

#GAPDH gives *loads* of entries, only one with homolog confidence
# orthologs$mmusculus_homolog_ensembl_gene[which(orthologs$hgnc=="GAPDH")] = "ENSMUSG00000057666"
orthologs = orthologs[orthologs$mmusculus_homolog_orthology_confidence==1 & !is.na(orthologs$mmusculus_homolog_orthology_confidence),]


mgis = getBM(
  attributes = c("ensembl_gene_id", "mgi_symbol"),
  values = orthologs$mmusculus_homolog_ensembl_gene,
  filters = "ensembl_gene_id",
  mart = mouse_ensembl)

orthologs$mgi = sapply(orthologs$mmusculus_homolog_ensembl_gene, 
                       function(x) mgis$mgi_symbol[mgis$ensembl_gene_id==x])

orthologs$aneu_up = orthologs$hgnc%in%aneu_up
```

We have performed differential expression analyses between the aneuploid and normal ploidy cells in the 8-cell G&T-seq embryos (where we have full confidence in ploidy from the genomic sequencing), as well as between our method-called aneuploid cells and all other cells in the Deng et al. mouse embryo data, and the mESC data that we have analysed above.

In the boxplots in Figures 56-58, we show the genes that the classifier retained for aneuploidy detection in the human embryo RT-qPCR data, with significantly differentially expressed genes (using edgeR, FDR-corrected p<0.1) highlighted with an asterisk (FDR-correction applied on these genes only). Genes on the left of the vertical line were seen to be upregulated in aneuploid cells, and genes on the right downregulated.

Note that for the embryos, we have excluded the control treatment cells as we cannot formulate a full rank matrix including treatment information, so to include these cells would confound the aneuploid status with treatment-provoked differences. We control for batch in the mESCs and stage in the Deng embryos.

```
raw_gt_reversine = getCounts(splitCellsByGroup(emb8)[["Reversine"]])
gt_special_genes = do_target_genes(raw_counts = raw_gt_reversine, 
                                     ortholog_df = orthologs, 
                                     design_matrix = model.matrix(~ I(colnames(raw_gt_reversine)
                                                                      %in%getHits(emb8)$cell)),
                                     naming_scheme = "ensembl",
                                     aneu_cells = colnames(raw_gt_reversine)[colnames(raw_gt_reversine)
                                                                         %in%getHits(emb8)$cell],
                                  title = "G&T-seq 8cell embryos"
)
```

Figure 56: Gene expression between aneuploid and normal-ploidy cells (G&T-seq data)  
 Gene selection was described above. Genes to the left of the black line have been observed in human embryos to be more lowly expressed in aneuploid cells than normal ploidy cells, while genes to the left more highly expressed. Genes marked by an asterisk show significant differential expression between aneuploid and non-aneuploid cells in this data.

```
#remove non-embryo cells
raw_deng_embryo = getCounts(deng)

deng_special_genes = do_target_genes(raw_counts = raw_deng_embryo,
                                     ortholog_df = orthologs, 
                                     design_matrix = model.matrix(~ I(getCellNames(deng)%in%
                                                                        getHits(deng)$cell) +
                                                                    as.character(deng_meta$stage)),
                                     naming_scheme = "mgi",
                                     aneu_cells = colnames(raw_deng_embryo)[getCellNames(deng)%in%
                                                                              getHits(deng)$cell],
                                     title = "Deng embryos"
)
```

Figure 57: Gene expression between aneuploid and normal-ploidy called embryonic cells (Deng data)  
 Gene selection was described above. Genes to the left of the black line have been observed in human embryos to be more lowly expressed in aneuploid cells than normal ploidy cells, while genes to the left more highly expressed. Genes marked by an asterisk show significant differential expression between aneuploid and non-aneuploid cells in this data.

```
mesc_special_genes = do_target_genes(raw_counts = getCounts(mesc), 
                                     ortholog_df = orthologs, 
                                     design_matrix = model.matrix(~ I(getCellNames(mesc) %in% getHits(mesc)$cell) +
                                                                    mesc_meta$embryo),
                                     naming_scheme = "ensembl",
                                     aneu_cells = colnames(getCounts(mesc))[colnames(getCounts(mesc)) 
                                                                     %in%getHits(mesc)$cell],
                                     title = "mESCs"
)
```

Figure 58: Gene expression between aneuploid and normal-ploidy called mES cells  
 Gene selection was described above. Genes to the left of the black line have been observed in human embryos to be more lowly expressed in aneuploid cells than normal ploidy cells, while genes to the left more highly expressed. Genes marked by an asterisk show significant differential expression between aneuploid and non-aneuploid cells in this data.

We do not consistently see significance over these genes, though we do typically see the correct changes in directionality. This may be due to a number of reasons:

1. The cellular response between murine and human cells to aneuploidy may be different. Aneuploidy is known to be common in human embryos, but not in mouse embryos. Therefore it is plausible that human development has a more effective mechanism to handle or remove these aneuploid cells.
2. The gene homologs may not function in an identical manner between the species. Therefore our differential expression comparison in this data could be less meaningful than in human data for these genes.
3. We have applied a gene-wise differential expression test compared to a classifier approach. It is not necessary for every term in a classifier to be significantly more highly or lowly expressed individually for it to provide meaningful information.
4. scRNA-seq is not as sensitive as qPCR methods - it is not as well suited to targetting specific genes in this way.

# 9 Cell cycle effects do not influence aneuploidy calling

Previous work has shown that gene dosage compensation happens very rapidly after gene duplication during S phase. However, we still want to be sure that we are not confounding our calls with cell cycle phase, where there are normal (i.e. non aneuploid) changes in copy number in the genome.

We achieve this by inferring cell cycle phase using a classifier (cyclone) and looking at how our aneuploidy calls are distributed between these phases, shown in Figure 59.

```
mouse_pairs = readRDS(system.file("exdata", "mouse_cycle_markers.rds", package="scran"))
mesc_cyclone = cyclone(x = getCPM(mesc), pairs = mouse_pairs)

mesc_meta$phase = mesc_cyclone$phases
mesc_meta$aneu = mesc_meta$cell%in%getHits(mesc)$cell

mesc_cycle_tab = table(mesc_meta$aneu, mesc_meta$phase)
mesc_frac = mesc_cycle_tab[2,] / apply(mesc_cycle_tab, 2, sum)

deng_cyclone = cyclone(x = sweep(raw_deng_translated*1e6,
                                 2, 
                                 colSums(raw_deng_translated),
                                 "/"), 
                       pairs = mouse_pairs)

deng_meta$phase = deng_cyclone$phases
deng_meta$aneu = deng_meta$cell %in% getHits(deng)$cell

deng_cycle_tab = table(deng_meta$aneu, deng_meta$phase)
deng_frac = deng_cycle_tab[2,] / apply(deng_cycle_tab, 2, sum)

cycle_df = data.frame(dataset = c(rep("mesc", 3), rep("deng", 3)), phase = c(colnames(deng_cycle_tab), colnames(deng_cycle_tab)), fraction = c(mesc_frac, deng_frac), totcells = c(colSums(mesc_cycle_tab), colSums(deng_cycle_tab)))

ggplot(data = cycle_df, mapping = aes(y = fraction, x = phase, fill = dataset)) +
  geom_bar(stat = "identity", position = "dodge") +
  labs(x = "Cell Cycle Phase", y = "Fraction aneuploid-called cells") +
  scale_fill_brewer(palette = "Accent") +
  theme_bw()
```

Figure 59: Fraction of cells containing a called aneuploid chromosome, by cyclone-inferred cell cycle phase

```
get_pairwise_fishers = function(table){
  combs = combn(1:3, 2)
  list = split(t(combs), 1:3)
  tests = sapply(list, function(x) fisher.test(table[,x])$p.value)
  return(tests)
}

ps = c(get_pairwise_fishers(mesc_cycle_tab), get_pairwise_fishers(deng_cycle_tab))

if(any(p.adjust(ps, method = "fdr")<0.05))
  print("Actually there is a significant p-value")
```

No pairwise Fisher’s tests show significant (p<0.05, after FDR-correction) differences between calling rates by cell cycle phases in each dataset. From this, we conclude that cell-cycle stage is not confounding.

# 10 Manuscript + Supplementary graphics

The following section documents generation of the plots used in the manuscript that this report supplements. It therefore shows clearly how we have created the figures that have been used in the manuscript, and therefore is probably only of interest if you would like to review specific pieces of code.

```
set.seed(5)
scores = c(rnorm(20, mean = 1, sd = 0.07), rnorm(2, mean = 1.4, sd = 0.05), rnorm(2, mean = 0.5, sd = 0.05))
hist_df = data.frame(X=scores, col = c(rep("black", 20), rep("seagreen4", 2), rep("purple", 2)))
alt_1a = ggplot(data = hist_df, mapping = aes(x = X, col = col, fill = col)) + 
  geom_histogram() +
  xlab ("Chromosome score") +
  ylab("Frequency") +
  scale_x_continuous(labels = c(0.5, 1, 1.5), breaks = c(0.5, 1, 1.5)) + 
  theme_bw() + 
  theme(panel.grid = element_blank(), 
        legend.position = "none", 
        axis.ticks.y = element_blank(), 
        axis.text.y = element_blank(), 
        panel.border = element_blank(),
        axis.line.x = element_line(colour = "black"),
        axis.line.y = element_line(colour = "black"), 
        axis.title.x = element_text(face="bold", size=20, vjust=-2),
        axis.text.x  = element_text( size=15, face = "bold"),
        axis.title.y = element_text(face="bold", size=20,vjust=2)) +
  coord_cartesian(xlim = c(0.45, 1.55), ylim = c(0, 8), expand = F) +
  scale_color_manual(values = c("grey50", "purple", "seagreen4")) +
  scale_fill_manual(values = c("grey50", "purple", "seagreen4"))


alt_1a
```

```
pdf(useDingbats = F, file = paste0(plot_folder, "/1a_schematic.pdf"), width = 5, height = 5)
alt_1a
dev.off()
```

```
## png 
##   2
```

```
zs = getScores(emb8)

monos = getKnownAneu(emb8)[getKnownAneu(emb8)$monosomy,]
tris = getKnownAneu(emb8)[!getKnownAneu(emb8)$monosomy,]

zs$truth = "none"
zs$truth[paste(zs$cell, zs$chr) %in% paste(monos$cell, monos$chr)] = "loss"
zs$truth[paste(zs$cell, zs$chr) %in% paste(tris$cell, tris$chr)] = "gain"

id.mono<-which(zs$truth=="loss")
id.tri<-which(zs$truth=="gain")

our_monos = getHits(emb8)[getHits(emb8)$monosomy, ]
our_tris = getHits(emb8)[!getHits(emb8)$monosomy, ]
zs$us = "none"
zs$us[paste(zs$cell, zs$chr) %in% paste(our_monos$cell, our_monos$chr)] = "loss"
zs$us[paste(zs$cell, zs$chr) %in% paste(our_tris$cell, our_tris$chr)] = "gain"

zs$embryo = substr(zs$cell, 1, 1)

#figure ######
set.seed(5)

x_coord = jitter(as.numeric(as.factor(zs$embryo)), factor = 1.2)

df<-data.frame(x=x_coord,#x-coordinate (random, centred around the embryo number) 
               y=zs$z, #z-score
               #y=zs$ratio, #aneuploidy score
               truth= zs$truth, #aneuploidy as in the G&T paper
               hits=zs$us) #aneuploidy as in our analysis

p<-ggplot(df, aes(x=x, y=y))+
  #geom_hline(yintercept=1, lty=2)+
   geom_hline(yintercept=0, lty=2)+
   geom_point(size=.6)+
   geom_point(data=df[df$truth=="loss",], aes(x=x, y=y), col="purple", shape=0, size=4,stroke=1.0)+#shape=1
   geom_point(data=df[df$truth=="gain",], aes(x=x, y=y), col="seagreen4", shape=0, size=4,stroke=1.0)+#shape=1
   geom_point(data=df[df$hits=="loss",], aes(x=x, y=y), col="black", shape=16, size=3)+#shape=18
   geom_point(data=df[df$hits=="loss",], aes(x=x, y=y), col="purple", shape=16, size=2)+
   geom_point(data=df[df$hits=="gain",], aes(x=x, y=y), col="black", shape=16, size=3)+#shape=18
   geom_point(data=df[df$hits=="gain",], aes(x=x, y=y), col="seagreen4", shape=16, size=2)+
  #  ylim(0.38,1.62)+
   xlab("Embryo")+
   ylab("Z-score")+
   theme_bw() + 
   theme(panel.border = element_blank(), 
         panel.grid.major = element_blank(), 
         panel.grid.minor = element_blank(), 
         axis.line.y = element_line(colour = "black"),
         axis.line.x = element_line(colour = "black"),
         axis.title.x = element_text(face="bold", size=20, vjust=-2),
        axis.text.x  = element_text( size=15),
        axis.title.y = element_text(face="bold", size=20,vjust=2),
        axis.text.y  = element_text( size=15),
        plot.margin=unit(c(1,1,1.5,1.2),"cm"),
        legend.text=element_text(size=25),#size of legend
        legend.title=element_text(size=25),
        plot.title = element_text(lineheight=.8, face="bold",size=30))+
  scale_x_discrete("Embryo",
                   expand = c(0.1,0.1),
                   labels=c("A","B","C","D","E","F","G"),
                   limits=c(1,2,3,4,5,6,7))

p
```

```
pdf(useDingbats = F, file = paste0(plot_folder, "/1b_8cell.pdf"), width = 12, height = 7)
p
dev.off()
```

```
## png 
##   2
```

```
print("Mouse 8 cell")
```

```
## [1] "Mouse 8 cell"
```

```
print(mouse_test)
```

```
## sensitivity   precision         fdr specificity    accuracy          f1 
## 0.780000000 0.886363636 0.113636364 0.994877049 0.984405458 0.829787234 
##         fpr 
## 0.005122951
```

```
print("HCC38-BL")
```

```
## [1] "HCC38-BL"
```

```
hccbl_test = testPerformance(hccbl)
print(hccbl_test)
```

```
## sensitivity   precision         fdr specificity    accuracy          f1 
##  0.28571429  0.15384615  0.84615385  0.98707403  0.98135198  0.20000000 
##         fpr 
##  0.01292597
```

```
print("Tris21")
```

```
## [1] "Tris21"
```

```
print(testPerformance(t21_sub))
```

```
## sensitivity   precision         fdr specificity    accuracy          f1 
## 0.000000000 0.000000000 1.000000000 0.993333333 0.967532468 0.000000000 
##         fpr 
## 0.006666667
```

```
set.seed(42)

palette = c("BL" = "#444C5C", "Reversine" = "#E1B16A", "T21" = "#78A5A3")
bl_col = palette[1]
bl_line = bl_col
rev_col = palette[2]
rev_line = rev_col
t21_col = palette[3]
t21_line = t21_col

npoints = 200
bl_rows = sample(length(bl_sub_mean), npoints)
rev_rows = sample(length(rev_sub_mean), npoints)
t21_rows = sample(length(norm_mean), npoints)


df1 = data.frame(mean = bl_sub_mean[bl_rows], sd = bl_sub_sd[bl_rows], line = "BL")
df2 = data.frame(mean = rev_sub_mean[rev_rows], sd = rev_sub_sd[rev_rows], line = "Reversine")
df3 = data.frame(mean = norm_mean[t21_rows], sd = norm_sd[t21_rows], line = "T21")
df_fin = rbind(df1, df2, df3)
# scramble plot order
df_fin = df_fin[sample(nrow(df_fin), nrow(df_fin), replace = FALSE), ]

p = ggplot(data = df_fin, mapping = aes(x = mean, y=sd, col = line)) +
  geom_point(alpha = 1) + 
  scale_x_log10(breaks = c(50, 250, 1000, 5000, 25000), limits = c(40, 1000)) + 
  scale_y_log10(breaks = c(10, 25, 50, 250, 1000, 5000), limits = c(10, 2000)) +
  labs(x = "Gene expression mean", y = "Gene expression \nstandard deviation") +
  geom_abline(slope = bl_lm$coefficients[2], intercept = bl_lm$coefficients[1], col = bl_line, lwd = 1.5) +
  geom_abline(slope = rev_lm$coefficients[2], intercept = rev_lm$coefficients[1], col = rev_line, lwd = 1.5) +
  geom_abline(slope = norm_lm$coefficients[2], intercept = norm_lm$coefficients[1], col = t21_line, lwd = 1.5) +
  scale_color_manual(values = palette, name = "Dataset", labels = c("HCC38-BL cell line", "Reversine-treated 8-cell embryos", "Trisomy 21 iPS derived neurons")) +
  theme_bw() +
  theme(panel.border = element_blank(), 
        panel.grid.major = element_blank(), 
        panel.grid.minor = element_blank(), 
        axis.line.x = element_line(colour = "black"),
        axis.line.y = element_line(colour = "black"),
        axis.title.x = element_text(face="bold", size=18, vjust=-2),
        axis.text.x  = element_text( size=15, colour = "black"),
        axis.title.y = element_text(face="bold", size=18,vjust=2),
        axis.text.y  = element_text( size=15, colour = "black"),
        plot.margin=unit(c(1,1,1.5,1.2),"cm"),
        # legend.position = "none",
        plot.title = element_text(lineheight=.8, face="bold",size=30)) +
  guides(colour = guide_legend(override.aes = list(alpha = 1)))


p
```

```
pdf(useDingbats = F, file = paste0(plot_folder, "/2b_dispersion.pdf"), width = 8, height = 5)
p
dev.off()
```

```
## png 
##   2
```

```
set.seed(42)

exp_raw_gt_8cell = getCounts(emb8)[rowMeans(getCounts(emb8))>0,]
raw_aneu = getCounts(emb8)[, colnames(exp_raw_gt_8cell)%in%getKnownAneu(emb8)$cell]
raw_fine = getCounts(emb8)[, !colnames(exp_raw_gt_8cell)%in%getKnownAneu(emb8)$cell]
raw_38 = getCounts(hcc38)
raw_bl = getCounts(hccbl)


#decide the overdispersion required
# od_factors = seq(from = 0.25, to = 2.25, by = 1)
od_factors = c(0.3, 0.8, 2, 5) 
#do the simulation, name, and subset to CPM>50
sim_out = lapply(od_factors, get_simulated_counts)
names(sim_out) = as.character(od_factors)
highexp = lapply(sim_out, function(x) x[rowMedians(x)>50, ])
#get means and variances
means = lapply(highexp, rowMeans)
vars = lapply(highexp, function(x) apply(x, 1, sd))


#downsample for plotting
n_genes = min(sapply(means, length))
sample_points = sample(n_genes, 500)
means_sub = lapply(means, function(x) x[sample_points])
vars_sub = lapply(vars, function(x) x[sample_points])


#melt into a single df
var_df = melt(vars_sub)
mean_df = melt(means_sub)
main_df = var_df
main_df$mean = mean_df$value
names(main_df) = c("sd", "od", "mean")

main_df = main_df[sample(nrow(main_df), nrow(main_df), replace = FALSE),]

sim_palette = rev(brewer.pal(4, "RdYlBu"))

#gradient data
grads = as.data.frame(rbind(coef(rev_lm), coef(bl_lm)))
names(grads) = c("intercept", "slope")
grads$cols = c("rev", "bl")

p = ggplot(data = main_df, mapping = aes(x=mean, y = sd, col = od)) +
  geom_point(alpha = 1) +
  scale_x_log10(breaks = c(50, 250, 1000, 5000), limits = c(40,1000)) + 
  scale_y_log10(breaks = c(50, 250, 1000, 5000), limits = c(20,2000)) +
  #geom_smooth(method = "lm", se = FALSE, fullrange = TRUE, size = 0.5, lty = "twodash") +
  # geom_abline(data = grads, mapping = aes(slope = slope, intercept = intercept, col = cols ), lwd = 1) +
  geom_abline(slope = coef(rev_lm)[2], intercept = coef(rev_lm)[1], color = rev_line, lwd = 1.3) +
  geom_abline(slope = coef(bl_lm)[2], intercept = coef(bl_lm)[1], color = bl_line, lwd = 1.3) +
  scale_color_manual(values = sim_palette, breaks = od_factors, name = "Simulation dispersion parameter") +
  # scale_linetype_manual(name = "Fitted trend from data", values = c("Reversine mouse embryos" = rev_col, "HCC38-BL" = bl_col),  guide="legend") +
  labs(x = "Gene expression mean", y = "Gene expression \nstandard deviation") +
  theme_bw()+
  theme(panel.border = element_blank(), 
        panel.grid.major = element_blank(), 
        panel.grid.minor = element_blank(), 
        axis.line.x = element_line(colour = "black"),
        axis.line.y = element_line(colour = "black"),
        axis.title.x = element_text(face="bold", size=18, vjust=-2),
        axis.text.x  = element_text( size=15, colour = "black"),
        axis.title.y = element_text(face="bold", size=18,vjust=2),
        axis.text.y  = element_text( size=15, colour = "black"),
        plot.margin=unit(c(1,1,1.5,1.2),"cm"),
        plot.title = element_text(lineheight=.8, face="bold",size=30)) +
  guides(colour = guide_legend(override.aes = list(alpha = 1)))

  
  
p
```

```
pdf(useDingbats = F, file = paste0(plot_folder, "/2c_simulations.pdf"), width = 8, height = 5)
p
dev.off()
```

```
## png 
##   2
```

```
extra_legend = ggplot(data = data.frame(X=1:2, Y=1:2, cols = 1:2), mapping = aes(x=X, y=Y,col = factor(cols))) +
  theme_bw() +
  geom_abline(mapping = aes(slope=X, intercept = Y, col = factor(cols))) +
  scale_color_manual(values = c(rev_line, bl_line, use.names = FALSE), labels = c("Reversine-treated 8-cell embryos", "HCC38-BL cell line"), name = "Trend fitted from data") +
  guides(colour = guide_legend(override.aes = list(size=1)))

pdf(useDingbats = F, file = paste0(plot_folder, "/2c_sim_extralegendonly.pdf"), width = 6, height = 5)
extra_legend
dev.off()
```

```
## png 
##   2
```

```
set.seed(42)

#od_factors sourced from above, we'll sort
sim_out = lapply(od_factors, function(x) subsample_repeat_analysis(overdisperse = x, nruns = 10))
#remove NaN's

means = lapply(sim_out, colMeans, na.rm = T)

result_df = data.frame(od = od_factors, 
                       sens = sapply(means, function(x) x["sensitivity"]),
                       prec = sapply(means, function(x) x["precision"]),
                       fdr = sapply(means, function(x) x["fdr"])
)

df.m = melt(result_df, id.vars = 1)

p = ggplot(data = df.m, mapping = aes(x=factor(variable), y=value)) +
  geom_bar(aes(fill = factor(od)), position = "dodge", stat = "identity", alpha = 0.8) +
  scale_fill_manual(values = sim_palette, name = "Simulation dispersion parameter") +
  theme_bw() +
  theme(
        axis.title.y = element_blank(),
        panel.grid = element_blank(),
        panel.border = element_blank(), 
        axis.line = element_line(),
        axis.title.x = element_text(face="bold", size=18, vjust=-2),
        axis.text.x  = element_text( face = "bold", size=15, colour = "black"),
        axis.text.y  = element_text( size=15, colour = "black"),
        axis.ticks.x = element_blank()) +
  scale_x_discrete("",
                   expand = c(0.1,0.1),
                   labels=c("Sensitivity", "Precision", "FDR")) +
  scale_y_continuous(limits = c(0,1), breaks = seq(from = 0, to = 1, by = 0.25)) +
  coord_cartesian(ylim = c(0,1), xlim = c(0.4, 3.6), expand = F)
  

p
```

```
pdf(useDingbats = F, file = paste0(plot_folder, "/2d_sim_result.pdf"), width = 9, height = 5)
p
dev.off()
```

```
## png 
##   2
```

```
#See the Deng "plot" code chunk for the code here

deng_plot
```

```
pdf(useDingbats = F, file = paste0(plot_folder, "/3a_deng_score_comparison.pdf"), width = 9, height = 7)
deng_plot
dev.off()
```

```
## png 
##   2
```

```
print("wilcox test p value")
```

```
## [1] "wilcox test p value"
```

```
print(p_3a)
```

```
## [1] 4.460402e-10
```

```
deng_state_boxplot
```

```
pdf(useDingbats = F, file = paste0(plot_folder, "/3b_deng_call_ase.pdf"), width = 7, height = 7)
deng_state_boxplot
dev.off()
```

```
## png 
##   2
```

```
#alternative
alt_3b = ggplot(data = deng_plot_df, mapping = aes(x=absdev)) +
   geom_histogram(mapping = aes(y=..density..)) +
   geom_density() +
   facet_grid(state~.)

ggsave(alt_3b, file = paste0(plot_folder, "/alt_3b.pdf"))

print("Tris call vs Norm call ASE deviation p:")
```

```
## [1] "Tris call vs Norm call ASE deviation p:"
```

```
tris_norm_test$p.value
```

```
## [1] 1.848645e-09
```

```
print("Mono call vs Norm call ASE deviation p:")
```

```
## [1] "Mono call vs Norm call ASE deviation p:"
```

```
mono_norm_test$p.value
```

```
## [1] 0.002318213
```

```
print("Tris call vs Mono call ASE deviation p:")
```

```
## [1] "Tris call vs Mono call ASE deviation p:"
```

```
mono_tris_test$p.value
```

```
## [1] 0.7848091
```

```
mesc_plot
```

```
pdf(useDingbats = F, file = paste0(plot_folder, "/S1_mesc_score_comparison.pdf"), width = 9, height = 7)
mesc_plot
dev.off()
```

```
## png 
##   2
```

```
mesc_state_boxplot
```

```
pdf(useDingbats = F, file = paste0(plot_folder, "/S2_mesc_call_ase.pdf"), width = 7, height = 7)
mesc_state_boxplot
dev.off()
```

```
## png 
##   2
```

# 11 R Session Information

```
sessionInfo()
```

```
## R version 3.4.1 (2017-06-30)
## Platform: x86_64-pc-linux-gnu (64-bit)
## Running under: Red Hat Enterprise Linux Server 7.3 (Maipo)
## 
## Matrix products: default
## BLAS: /nfs/research2/marioni/jonny/software/R-3.4.1/bin/lib64/R/lib/libRblas.so
## LAPACK: /nfs/research2/marioni/jonny/software/R-3.4.1/bin/lib64/R/lib/libRlapack.so
## 
## locale:
## [1] C
## 
## attached base packages:
##  [1] grid      parallel  stats4    methods   stats     graphics  grDevices
##  [8] utils     datasets  base     
## 
## other attached packages:
##  [1] scales_0.5.0                ggplot2_2.2.1              
##  [3] scploid_0.9                 knitr_1.17                 
##  [5] Matrix_1.2-11               RColorBrewer_1.1-2         
##  [7] edgeR_3.19.3                limma_3.33.9               
##  [9] reshape2_1.4.2              biomaRt_2.33.4             
## [11] scran_1.5.9                 SingleCellExperiment_0.99.3
## [13] SummarizedExperiment_1.7.6  DelayedArray_0.3.19        
## [15] matrixStats_0.52.2          Biobase_2.37.2             
## [17] GenomicRanges_1.29.13       GenomeInfoDb_1.13.4        
## [19] IRanges_2.11.15             S4Vectors_0.15.7           
## [21] BiocGenerics_0.23.1         BiocParallel_1.11.8        
## [23] here_0.1                    BiocStyle_2.5.37           
## [25] rmarkdown_1.6              
## 
## loaded via a namespace (and not attached):
##  [1] bitops_1.0-6            bit64_0.9-7             progress_1.1.2         
##  [4] rprojroot_1.2           dynamicTreeCut_1.63-1   tools_3.4.1            
##  [7] backports_1.1.0         DT_0.2                  R6_2.2.2               
## [10] vipor_0.4.5             DBI_0.7                 lazyeval_0.2.0         
## [13] colorspace_1.3-2        gridExtra_2.3           prettyunits_1.0.2      
## [16] bit_1.1-12              compiler_3.4.1          labeling_0.3           
## [19] bookdown_0.5            stringr_1.2.0           digest_0.6.12          
## [22] XVector_0.17.1          scater_1.5.12           pkgconfig_2.0.1        
## [25] htmltools_0.3.6         highr_0.6               htmlwidgets_0.9        
## [28] rlang_0.1.2             RSQLite_2.0             FNN_1.1                
## [31] shiny_1.0.5             bindr_0.1               zoo_1.8-0              
## [34] dplyr_0.7.3             RCurl_1.95-4.8          magrittr_1.5           
## [37] GenomeInfoDbData_0.99.1 Rcpp_0.12.12            ggbeeswarm_0.6.0       
## [40] munsell_0.4.3           viridis_0.4.0           stringi_1.1.5          
## [43] yaml_2.1.14             zlibbioc_1.23.0         rhdf5_2.21.4           
## [46] plyr_1.8.4              blob_1.1.0              shinydashboard_0.6.1   
## [49] lattice_0.20-35         splines_3.4.1           locfit_1.5-9.1         
## [52] igraph_1.1.2            rjson_0.2.15            XML_3.98-1.9           
## [55] glue_1.1.1              evaluate_0.10.1         data.table_1.10.4      
## [58] httpuv_1.3.5            gtable_0.2.0            assertthat_0.2.0       
## [61] mime_0.5                xtable_1.8-2            viridisLite_0.2.0      
## [64] tibble_1.3.4            AnnotationDbi_1.39.3    beeswarm_0.2.3         
## [67] memoise_1.1.0           tximport_1.5.0          bindrcpp_0.2           
## [70] statmod_1.4.30
```
