# Supplementary material for: Mosaic autosomal aneuploidies are detectable from single-cell RNAseq data
Source: BMC Genomics. 2017 Nov 25;18:904. doi: 10.1186/s12864-017-4253-x (PMC5702132; doi:10.1186/s12864-017-4253-x)
Supplement: Supplementary file 1 — Code for analysis. A gzipped tarball containing all code used for analysis, as well as the.html report referred to above. A package to run aneuploidy assessment in R is also included, alongside a script to download the data we have used. The latest version of these files may be found on https://github.com/MarioniLab/Aneuploidy2017. (GZ 6405 kb) [file 12864_2017_4253_MOESM1_ESM.gz › Aneuploidy2017/scploid_usage.html]

scploid Package Usage Guide


Code 

- Show All Code
- Hide All Code

# scploid Package Usage Guide

Jonathan Griffiths

#### *23 September, 2017*

This document describes the usage of the `scploid` package to call aneuploidies from single-cell data.

We will load some useful packages first:

```
library(scploid)

#here() allows us to point to files in a robust way
library(here)
fold = here()

#biomaRt allows us to get the gene locations
library(biomaRt)

#knitr tidies up some output for this document
library(knitr)

#BiocStyle is a pretty R markdown output
library(BiocStyle)
```

To install the package, you can run:

`library(devtools)` `devtools::install_github("MarioniLab/Aneuploidy2017", subdir = "package")`

First, we load in the data - we use the G&T-seq 8-cell stage embryos that we considered in the paper:

```
counts = read.table(
  paste0(fold, "/raw_data/gt_8cell_counts.txt"),
  header = T,
  row.names = 1
)

#tidy up the cell names
colnames(counts) = paste0("cell_", 1:ncol(counts))
```

The count table is formatted with cells as the columns, and genes as the rows, shown in Table 1:

Table 1: The first five rows and columns of the counts matrix

|  | cell\_1 | cell\_2 | cell\_3 | cell\_4 | cell\_5 |
| --- | --- | --- | --- | --- | --- |
| ENSMUSG00000000001 | 10 | 219 | 169 | 48 | 272 |
| ENSMUSG00000000003 | 0 | 0 | 0 | 0 | 0 |
| ENSMUSG00000000028 | 44 | 168 | 797 | 459 | 470 |
| ENSMUSG00000000031 | 0 | 0 | 0 | 0 | 0 |
| ENSMUSG00000000037 | 0 | 33 | 0 | 2 | 0 |

We now need to identify the chromosome that each gene lies on. We do this with biomaRt.

```
mouse_ensembl = useMart("ensembl")
mouse_ensembl = useDataset("mmusculus_gene_ensembl", 
                           mart = mouse_ensembl)

gene_table = getBM(
  attributes = c("ensembl_gene_id", "chromosome_name"),
  mart = mouse_ensembl,
  values = as.character(rownames(counts)),
  filters = "ensembl_gene_id"
)
```

We want to assess autosomal chromosomes, so we retain only genes on these.

```
gene_table = gene_table[gene_table$chromosome_name %in% 1:19, ]
counts = counts[gene_table$ensembl_gene_id, ]
```

Finally, we can make the `ploidytest` object, which holds data and performs aneuploidy calculations for us. We need:

- The counts table (integer matrix)
- The gene names (chracter vector)
- The gene chromosomes (character or numeric vector)
- Cell names (character vector)
- Cell groups for analysis to avoid DE-driven effects (character vector)

We make one such object below:

```
ploidytest = makeAneu(counts = as.matrix(counts), 
                      genes = gene_table$ensembl_gene_id, 
                      chrs = gene_table$chromosome_name, 
                      cellNames = colnames(counts), 
                      cellGroups = rep("group_1", ncol(counts)))
```

The method only considers genes with median expression above 50 CPM. We can check to see if there is any structure in these genes that we might have missed, by plotting the first two components of a PCA. The PCA is performed on centred log-transformed CPM. You can discretely colour this plot any way you like, in case there are any covariates you would like to check for confounding. We show this using random colours in Figure 1.

```
random_colours = round(runif(ncol(counts), 0.5, 4.5))

plotPCA(ploidytest, cols = random_colours)
```

Figure 1: The first two principal components of CPM counts, coloured randomly

If there is any covariate-driven structure, make the ploidytest object again and change the cell groupings. Here, we can see two cells that behave very differently to the others: these were QC-fail cells.

You may now want to change some of the parameters of the aneuploidy calling approach. These are:

- `p.thresh`: post FDR correction p-value for aneuploidy calling (default 0.1)
- `min.deviation`: minimum deviation of \(s\_{ij}\) from 1 for aneuploidy calling (default 0.2, i.e. \(0.8<s\_{ij}<1.2\) will never be considered as aneuploid)
- `center.cells`: logical value to allow or prevent within-cell \(s\_{ij}\) centering (default TRUE)
- `min.median`: minimum CPM median value for gene retention for analysis (default 50)
- `extreme.gene.thresh`: maximum \(a\_{gij}\) that one gene may show before removal as outlier (default 10, to exclude no genes set to a very high value e.g. `Inf`)

You can check a parameter using `getParam`, like so:

```
getParam(ploidytest, 
         param_name = "p.thresh")
```

```
## [1] 0.1
```

And change them using `setParam`:

```
ploidytest = setParam(ploidytest, 
                      param_name = "center.cells", 
                      param_value = FALSE)
```

The `extreme.gene.thresh` parameter is designed to exclude genes that show a large amount of differential expression within cell groupings. This differential expression could contribute artefactually high scores to aneuploidy calls. You can review the scores of genes in groups using the `getMaxA()` function:

```
max_a = getMaxA(ploidytest)
head(max_a)
```

```
## ENSMUSG00000097971 ENSMUSG00000021714 ENSMUSG00000042029 ENSMUSG00000040693 
##          12.773004           9.587493           8.650824           8.401957 
## ENSMUSG00000028820 ENSMUSG00000020458 
##           8.337805           8.331815
```

If you would like to exclude genes like this, you can set the `extreme.gene.thresh` parameter. The `print` argument can provide feedback to you about how many genes are being removed. If print is TRUE, the number of genes on each chromosome that will be excluded by the extreme gene cutoff is printed, separately for each cell group defined:

```
ploidytest = setParam(ploidytest, 
                      param_name = "extreme.gene.thresh", 
                      param_value = 8,
                      print = TRUE)
```

```
##                                 1   2   3   4   5   6   7   8   9  10  11
## group_1 genes considered      195 296 158 240 221 156 227 199 205 167 324
## group_1 extreme genes dropped   1   0   0   2   0   0   0   0   0   0   1
##                                12  13  14  15  16  17 18  19
## group_1 genes considered      125 124 113 131 119 168 85 117
## group_1 extreme genes dropped   1   1   0   0   0   1  0   0
```

This may help you prevent the exclusion of too many genes by setting the cutoff too low.

Once you are happy with your setup, you can run the aneuploidy calculations.

```
ploidytest = doAneu(ploidytest)
```

You can look at the scores for each chromosome using `getScores`, which presents all chromosomes tested. Columns names are:

- `chr`: Chromosome tested
- `cell`: Cell tested
- `z`: Z-score calculated from \(s\_{ij}\)
- `score`: Value of \(s\_{ij}\)
- `p`: Raw p-value from Z-score
- `p.adj`: FDR-adjusted p-value
- `monosomy`: If expression is depressed compared to other cells, this is TRUE, otherwise FALSE. It does not indicate a called aneuploidy for this function.

The head of the scores table is shown in Table 2.

Table 2: Scores of aneuploidy assessment

|  | chr | cell | z | score | p | p.adj | monosomy |
| --- | --- | --- | --- | --- | --- | --- | --- |
| group\_1.1 | 1 | cell\_1 | -1.205581 | 0.9694649 | 0.2352430 | 0.8655804 | TRUE |
| group\_1.2 | 10 | cell\_1 | 5.535568 | 1.4425614 | 0.0000023 | 0.0001737 | FALSE |
| group\_1.3 | 11 | cell\_1 | 3.721813 | 1.2410683 | 0.0006229 | 0.0157806 | FALSE |
| group\_1.4 | 12 | cell\_1 | -2.447870 | 0.8726135 | 0.0189740 | 0.2288929 | TRUE |
| group\_1.5 | 13 | cell\_1 | -1.890310 | 0.8744468 | 0.0661642 | 0.5079481 | TRUE |
| group\_1.6 | 14 | cell\_1 | -1.233728 | 1.0079661 | 0.2246880 | 0.8655804 | TRUE |

To look at just the called aneuploidies, use `getHits`. Here, only the chromosomes that were p-value and \(s\_{ij}\)-deviant are returned. The head of the “hits” table is shown in Table 3.

Table 3: Scores of called aneuploid chromosomes

|  | chr | cell | z | score | p | p.adj | monosomy |
| --- | --- | --- | --- | --- | --- | --- | --- |
| group\_1.2 | 10 | cell\_1 | 5.535568 | 1.4425614 | 0.0000023 | 0.0001737 | FALSE |
| group\_1.3 | 11 | cell\_1 | 3.721813 | 1.2410683 | 0.0006229 | 0.0157806 | FALSE |
| group\_1.15 | 5 | cell\_1 | 11.631552 | 1.6570165 | 0.0000000 | 0.0000000 | FALSE |
| group\_1.53 | 5 | cell\_3 | 3.277819 | 1.2301227 | 0.0022039 | 0.0398800 | FALSE |
| group\_1.65 | 16 | cell\_4 | -3.575305 | 0.7552025 | 0.0009519 | 0.0215920 | TRUE |
| group\_1.69 | 2 | cell\_4 | 3.568861 | 1.2848473 | 0.0009697 | 0.0215920 | FALSE |

Finally, it is important to check whether the data are of high enough quality to apply this method. The metrics discussed in the Supplementary Report to the paper (section *Testing Data Suitability for the Method*) are calculated when the ploidytest object is made. These metrics are:

- `ngenes`: The number of genes that qualify for aneuploidy assessment
- `zeros`: The fraction of 0 counts for each gene that qualifies for aneuploidy assessment
- `residual`: residual score vs. G&T-seq 8-cell stage embryos (see Supplemental Report for details.)

You can access these using the `getMetrics` function:

```
metrics = getMetrics(ploidytest)
#access each one by e.g. metrics$ngenes
```

As an example from this data, the number of genes considered is 3370.

There are two ways to interpret these values: either by direct comparison to the graphs shown in the Supplementary Report (section *Testing Data Suitability for the Method*); or by using the `assessMetrics` function.

Please note that the `assessMetrics` function only provides broad-stroke evaluation of the metrics. There may be other confounding factors in your data that these metrics do not capture: before using the aneuploidy-calling results, you should show to your own satisfaction that you have accounted for these. Finally, `assessMetrics` assumes default parameter choice, particularly for `min.median`.

The output from `assessMetrics` on this data is shown in Table 4

Table 4: Simple quality assessment calls for this dataset

  
|  | result |
| --- | --- |
| residuals | Good quality |
| ngenes | Good quality |
| zeros | Good quality |
