# Supplementary figures and images for: Mosaic autosomal aneuploidies are detectable from single-cell RNAseq data
Source: BMC Genomics. 2017 Nov 25;18:904. doi: 10.1186/s12864-017-4253-x (PMC5702132; doi:10.1186/s12864-017-4253-x)

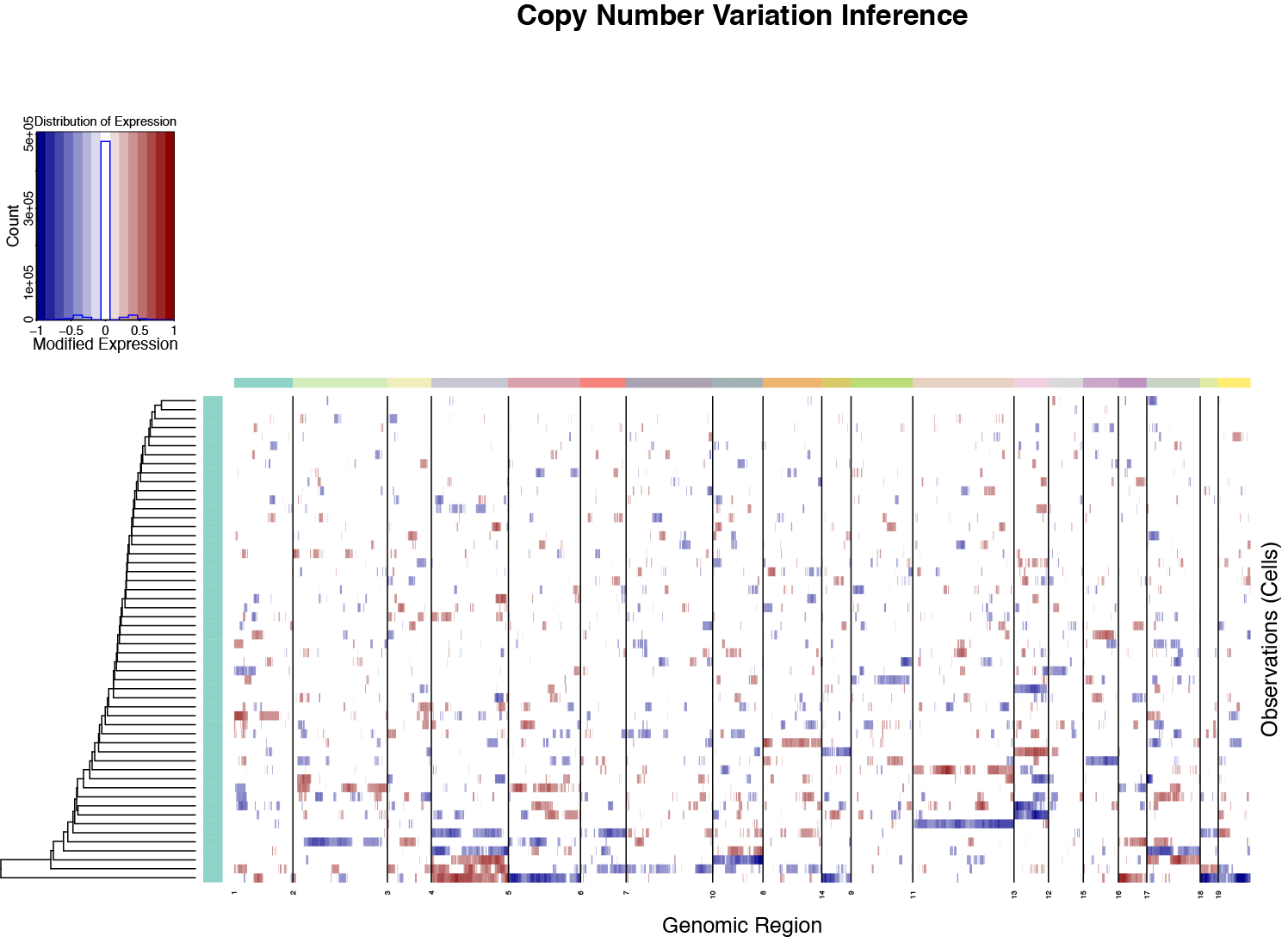

Supplement: Supplementary file 1 — Code for analysis. A gzipped tarball containing all code used for analysis, as well as the.html report referred to above. A package to run aneuploidy assessment in R is also included, alongside a script to download the data we have used. The latest version of these files may be found on https://github.com/MarioniLab/Aneuploidy2017. (GZ 6405 kb) [file 12864_2017_4253_MOESM1_ESM.gz › Aneuploidy2017/infercnv.png]

**Frequency**

0.5

1

1.5

**Chromosome score**

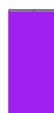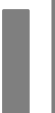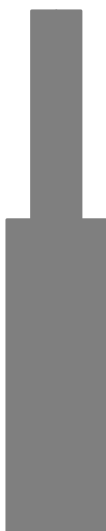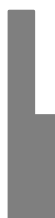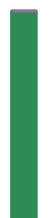

Supplement: Supplementary file 1 — Code for analysis. A gzipped tarball containing all code used for analysis, as well as the.html report referred to above. A package to run aneuploidy assessment in R is also included, alongside a script to download the data we have used. The latest version of these files may be found on https://github.com/MarioniLab/Aneuploidy2017. (GZ 6405 kb) [file 12864_2017_4253_MOESM1_ESM.gz › Aneuploidy2017/plots/1a_schematic.pdf]

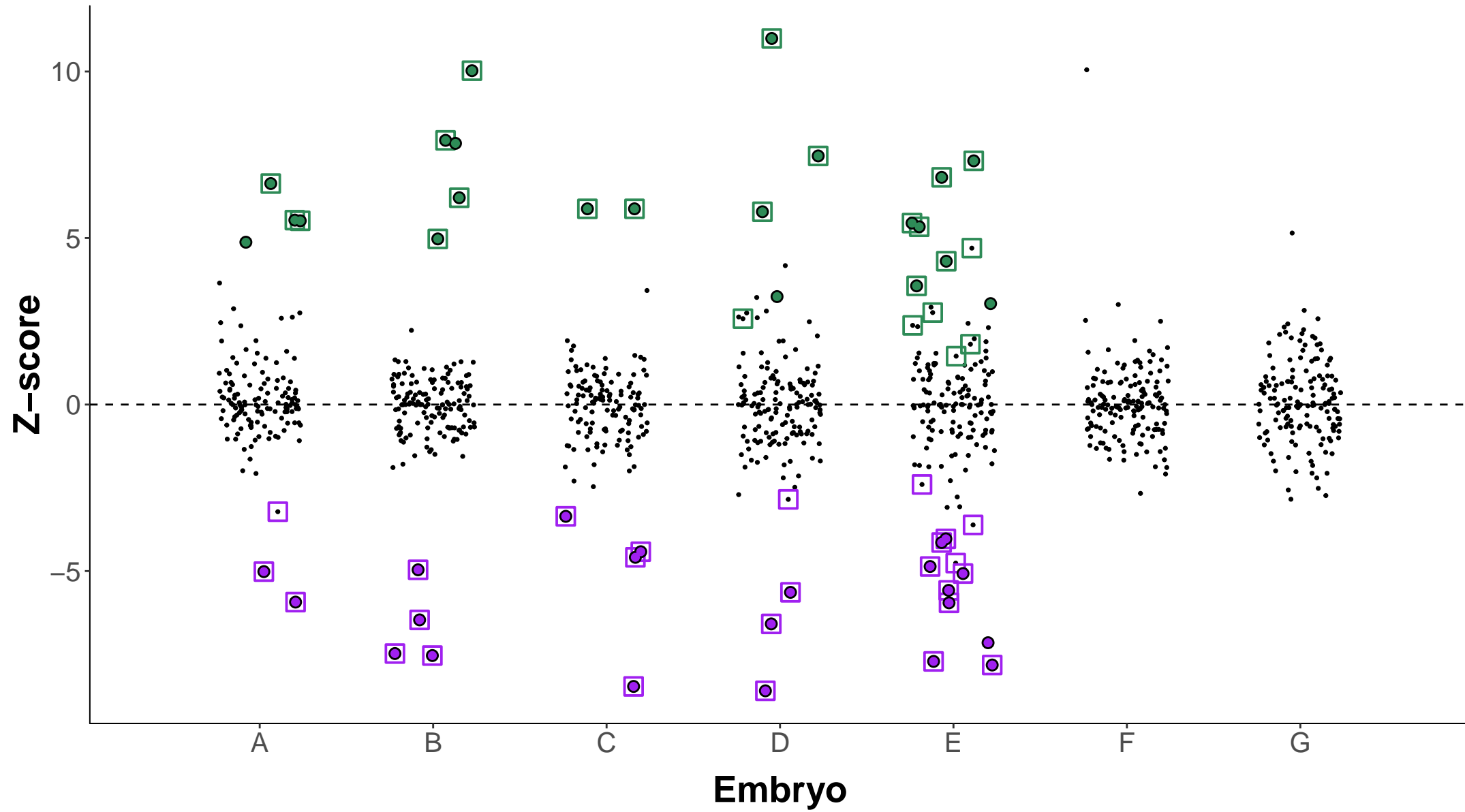

Supplement: Supplementary file 1 — Code for analysis. A gzipped tarball containing all code used for analysis, as well as the.html report referred to above. A package to run aneuploidy assessment in R is also included, alongside a script to download the data we have used. The latest version of these files may be found on https://github.com/MarioniLab/Aneuploidy2017. (GZ 6405 kb) [file 12864_2017_4253_MOESM1_ESM.gz › Aneuploidy2017/plots/1b_8cell.pdf]

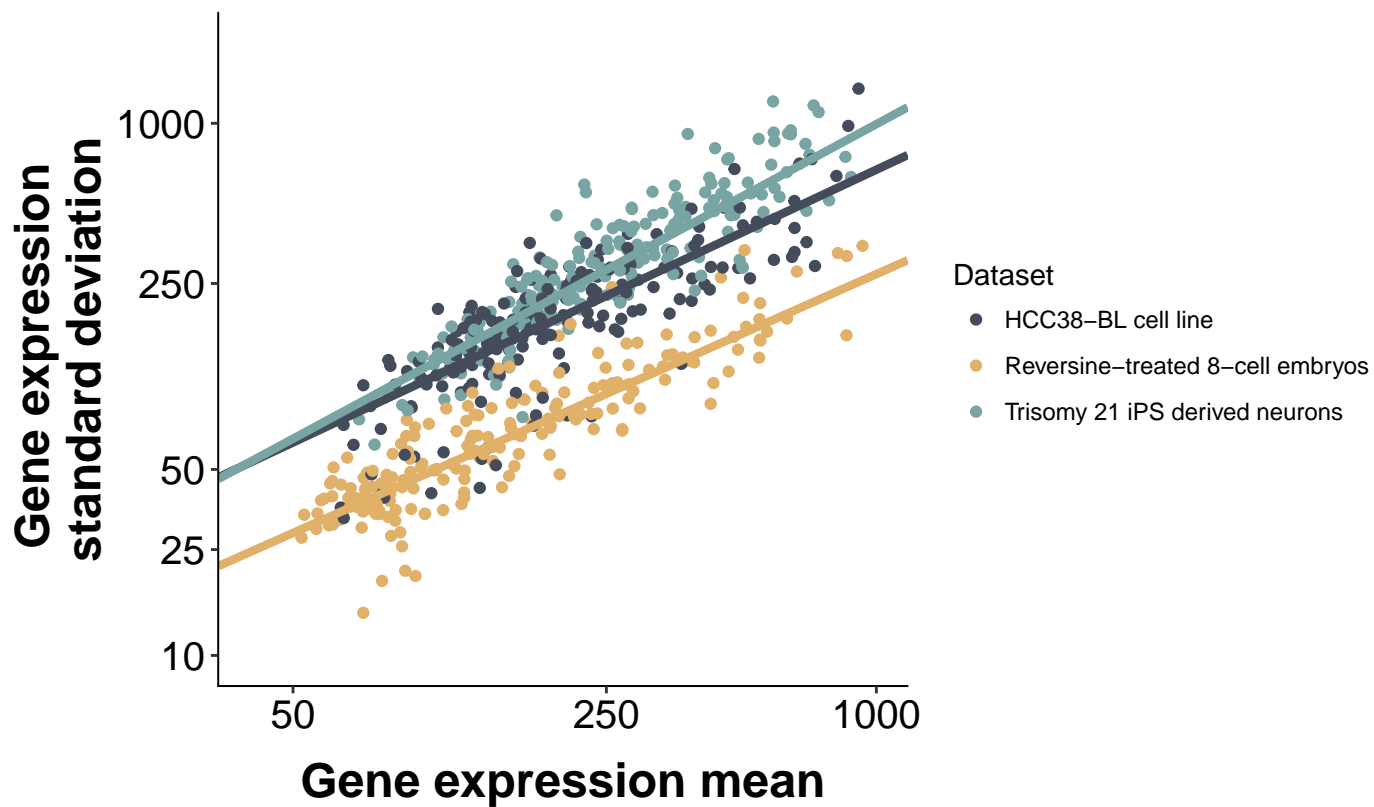

Supplement: Supplementary file 1 — Code for analysis. A gzipped tarball containing all code used for analysis, as well as the.html report referred to above. A package to run aneuploidy assessment in R is also included, alongside a script to download the data we have used. The latest version of these files may be found on https://github.com/MarioniLab/Aneuploidy2017. (GZ 6405 kb) [file 12864_2017_4253_MOESM1_ESM.gz › Aneuploidy2017/plots/2b_dispersion.pdf]

Y

X

Trend fitted from data

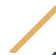

Reversine-treated 8-cell embryos

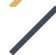

HCC38-BL cell line

Supplement: Supplementary file 1 — Code for analysis. A gzipped tarball containing all code used for analysis, as well as the.html report referred to above. A package to run aneuploidy assessment in R is also included, alongside a script to download the data we have used. The latest version of these files may be found on https://github.com/MarioniLab/Aneuploidy2017. (GZ 6405 kb) [file 12864_2017_4253_MOESM1_ESM.gz › Aneuploidy2017/plots/2c_sim_extralegendonly.pdf]

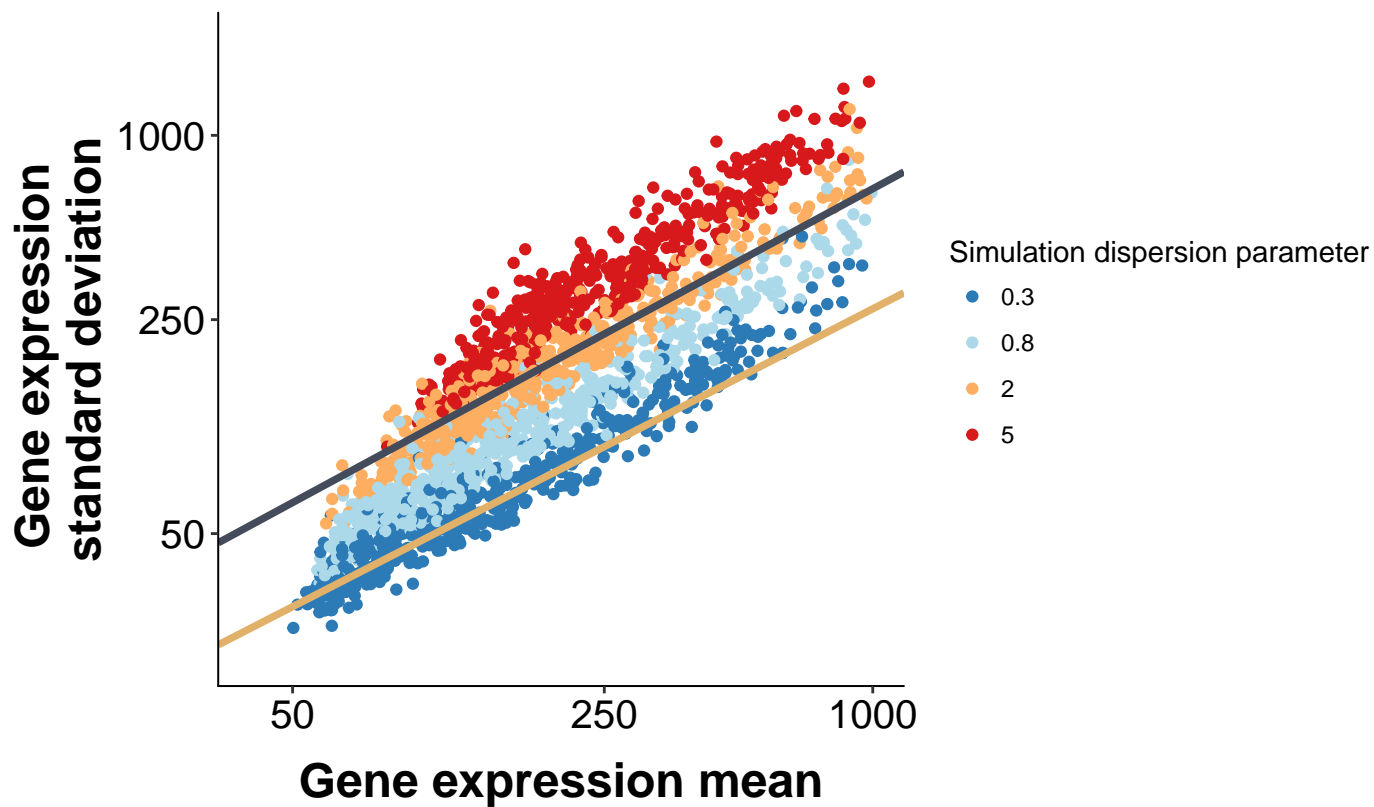

Supplement: Supplementary file 1 — Code for analysis. A gzipped tarball containing all code used for analysis, as well as the.html report referred to above. A package to run aneuploidy assessment in R is also included, alongside a script to download the data we have used. The latest version of these files may be found on https://github.com/MarioniLab/Aneuploidy2017. (GZ 6405 kb) [file 12864_2017_4253_MOESM1_ESM.gz › Aneuploidy2017/plots/2c_simulations.pdf]

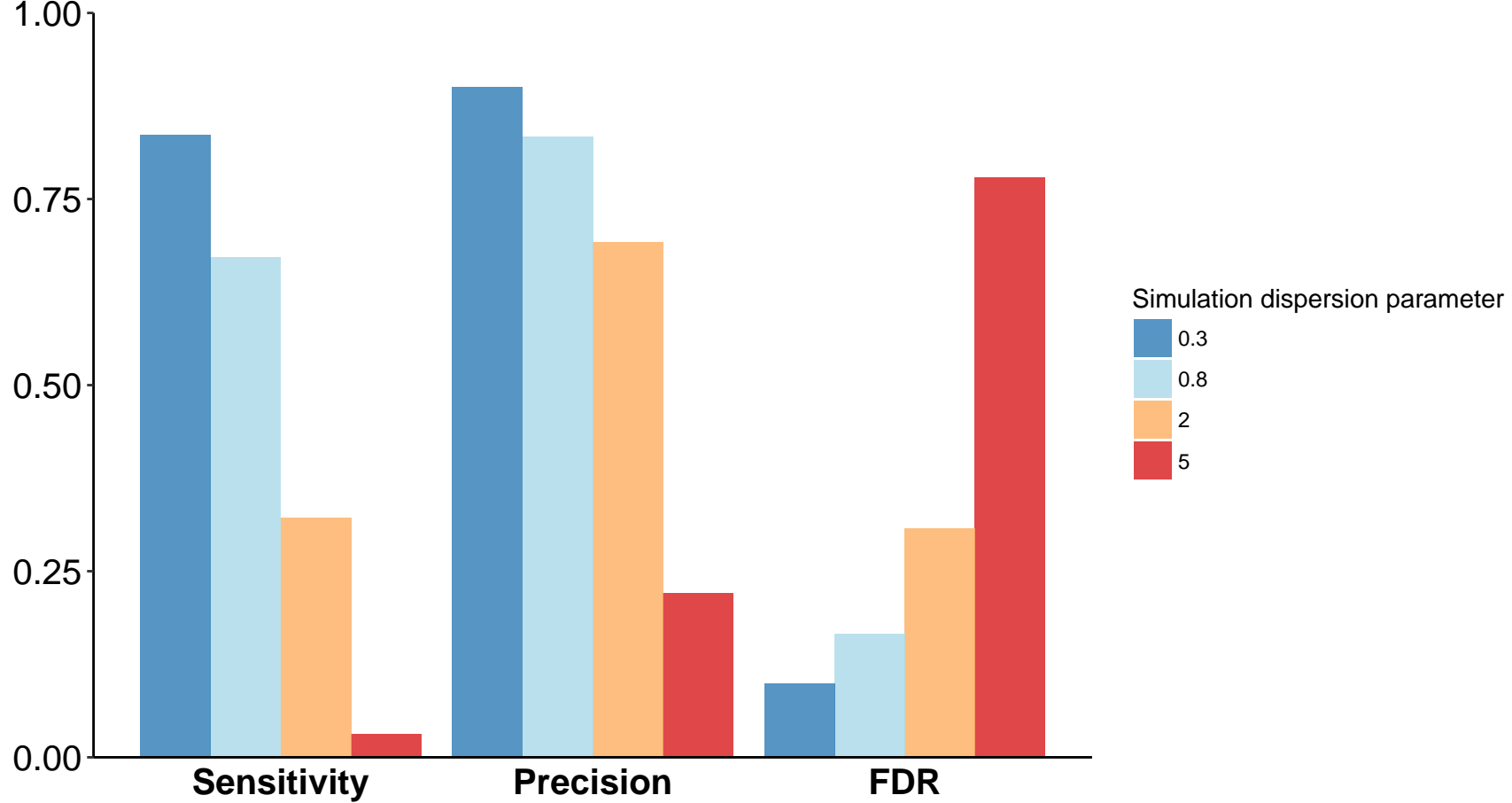

Supplement: Supplementary file 1 — Code for analysis. A gzipped tarball containing all code used for analysis, as well as the.html report referred to above. A package to run aneuploidy assessment in R is also included, alongside a script to download the data we have used. The latest version of these files may be found on https://github.com/MarioniLab/Aneuploidy2017. (GZ 6405 kb) [file 12864_2017_4253_MOESM1_ESM.gz › Aneuploidy2017/plots/2d_sim_result.pdf]

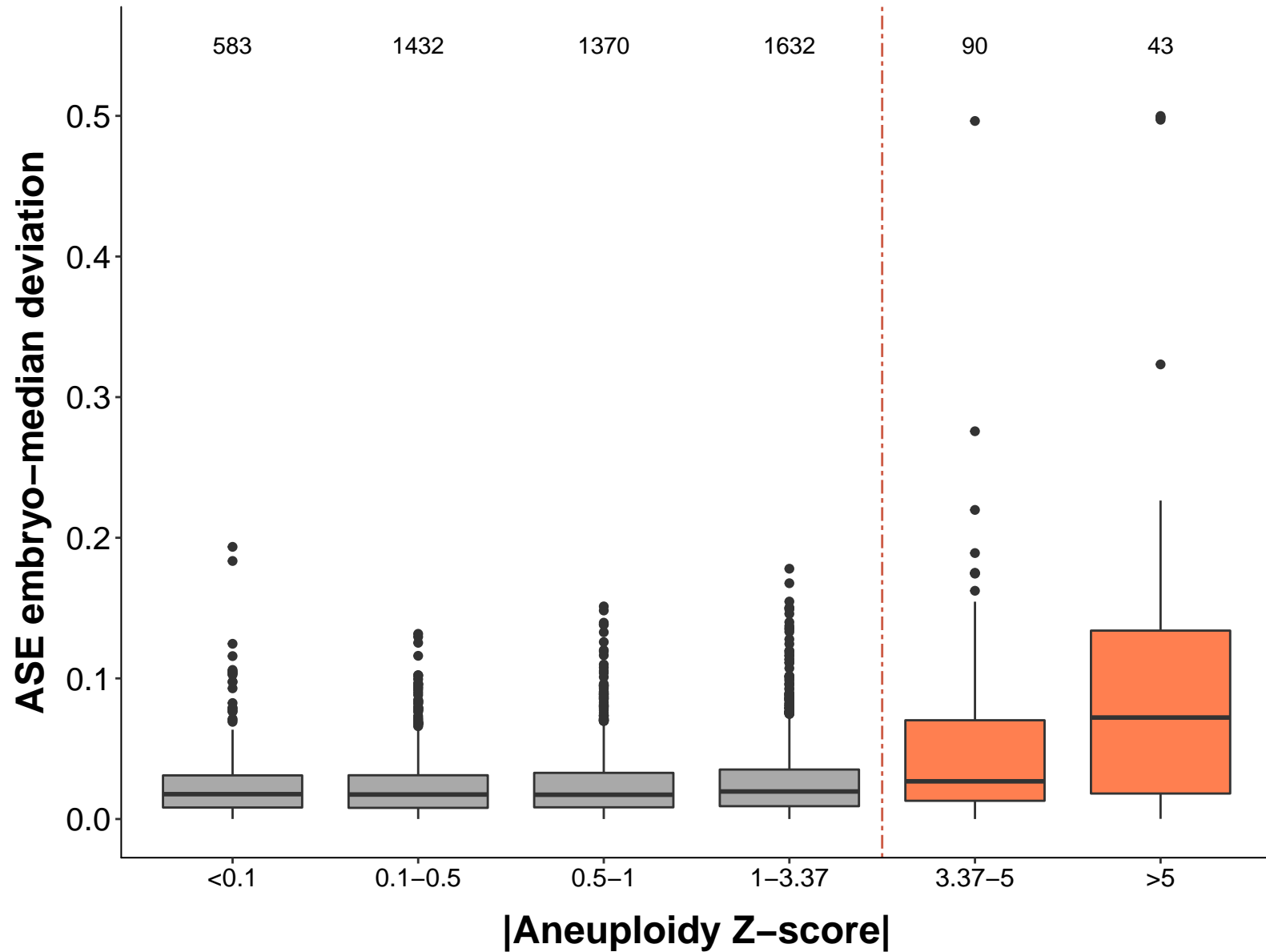

Supplement: Supplementary file 1 — Code for analysis. A gzipped tarball containing all code used for analysis, as well as the.html report referred to above. A package to run aneuploidy assessment in R is also included, alongside a script to download the data we have used. The latest version of these files may be found on https://github.com/MarioniLab/Aneuploidy2017. (GZ 6405 kb) [file 12864_2017_4253_MOESM1_ESM.gz › Aneuploidy2017/plots/3a_deng_score_comparison.pdf]

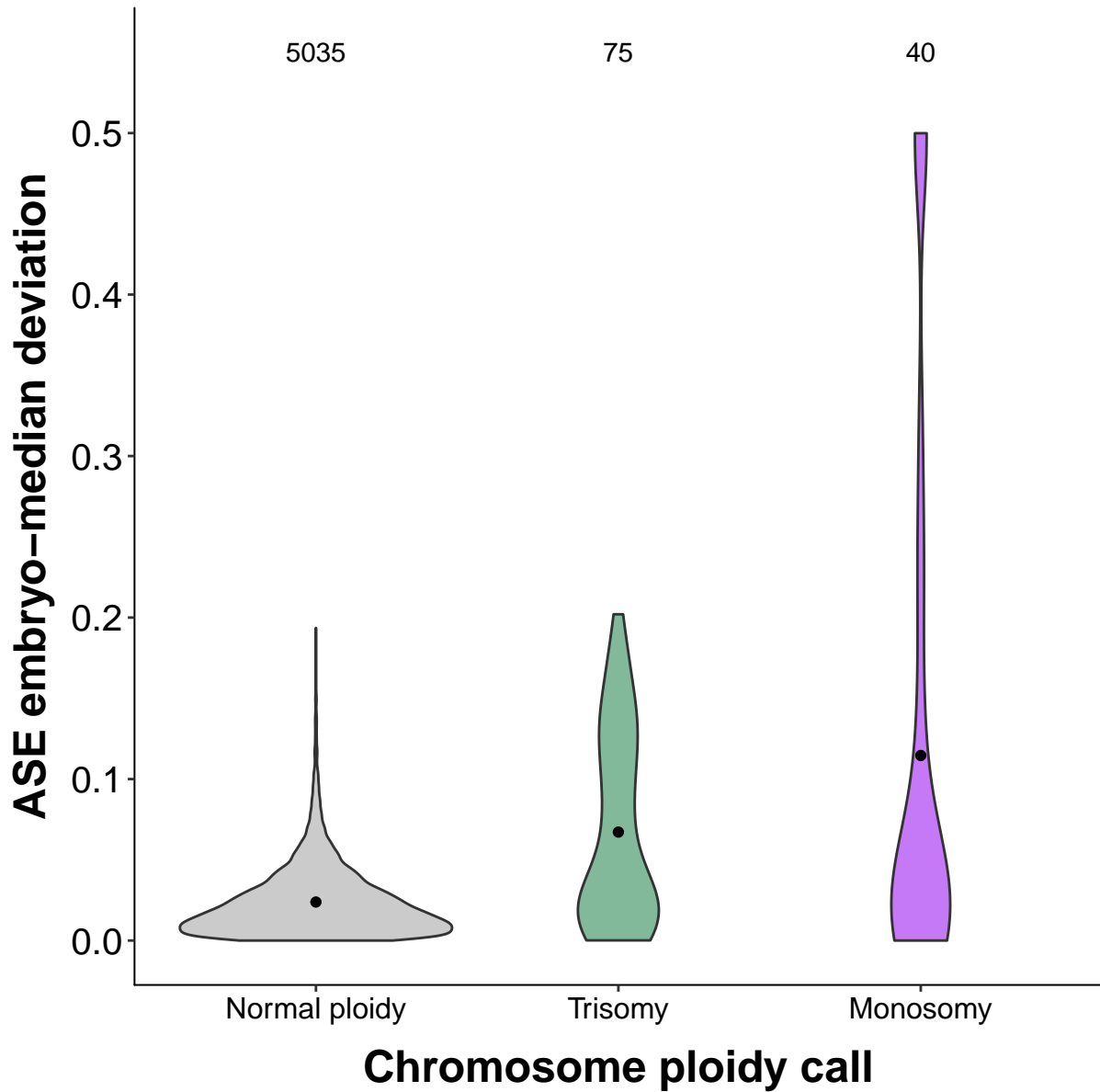

Supplement: Supplementary file 1 — Code for analysis. A gzipped tarball containing all code used for analysis, as well as the.html report referred to above. A package to run aneuploidy assessment in R is also included, alongside a script to download the data we have used. The latest version of these files may be found on https://github.com/MarioniLab/Aneuploidy2017. (GZ 6405 kb) [file 12864_2017_4253_MOESM1_ESM.gz › Aneuploidy2017/plots/3b_deng_call_ase.pdf]

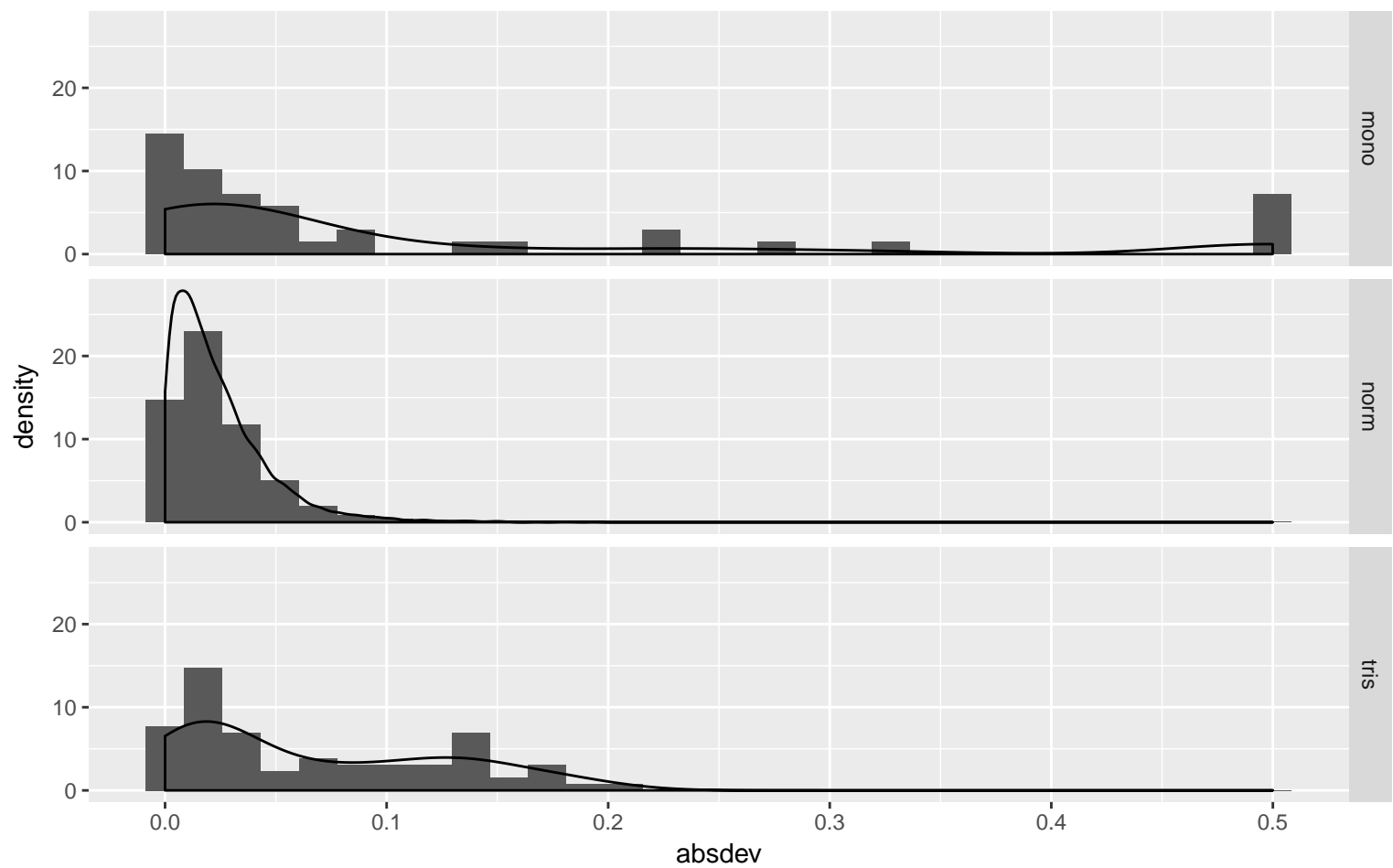

Supplement: Supplementary file 1 — Code for analysis. A gzipped tarball containing all code used for analysis, as well as the.html report referred to above. A package to run aneuploidy assessment in R is also included, alongside a script to download the data we have used. The latest version of these files may be found on https://github.com/MarioniLab/Aneuploidy2017. (GZ 6405 kb) [file 12864_2017_4253_MOESM1_ESM.gz › Aneuploidy2017/plots/alt_3b.pdf]

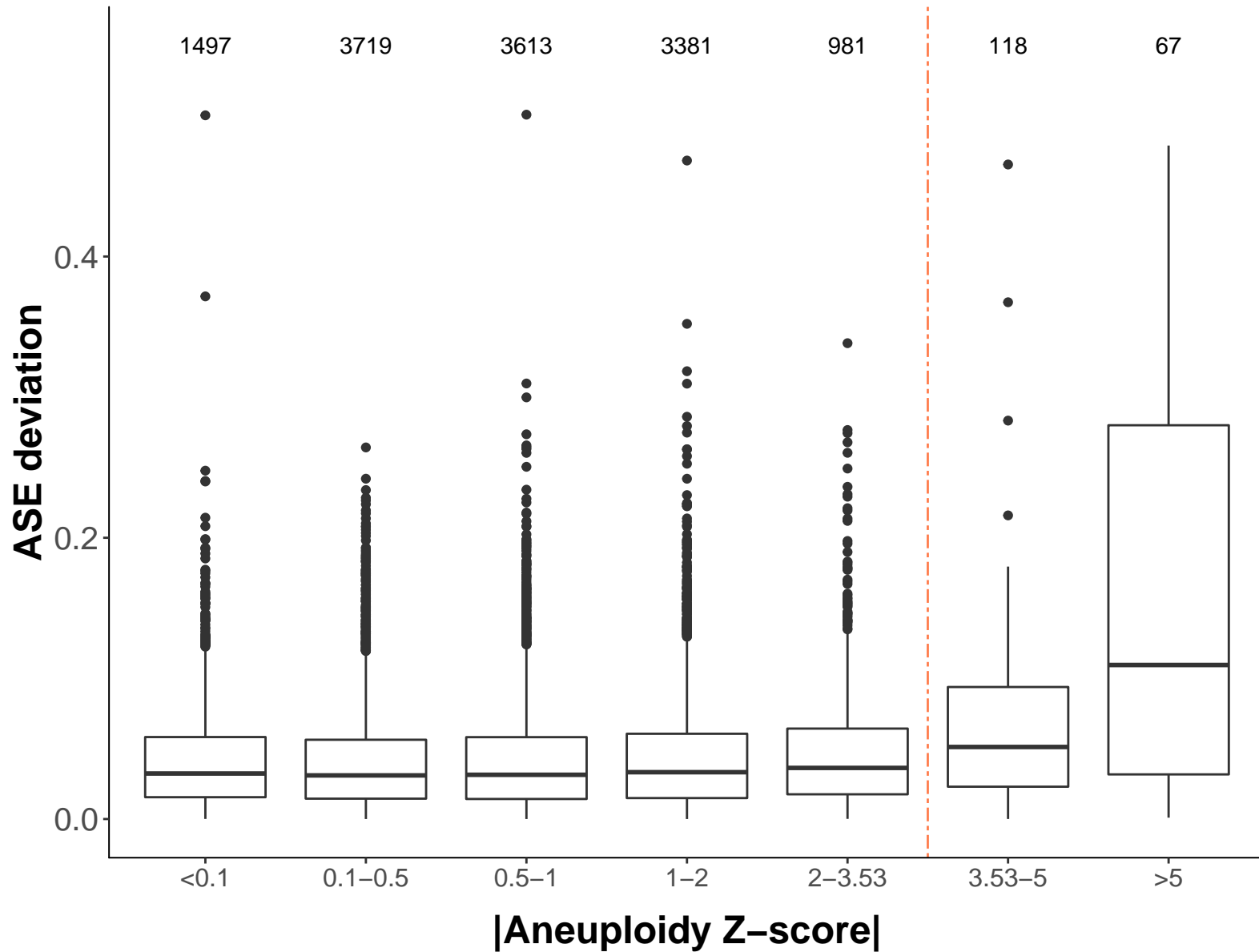

Supplement: Supplementary file 1 — Code for analysis. A gzipped tarball containing all code used for analysis, as well as the.html report referred to above. A package to run aneuploidy assessment in R is also included, alongside a script to download the data we have used. The latest version of these files may be found on https://github.com/MarioniLab/Aneuploidy2017. (GZ 6405 kb) [file 12864_2017_4253_MOESM1_ESM.gz › Aneuploidy2017/plots/S1_mesc_score_comparison.pdf]

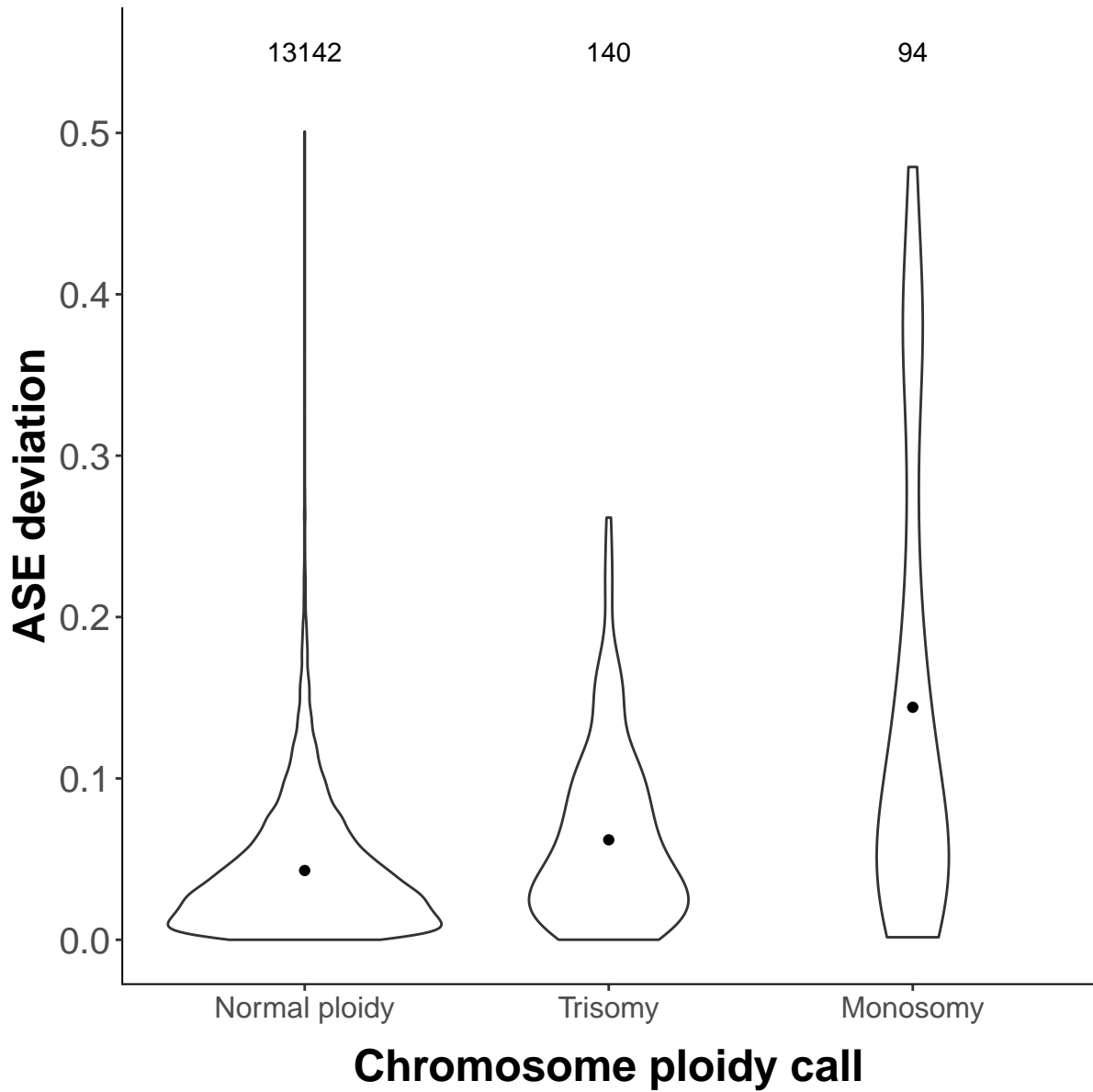

Supplement: Supplementary file 1 — Code for analysis. A gzipped tarball containing all code used for analysis, as well as the.html report referred to above. A package to run aneuploidy assessment in R is also included, alongside a script to download the data we have used. The latest version of these files may be found on https://github.com/MarioniLab/Aneuploidy2017. (GZ 6405 kb) [file 12864_2017_4253_MOESM1_ESM.gz › Aneuploidy2017/plots/S2_mesc_call_ase.pdf]
